# Recovery Trends in Marine Mammal Populations

**DOI:** 10.1371/journal.pone.0077908

**Published:** 2013-10-30

**Authors:** Anna M. Magera, Joanna E. Mills Flemming, Kristin Kaschner, Line B. Christensen, Heike K. Lotze

**Affiliations:** 1 Department of Biology, Dalhousie University, Halifax, Nova Scotia, Canada; 2 Department of Mathematics and Statistics, Dalhousie University, Halifax, Nova Scotia, Canada; 3 Department of Biometry and Environmental System Analysis, University of Freiburg, Freiburg, Germany; 4 CESAB (Centre de Synthèse et d′Analyse sur la Biodiversité), Immeuble Henri Poincaré, Domaine du Petit Arbois, Aix-en-Provence, France; 5 Fisheries Centre, University of British Columbia, Vancouver, British Columbia, Canada; Aristotle University of Thessaloniki, Greece

## Abstract

Marine mammals have greatly benefitted from a shift from resource exploitation towards conservation. Often lauded as symbols of conservation success, some marine mammal populations have shown remarkable recoveries after severe depletions. Others have remained at low abundance levels, continued to decline, or become extinct or extirpated. Here we provide a quantitative assessment of (1) publicly available population-level abundance data for marine mammals worldwide, (2) abundance trends and recovery status, and (3) historic population decline and recent recovery. We compiled 182 population abundance time series for 47 species and identified major data gaps. In order to compare across the largest possible set of time series with varying data quality, quantity and frequency, we considered an increase in population abundance as evidence of recovery. Using robust log-linear regression over three generations, we were able to classify abundance trends for 92 spatially non-overlapping populations as Significantly Increasing (42%), Significantly Decreasing (10%), Non-Significant Change (28%) and Unknown (20%). Our results were comparable to IUCN classifications for equivalent species. Among different groupings, pinnipeds and other marine mammals (sirenians, polar bears and otters) showed the highest proportion of recovering populations, likely benefiting from relatively fast life histories and nearshore habitats that provided visibility and protective management measures. Recovery was less frequent among cetaceans, but more common in coastal than offshore populations. For marine mammals with available historical abundance estimates (n = 47), larger historical population declines were associated with low or variable recent recoveries so far. Overall, our results show that many formerly depleted marine mammal populations are recovering. However, data-deficient populations and those with decreasing and non-significant trends require attention. In particular, increased study of populations with major data gaps, including offshore small cetaceans, cryptic species, and marine mammals in low latitudes and developing nations, is needed to better understand the status of marine mammal populations worldwide.

## Introduction

In the marine realm, mammals appear to be one of the taxa that have benefitted the most from a shift from resource exploitation toward wildlife conservation [1]–[4]. Marine mammals, a loose grouping of approximately 127 species, include cetaceans (whales, porpoises and dolphins), pinnipeds (true seals, fur seals and sea lions), as well as marine otters and sea otters, sirenians (manatees and dugongs) and polar bears [5]. Throughout history, humans have exploited and often depleted marine mammal populations [3], [6]–[13]. Yet in the 20^th^ century, substantial population declines led to relatively early and widespread reduction or cessation of commercial exploitation and implementation of conservation measures [1], [2], [4], [14]. While several marine mammals have been held up as key conservation success stories (e.g. eastern North Pacific gray whales, *Eschrichtius robustus* [15], [16] and sea otter, *Enhydra lutris,* populations [17]), not all marine mammal populations have recovered from earlier exploitation-driven declines. However, with data indicating positive abundance trend estimates for various populations, we now have the opportunity to not only evaluate threat status, decline, and extinction risk in marine mammals, but also increase and recovery.

Threats to marine mammals are numerous and have changed over time. Historically, marine mammals have been prized sources of meat, oil, fur, baleen and ivory [6], [7], [9], [11]. They have also been captured for display in aquariums, culled when declared nuisances, used for bait, and indirectly exploited as bycatch [18]–[24]. Numerous marine mammal species were reduced to very low abundances by or during the 1900 s [2], [7], [25], and some almost to extinction, such as Northern elephant seals (*Mirounga angustirostris*) [26] and Guadalupe fur seals (*Arctocephalus townsendi*) [27]. Certain populations were regionally extirpated, including the sea otter throughout most of its range [17], the Atlantic gray whale, and the walrus (*Odobenus rosmarus*) in parts of the Northwest Atlantic [1]. A handful of species have become globally extinct, including the Steller’s sea cow (*Hydrodamalis gigas*), sea mink (*Neovison macrodon*), Caribbean monk seal (*Monachus tropicalis*), and Japanese sea lion (*Zalophus japonicu*s) [5], [26]. Among the survivors, however, some substantial population recoveries have occurred, for example for gray whales in the eastern North Pacific, multiple populations of humpback whales (*Megaptera novaeangliae*), southern right whales (*Eubalaena australis*) [28], sea otters [29], northern elephant seals [26], grey seals (*Halichoerus grypus*) in the UK [30] and Northwest Atlantic [31], and numerous fur seal species [27]. Yet direct and indirect exploitation, as well as disease, competition for prey, habitat degradation from coastal development and dams, ship traffic, offshore oil and gas exploration, pollution (chemical, physical and acoustic), and climate change continue to impact marine mammal populations today [23], [24], [32]–[34].

Although it is a commonly used term, “recovery” can have many definitions in different management and conservation contexts [25], [35]. What definition of recovery is chosen depends on the goals of the study and can alter the conclusions. Generally, recovery is taken to be “a return to a normal state of health… or strength” [36]. In analyses of wild animal populations, however, we often do not know the “normal state” of a population [25]. Pre-commercial exploitation abundance or carrying capacity (K) can be used as a reference point, with the increase of a population towards such a reference point indicating recovery [6], [25], [37]. However, neither pre-exploitation nor K estimates exist for many species that lack records or data on past catches, traded products (e.g. oil, fur), scientific surveys, genetics, life history, or population structure. Furthermore, there is often debate as to whether K estimates should refer to pre-exploitation or current ecosystem conditions [6], [38]. Therefore, a basic and practical approach in many data-limited (in terms of time span, number, frequency, and precision of data points) cases has been to view any significant abundance increase as evidence of a recovering population and at least partial recovery [25], [39]. This is the definition we used in this study as it allowed us to compare across a maximal number of marine mammal population abundance time series with variable data quality.

Management bodies often judge recovery with respect to a proportion of K or pre-exploitation size. The U.S. Marine Mammal Protection Act specifies management for an “optimal sustainable population” level, which is defined by the U.S. National Marine Fisheries Services (NMFS) as “a population level between carrying capacity and the population size at maximum net productivity” [37]. An optimal sustainable population level for marine mammals is thought to be between 50–85% of K [37], but generally 60% is used [15], and the International Whaling Commission assumes 60% is the level at which whale populations are most productive [28]. In the absence of either K or pre-exploitation abundance, management bodies may use maximum observed population levels, based on survey data for example, as a reference point. Such is the case with Atlantic seal populations in Canada [40]. However, in cases where reports of substantial declines predate quantitative records, maximum observed population levels would not represent pre-exploitation levels, and could result in the underestimation of declines and the overestimation of recoveries. Additional criteria, such as targets for demographic features (e.g. ratios of juveniles to adults, or males to females), social dynamics, or ecological functions [41] may be relevant to evaluating recovery depending on the situation. Recovery may also be measured over different time periods, such as an entire time series of data, a set time period (e.g. 50 years, or 1950–2000), or with respect to the species’ life history (e.g. three generations is commonly used by the International Union for Conservation of Nature (IUCN) [42]).

Despite the strong conservation focus on marine mammal management in many parts of the world, abundance data are limited and subject to high uncertainty. Reliable, effort-corrected catch data are absent for many populations, and marine mammals are notoriously difficult to survey accurately for abundance. Current population designations and/or distributions may not match historical records, leading to speculation that some populations have changed their distributions over time [43], [44], and complicating assessments of long-term trends. Longer time series with historical population estimates are lacking for many populations that are or were not commercially valuable [2]. Many marine mammal species have elusive behaviors (e.g. extensive migrations or deep diving for prolonged time periods) which, combined with their widespread occurrences in remote areas, represent high logistical and economic challenges for monitoring their population statuses [15], [45]–[47]. With the exception of land-breeding pinnipeds, accurate estimation of abundance trends over relatively short time periods is difficult in many cases [46], and abundance time series often encompass irregular survey intervals. Existing variability in survey coverage and methodology greatly hampers the assessment of population trends [47]. However, data availability has improved greatly with the population monitoring and modeling that have accompanied increased management and conservation efforts internationally and domestically since the 1970 s.

Although marine mammal abundance data are collected and assessed by several organizations for management and conservation purposes (e.g. IUCN, U.S. marine mammal stock assessments), a quantitative global synthesis of marine mammal recoveries on a population level has not been previously attempted. Populations within the same species may show different abundance trends, and population-level abundance monitoring has become increasingly valued. Thus, the objectives of our study were to compile publicly available population-level abundance time series for marine mammals around the world, assess population trends, and classify recovery. Where possible, we also aimed to quantify the relationship between historical population decline and subsequent recovery. Our overall goal was to enhance our understanding of recovery in formerly exploited marine mammals and marine species overall.

## Materials and Methods

### Data Compilation

We expanded a database of marine mammal abundance estimates collected by Kaschner (2004) [48] and Christensen (2006) [11]. We collected population-level abundance data from around the world from publicly available published journal articles, online government documents, stock assessment reports, and recovery plans. Major sources included: Fisheries and Oceans Canada (DFO), Committee on the Status of Endangered Wildlife in Canada (COSEWIC), U.S. National Oceanic and Atmospheric Administration (NOAA) and NMFS technical and administrative reports, U.S. Marine Mammal Stock Assessment Reports, St. Andrew’s Sea Mammal Research Unit reports, Australian and New Zealand government documents, International Whaling Commission documents, and numerous published primary sources (Table S1). We collected data up to 2008 or the next most recent data point. We also collected information on generation times, methods of data collection and abundance estimation, and data reliability. For abundance trend analysis, we limited our attention to time series with at least three estimates. Although a population is generally described as a group of interbreeding organisms of the same species in a defined area [49], for the purpose of this study we chose population abundance data according to consistently defined areas described in the source literature. Thus, we did not exclusively adhere to population or stock definitions from monitoring or regulatory agencies, but the population designations often matched.

Population abundance data came from dedicated and opportunistic aerial, land-based and ship-based surveys, extrapolated total population or pup-count data, photo-identification and mark-recapture models, genetic diversity analysis, combined totals from the literature, models that relied heavily on bycatch, catch or catch-per-unit-effort data, various other model-derived estimates from these aforementioned types of data (including age- or stage-based, simple regression, Bayesian or state-space models), and in some cases unknown or unstated methods (Table S1). When year ranges were provided for abundance estimates, we used the mid-point of the range.

Error is present in virtually all marine mammal abundance data, but it may originate from different sources. For example, catch, bycatch, or product data could be subject to intentional or unintentional misreporting. In turn, abundance estimation from inaccurate data may provide faulty estimates of K or the intrinsic rate of increase (r_max_), both of which may vary over time [6]. Historical abundance estimates from DNA have been questioned because of uncertainty over changes in mutation rates, appropriate designation of particular populations, and migration, all of which can affect population estimates [38]. Survey data are affected by biases such as avoidance or attraction behavior of the species surveyed, and estimates may also be skewed because of detectability issues, particularly for more cryptic species. Analysis methods have been developed, however, to correct survey data for unobserved animals that may be at sea or are otherwise not visible [45], [50], [51].

We collected any reported error information (e.g. coefficient of variation, confidence intervals, standard error, or standard deviation). Where available, we gathered pre-exploitation abundance estimates and K estimates. In cases where multiple historical estimates were found (e.g. catch vs. genetic data) we recorded them all. Not all data sources reported error, so we used a system to incorporate quantitative error information (where available) with qualitative information about the data source for each data point and its reliability into abundance trend estimation (Abundance Confidence ID (ACID), Text S1).

### Data Analysis – Trend Overview

We used a general definition of recovering populations being those that displayed an increase in abundance. Our main objectives were to gather abundance data and statistical evidence so as to broadly classify populations as having either increasing or decreasing trends, rather than attempting to estimate and describe complex population trajectories. In order to do so we estimated a trend for each population over the three most recent generations. This corresponded with the criteria used by the IUCN for assessing population decline [42]. In cases where we did not have data for the entire three-generation time period, we used the available time period with a required minimum of ten years instead (i.e. the minimum time period used by the IUCN for assessing population declines [52]) (Table S2).

### Trend Analysis

Given available data and our aim, we used robust regression to estimate population trends. Robust regression is a powerful tool for fitting linear models that simultaneously identifies and down weights outlying data points [53], [54]. Specifically we used the lmRob command in the robust library in R (which makes available an S-estimator [55] with a high breakdown point). We performed a full exploratory analysis using simple and robust linear regression, as well as log-linear robust regression (i.e. with a log transformation of the abundance values) and with and without weighting to include abundance data error information. We fit simple and robust regressions to both regular abundance data (i.e. number of individuals) and standardized abundance data (obtained by subtracting the mean from each data point and dividing by the standard deviation). The standardization facilitated comparison of regression results amongst different populations. We decided to use robust linear and robust log-linear weighted regressions as they best reflected the data. The complete R code is available in Text S2.

Results of fitting the linear and log-linear robust weighted regressions were then used to classify each population as Significantly Increasing, Significantly Decreasing, Non-Significant Change, or Unknown. To start, any populations with insufficient data (i.e. <3 data points over three generations or minimum 10 years) were deemed Unknown. Those remaining were classified according to both the direction and significance of their abundance trend. Specifically, Significantly Increasing and Significantly Decreasing populations had positive or negative slope estimates, respectively, and 95% confidence intervals that did not include zero. Populations with 95% confidence intervals that did include zero were classified as showing Non-Significant Change. Our classifications of population trends allowed for an easily interpretable summary of patterns in the marine mammal population abundance data.

### Trend Comparisons

We examined both the population trend estimates (and the ensuing classifications) for marine mammals overall, as well as for notable taxonomic divisions (cetaceans, pinnipeds, other marine mammals), main habitat type according to Jefferson et al. (2008) [5] (for cetaceans only: coastal, offshore, or both), and other relevant sub-groupings (e.g. mysticetes or baleen whales, odontocetes or toothed whales, a sub-group of just dolphins and porpoises, otariids or eared seals, phocids or true seals). The groupings were not necessarily mutually exclusive. For example, a pantropical spotted dolphin (*Stenella attenuata*) population would be included in the overall marine mammal category, as well as the cetacean, odontocete, and dolphin and porpoise sub-groupings. The groupings for each population are included in Table S2. Many of the populations for which time series data exist overlapped spatially or were nested in each other’s range, thus likely representing a similar set of individual animals. To avoid such double counting, we grouped populations into the largest and smallest non-overlapping or non-nested areas and analyzed both sets separately. These groupings by large and small areas are also listed in Table S2.

### Comparison with IUCN Data

We compared our population trend classifications (Significantly Increasing, Significantly Decreasing, Non-Significant Change, Unknown) to those available through the IUCN Red List (Increasing, Decreasing, Stable, Unknown) (www.iucnredlist.org, Table S3). Since our data were mostly collected at a population level, and the IUCN mostly works with species level, we first summarized our data at a species level to facilitate comparison. For each species, we selected from our database those populations that comprised the majority of the overall species abundance. In cases of multiple non-nested and approximately equal-sized populations, we took the most frequently occurring trend classification of the populations of the species. If this did not exist (e.g. as for three populations with different trend classifications), we listed the population classification for that particular species as Unknown.

### Historical Declines and Recent Increases

With the available data, we also had the opportunity to examine another facet of marine mammal recovery. For some populations (n = 47), we had at least one historical estimate, as well as a minimum population estimate and a current population estimate that was above the minimum estimate. Thus, these populations demonstrated both a historical decline (decrease) and evidence of recent recovery (increase) (Table S4). We were interested in whether the magnitude of recent recovery was related to the magnitude of historical decline. Therefore, the historical abundance estimate(s) was compared to the population minimum and the most recent abundance estimates to estimate the magnitude of decline and the magnitude of recovery with respect to the historical population size. For the populations for which there existed multiple historical abundance estimates, we used the mean of the declines and recoveries relative to these historical estimates. In addition to the magnitude of recovery, we were also interested in comparing the magnitude of historical population declines to the rates of recovery (i.e. the slopes of our above regression analysis). Unfortunately, only a small subset of the populations with historical abundance estimates had enough data over three generations to derive a rate of recovery, which provided too small a sample size from which to derive meaningful patterns.

## Results

### State of Available Marine Mammal Population Data

Overall, we were able to compile 182 population abundance time series for 47 species of marine mammals (i.e. 37% of the 127 currently recognized marine mammal species) for which there were at least three abundance estimates over three generations or at least ten years. Originally, we had compiled 198 population abundance time series; however, some time series corresponded to the same population, representing both pup count and regular count (i.e. entire population) data. We used the regular data unless they were much sparser than the pup count data. With these duplicate population indices removed, we had 182 population abundance time series in total.

**Figure 1:**
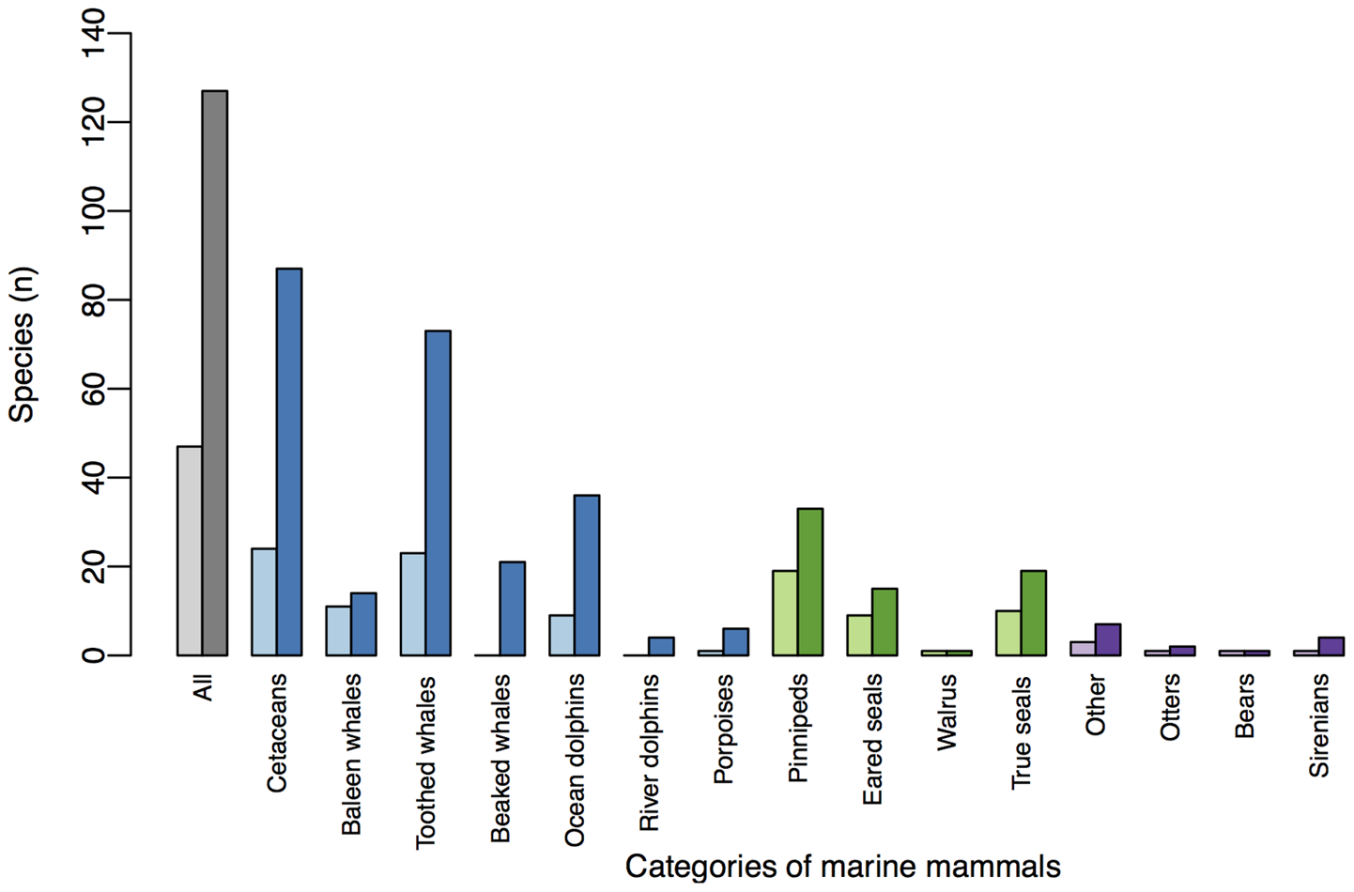
Species represented in population-level data in this study compared to marine mammal species overall. This study = light bars, marine mammals species overall = dark bars. Colors represent main taxonomic groups and their relevant sub-groupings: all marine mammals (grey), cetaceans (blue), pinnipeds (green), and other marine mammals (marine and sea otters, polar bears and sirenians) (purple).

A breakdown of species by taxonomic groups and different sub-groupings represented in this study compared to all marine mammals is depicted in Figure 1. Groups with high representation (>50% of known species represented) were the baleen whales (mysticetes) and pinnipeds. Groups with low representation in our data (<50% of known species represented) included the sirenians and cetaceans overall, especially toothed whales (odontocetes) within the cetaceans grouping, and dolphins and porpoises within the toothed whales grouping. No species of beaked whales (n = 21 species globally) or river dolphins (n = 4 species globally) were included. It was difficult to obtain time series that met our criteria for many smaller cetacean species (notably porpoises, beaked whales and river dolphins), Antarctic true seals, and sirenians. However, we were able to obtain three-generation abundance data (rather than the 10 years of data) for 95 of the 182 populations.

### Marine Mammal Population Trends

**Figure 2:**
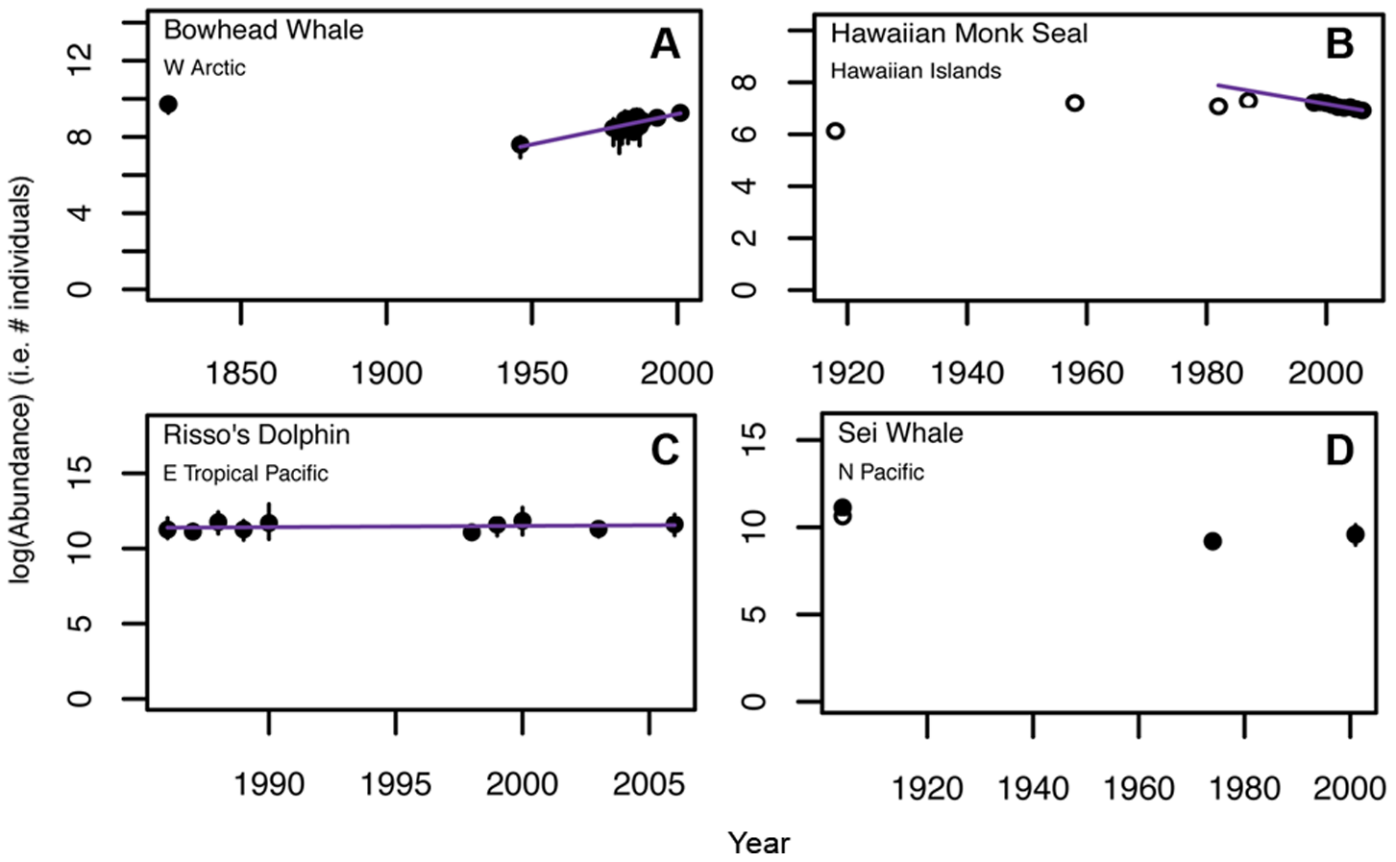
Examples of Significant Increase (A), Significant Decrease (B), Non-Significant Change (C) and Unknown (D) population abundance trends. Robust weighted log-linear regression line is depicted over three generations or at least ten years. Solid points = abundance estimates with reported error (95% confidence intervals). Open points = abundance estimates without reported error.

**Figure 3:**
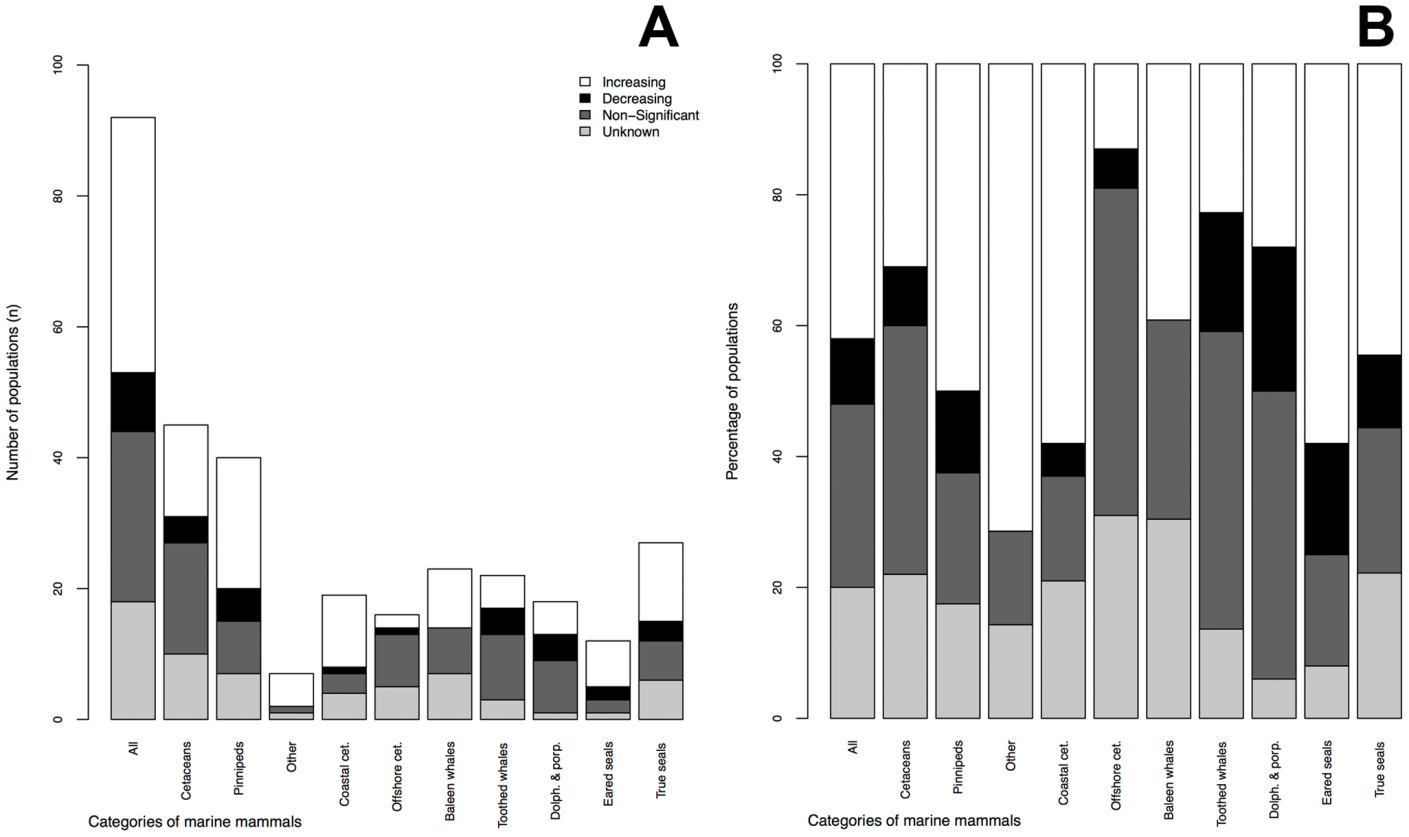
Trend classification for marine mammal populations by different categories as numbers (A) and proportions (B). Summary of results from robust weighted log-linear regressions for 92 (non-nested, including the largest possible areas) marine mammal populations. “Other” includes sirenians, polar bears and sea otters.

Population trends were estimated for 182 non-duplicated populations from 47 species (see Figure 2 for examples, Figure 3 for population trend classifications, Figure S1 for plots for all populations, and Table S5 for all regression results). The models that best fit the available data were found to be those based on scaled abundance data and estimated via robust log-linear regression (weighted by ACID). We compared the R-squared values for both the robust linear and log-linear models, and found that robust log-linear models provided a better fit to the data for 78 of the populations, while robust linear models were better for 50 of the populations. There was 1 population for which both models produced a similar result, and 33 populations with Unknown trends. However, the resulting trend classifications were largely the same, with the two models producing different results for only 20 populations. In these cases, the robust log-linear model provided a better fit for 13 populations, and for the other 7 populations it found no evidence of trend (i.e. Non-Significant Change) as opposed to a Significantly Increasing or Significantly Decreasing trend provided by the better fitting robust linear model. Since the robust log-linear model best described the data in the majority of cases, we reported these results in the paper. However, plots and results for both the robust linear and log-linear regressions are included in the supplementary materials (Figure S1 and Table S5).

In terms of marine mammal population trends for the largest non-overlapping areas (n = 92 populations), 42% were Significantly Increasing, 10% were Significantly Decreasing, 28% showed Non-Significant Change, and 20% were deemed Unknown (see ‘All’ in Figure 3). Since some populations were nested within each other and consequently not independent, we analyzed trends for populations chosen by the smallest (n = 135) and the largest (n = 92) non-overlapping areas. Results did not differ substantially so we report results by the largest non-overlapping areas. The number of other marine mammal populations (polar bears, otters and sirenians) in the analysis was low (n = 7), with sea otters comprising the majority (n = 4) of the sample of other marine mammals.

Some populations did have flat slopes (i.e. very close to zero) and small confidence intervals that might suggest stable population abundance, such as the New Zealand sea lion (*Phocarctos hookeri*) (Sandy Bay) and the Southern elephant seal (*Mirounga leonina*) (Kerguelen Isles) (Figure S1). However, they did not have significant trends and rather than include an additional trend classification category, they were grouped with other populations that had larger confidence intervals and/or more variable data as displaying Non-Significant Change.

When comparing different categories of marine mammals (Figure 3), our results indicate that proportionally more sirenian, polar bear and sea otter populations (i.e. “Other”, 71%) and pinnipeds (50%) were Significantly Increasing, than marine mammals overall (42%) or cetaceans (31%). For the pinnipeds, eared seals (58%) showed more Significantly Increasing populations compared to true seals (44%). Among the cetaceans, all taxonomic groups showed relatively low numbers of Significantly Increasing populations (39% baleen whales, 23% all toothed whales, 28% just dolphins and porpoises). Primarily coastal cetaceans, however, had proportionally more Significantly Increasing populations (58%) than primarily offshore cetaceans (13%). Overall, pinnipeds (and especially eared seals), other marine mammals, and coastal cetaceans showed the highest proportions of Significantly Increasing populations. Toothed whales, and dolphins and porpoises, had the highest proportions of Significantly Decreasing populations. All categories of cetaceans, except for coastal cetaceans, had approximately one third or more Non-Significant trends. Offshore cetaceans and baleen whales also had over a third of populations with Unknown trends over three generations.

### Comparison with IUCN Data

**Figure 4:**
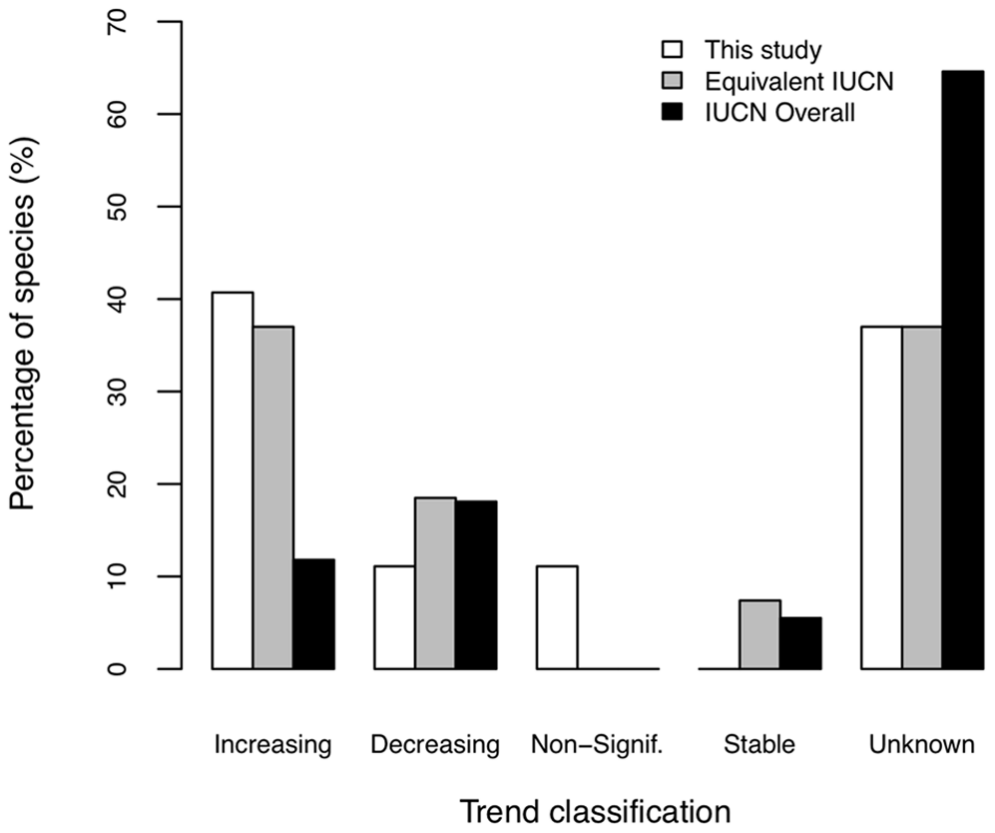
Comparison of marine mammal population trend classification results from this study with IUCN classifications. Percentage of marine mammals at the species level in abundance trend categories from this study (n = 27), the equivalent IUCN species (n = 27), and all marine mammal species assessed by IUCN (n = 127). The IUCN does not have a “Non-Significant” category as in this study, but it does have a “Stable” category not used in our results.

The IUCN lists trend classifications for 127 marine mammal species, while we had data for populations from 47 species. We had enough population-level data to allow for comparison of 27 of the 47 species to the IUCN classifications (Figure 4, Table S3). Most trend classifications were similar, with approximately 37–41% of species showing Increasing trends, 11–19% Decreasing, 7% Non-Significant or 11% Stable (these were two categories that did not match between the IUCN classifications and the ones in our study), and 37% Unknown (Figure 4). However, compared to all 127 mammals in the IUCN database, our data showed a higher proportion of Increasing populations and lower proportions of Decreasing and Unknown populations.

### Historical Declines and Recent Increases

**Figure 5:**
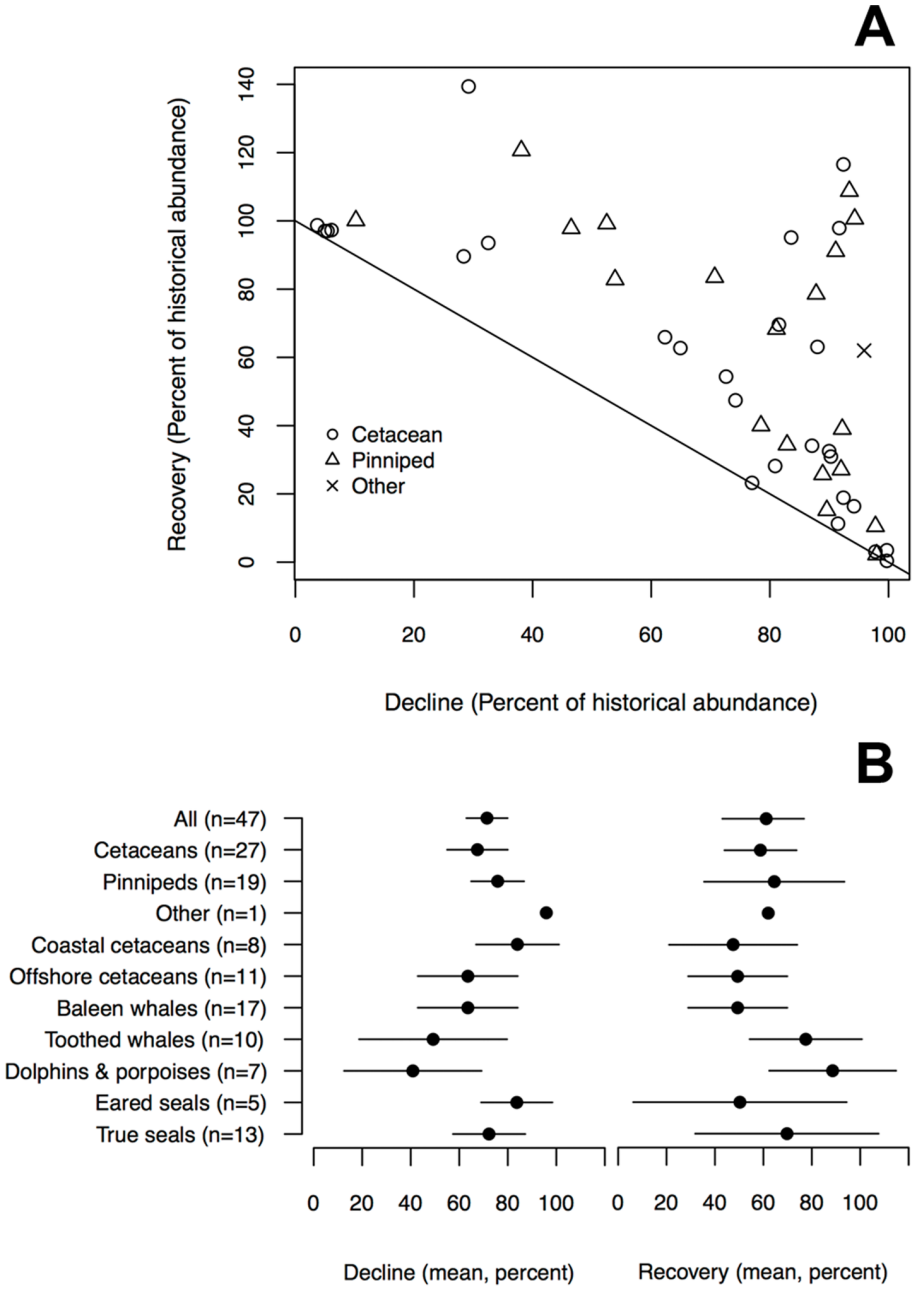
Historical decline and recent recovery relative to historical level for 47 non-nested marine mammal populations. Proportional decline and recovery in abundance for individual populations (A), and average decline and recovery across different categories of marine mammals (B). Note that in (A) that recovery must be equal to or greater than decline for a population (i.e. in the area above the diagonal line).

Among all non-duplicated populations in our database (n = 182), we had 47 non-nested populations that included a historical, minimum and recent population estimate (Figure 5, Table S4). Historical abundance declines and recent recoveries, as a proportion of the historical abundance estimate(s), ranged from virtually zero to over 100%, with some population recoveries exceeding the best available historical population estimates we could find. One should note that populations with smaller historical declines (e.g. to 90% of historic levels) could only increase over a smaller range (e.g. between 90 and >100%), while those with large historical declines (e.g. to 10% of historic levels) could increase over a much larger range (e.g. between 10 and >100%). Overall, however, there were many populations with very large historical declines >90% that have so far shown only small recent increases (clustered in the lower right corner of Figure 5A on or slightly above the diagonal line). Yet there was also considerable variation in recoveries among populations with declines >80%, and five populations with very high declines to <10% of historical levels also showed very high recoveries to >90% of historical levels. These included South African fur seals (South Africa and Namibia), harbour seals (Washington coast, Oregon), and humpback whales (North Atlantic and North Pacific).

On average, all marine mammal populations declined by 71% and have so far recovered to 61% of their historical abundance (Figure 5B). Recovery responses were generally more variable within each group than declines, and pinnipeds (true seals and eared seals) showed the most variation. Cetaceans that spend the majority of their time in coastal areas instead of offshore waters and other marine mammals (in this case, n = 1 sea otter population) showed the greatest mean declines (93% and 96% respectively), and coastal cetaceans the lowest recoveries (38%). The two groupings that had the lowest mean declines, just dolphins and porpoises (41%) and all toothed whales (49%), showed the highest mean recoveries (89% and 78% respectively).

## Discussion

The aim of our study was to perform a quantitative assessment of recovery in marine mammal populations worldwide using the best publicly available abundance data. Overall, our results suggest that more marine mammal populations are recovering than not, especially among pinnipeds, other marine mammals (e.g. sirenians, polar bears and otters) and coastal cetaceans, but a large proportion (43%) of populations have non-significant or unknown trends mainly due to high variability or data scarcity. Our study also suggests that, in general, more historically depleted populations have so far shown either low or variable recovery. These results highlight the many recovery successes while also drawing attention to those populations needing more attention in terms of monitoring, management and conservation.

### Available Marine Mammal Population Data

Notwithstanding that population level abundance data are limited in many ways for marine mammals, we successfully compiled a substantial number of time series that represented 37% of marine mammal species worldwide. These time series provided insight into data availability and quality at the population level, as well as valuable information pertaining to recoveries. Any additional data could easily be included in our database for further investigations.

Populations with better abundance time series were found to share common characteristics. They typically had either present or past commercial or cultural value, as is the case with many large whales and pinnipeds, or they were iconic or charismatic species such as killer whales (*Orcinus orca*) or common bottlenose dolphins (*Tursiops truncatus*). Moreover, species with good data were generally easier to monitor because of some combination of factors that made them accessible and visible. These factors included aspects of their behavior, such as the regular use of haul-out and breeding areas by numerous pinniped species [46], consistent coastal migration routes as for the gray whale on the west coast of North America [56], or long times spent at the water surface, as with North Atlantic right whales (*Eubalaena glacialis*) [57]. Habitat or body size characteristics may have also contributed to better time series information: abundance data collection may be easier for animals in accessible coastal areas, with smaller, well-known ranges, with large body size (e.g. great whales), or with individually identifying markings (e.g. killer whales) [3], [26], [46].

Definite gaps in our knowledge of marine mammal abundance trends still exist, namely for beaked whales, river dolphins, sirenians and Antarctic true seal populations. Beaked whales are typically pelagic deep-divers that spend very little time at the surface where there can be detected. Moreover, they have large ranges and low densities [46]. Relative lack of commercial value and recognition in the public sphere may also have contributed to the lack of monitoring and data for these species, although short (typically <10 years), sparse, recent time series [46], [47] or estimates [58] do exist for some populations. Time series data will likely improve due to interest in the susceptibility of beaked whales to acoustic disturbance, especially from seismic and naval sonar testing [46], [59]. Many river dolphins are similarly cryptic, live at low densities, or do not gather in social groups. A lack of conservation and monitoring plans with standardized population and habitat assessment techniques has limited data availability for them [23]. Although intensely studied, West Indian or Florida manatees (*Trichechus manatus*) are difficult to observe and reliable abundance estimates are challenging to obtain [60]. The lack of management has also contributed to a lack of time series data for Amazonian (*T. inunguis*) and West African manatees (*T. senegalensis*) [61], [62]. Despite intensive survey efforts in many areas, low densities and large ranges have inhibited reliable abundance estimates for dugongs (*Dugong dugon*) [63]. Many Antarctic true seal populations have relatively recent population estimates and are thought to have healthy populations, but they lack longer time series or historical estimates due to the inaccessibility of their remote breeding habitats until recently [24]. Abundance monitoring of Antarctic species may become more important for assessing and managing the effects of climate change and expanding Antarctic fisheries [64], [65], [66].

The time spans for which data were available varied between different groupings of marine mammals. Three generations of data were available for 52% of populations, while shorter time series were typically available for dolphins, porpoises, small whales, polar bears (*Ursus maritimus*), and sirenians. These data gaps are not surprising considering that many of these populations were not heavily commercially exploited, and thus abundance records likely only began recently with management and monitoring. Populations of great whales, Pacific dolphin species, northern true seals, belugas (*Delphinapterus leucas*) and narwhals (*Monodon monoceros*) made up the majority of the populations with historical abundance estimates. Historical population estimates for commercially valuable great whales typically came from either (1) catch data or product trade records, or (2) back-casting or genetic analysis techniques [6], [67], [68]. Numerous dolphin species found in Pacific U.S. waters were impacted by the tuna fishery that was established in the 1960 s [69], [70], and they are a management concern under the U.S. Marine Mammal Protection Act. Certain northern marine mammal species (northern true seals, walrus, polar bear, beluga, narwhal, and bowhead whales) are still exploited for subsistence use by aboriginal groups, and in certain cases commercially or for sport hunts. They have been subject to study and management, although survey data collection can be logistically challenging (e.g. [9], [40], [71]–[74]). Commercial pinniped hunts exist in Canada, Greenland, Namibia, Norway and Russia for several true and fur seal populations [40], [75]. With the lack of historical catch or abundance data for many populations, the development of genetic techniques [67], [68] or habitat availability analyses [76] for estimating historic population size may provide more insight into pre-exploitation estimates for some populations in coming years.

We also found geographical biases in the data, which was typically from North America, Europe (especially northern Europe), and to a lesser extent Australia, New Zealand, Japan, South America and southern Africa. This bias is generally mimicked in global marine fish abundance datasets (e.g.[77]–[79]) and the distribution of worldwide line-transect cetacean survey effort [80]. A recent study by Kaschner et al. found that the Eastern Tropical Pacific has been the most surveyed region in the world via aerial or ship-based line-transects for cetaceans since the 1980 s [80]. The geographical biases in the data also reflect the availability of financial and logistical resources for monitoring and assessment in richer nations. With the increasing interest in the value of global biodiversity, monitoring may expand to other more data-poor areas. Population abundance monitoring is important for both conservation (e.g. recovery plans) and sustainable extractive management to assess trends and reference points for conservation goals and management targets [25], [35], [77].

### Marine Mammal Population Trends

Our analysis provides a general overview of recovery trends across marine mammal populations for which data was available. Amalgamating data from numerous different sources posed challenges, but we chose analytical techniques and recovery definitions accordingly. Using a robust regression over three generations allowed us to estimate in a statistically sound manner the dominant recent abundance trends for the largest number of marine mammal populations, as scaled to life history and comparable to IUCN methods [52]. In a few cases, historical estimates were included in the 3-generation regression timespan, but the robust regression generally downweighted high leverage points (i.e. abundances that are far from the mean). The analysis of historical decline and recent recovery, however, allowed us to incorporate populations with longer time spans and historical abundance estimates, but insufficient data over three generations, into the study. Our method for identifying population trends and those populations showing signs of increase or recovery – i.e. those showing a statistically significant increase in abundance, robustly estimated using scaled data – also worked well for the challenges we faced, including non-uniform data time-spans and different life histories. Only in a few cases with short and/or highly variable time series did this method not capture what by sight appeared to be the actual trend of the data. In other analyses, depending on the goals, more specific definitions of recovery may be appropriate [25]. In addition, the sample size of other marine mammal populations in this analysis was low (n = 7) and further investigation of this category is recommended as more population data becomes available, particularly for species other than sea otters.

Overall, 42% of populations were Significantly Increasing, 10% were Significantly Decreasing, 28% showed Non-Significant Change, and 20% were deemed Unknown. The somewhat large proportion of Non-Significant and Unknown trends points to the difficulty of studying abundance trends in these animals and estimating their population size with accuracy, stressing the need for better monitoring efforts for certain groups. Improvements in increasingly used techniques such as acoustic monitoring, tagging, photo-identification and mark-recapture, as well as computerized database and analysis technologies, modeling, and data sharing among organizations, including incorporating local community knowledge [81], may improve data quality and quantity.

Despite the data gaps, those populations with sufficient data showed some interesting patterns. Half of the pinniped populations were Significantly Increasing. Their fast life history characteristics (e.g. shorter generation times) may have helped promote recovery. Management and conservation efforts, in terms of limiting direct exploitation, bycatch and trade, as well as either the isolation or protection of important haul-out or breeding habitats, likely also contributed to recoveries in some populations [24].

Coastal cetaceans were also recovering relatively well, possibly because of their early exploitation and subsequent relatively early management, conservation, and attention in the public sphere. Large, coastal cetaceans were often the first species commercially hunted in an area because of their economic value and relatively easy access (e.g. bowhead [82], North Atlantic right [82], gray [56], and humpback whales [82]). International concern over steep declines in numbers by the 1900 s for most populations led to some of the first multilateral conservation agreements, protection from international trade, domestic exploitation bans or regulations, habitat protection, and recovery planning [3], [25], [26], [83]. Numerous marine mammals have also become endearing symbols of the environmental movement [84].

Less visible or charismatic species, such as more predominantly offshore or smaller cetaceans, may have suffered in terms of population recovery for a few reasons. They were often exploited after the depletion of more easily accessible coastal species. Later onset of management or lack of directed management may have also been a contributing factor.

Some toothed cetaceans, such as sperm, pilot (*Globicephala* species) and killer whales, also have highly developed social structures that may be important to survival. As a result they may be more heavily impacted by the effects of selected removal and small population size [85], [86]. Critical factors which may determine population recovery have been investigated, and appear to support the assertion that primarily coastal marine mammals had a higher probability of recovering than offshore populations, as did earlier maturing populations and pinnipeds (Anna M. Magera, MSc thesis, Dalhousie University, 2011).

### Comparison with IUCN Data

In order to verify our population-level trends, we compared our results with IUCN assessments for those species for which we had the majority of the species’ abundance data at a population level. This included 27 species, approximately 21% of all marine mammal species worldwide. For these, we did have good agreement with the equivalent IUCN trend determination for the same species, which strengthens confidence in our results. However, we clearly had more Increasing, and fewer Decreasing and Unknown species (populations) compared to the global IUCN assessment of all 127 marine mammals (Figure 4). This is primarily attributable to the absence of data that met our criteria for many rare or difficult to monitor species, which resulted in them being excluded from the study (see Methods), combined with the large percentage (58%) of species classified as data-deficient by the IUCN.

### Historical Declines and Recent Increases

Many populations with available time series did not have pre-exploitation or K estimates of historical population size. However, we were able to obtain estimates of historical population size and thus compare the magnitude of historical declines and recent recoveries for 47 of the non-nested populations. As shown for marine fish populations [39], [87], smaller historical population declines were generally associated with more successful recent recoveries. In turn, populations with very large historical declines (>90%) in fish [39], [87] and many marine mammals (this study) have shown low magnitudes of recovery so far, with some notable exceptions. The large proportion of populations with >60% to >90% declines in this study highlights the substantial historical declines in many marine mammal populations [2].

We also found differences in declines and recoveries between different types of marine mammals, with the smaller, typically less commercially valuable toothed whales, and especially porpoises and dolphins showing the smallest historical declines and greatest recent recoveries. In turn, easily accessible coastal cetaceans and other marine mammals showed the largest historical declines. Sethi et al. (2010) note that in commercial fisheries development is typically driven by which taxa will produce the most profit at the minimal cost [88]. Similar explanations certainly exist for the history of whaling and sealing [7], [13]. Recovery among marine mammal populations showed quite high variability. However, on average eared seals, baleen whales, as well as offshore and coastal cetaceans seemed to show the smallest recoveries, likely because of heavy historical exploitation (eared seals, baleen and coastal cetaceans) or lack of management (offshore cetaceans) [7], [13], [24].

Other studies have quantified historical declines and, in part, recoveries in marine mammals. Christensen (2006) estimated historical baselines for exploited cetaceans and pinnipeds and found a cumulative decline of 22% (range: 0–62%) in numbers between 1800 and 2001. The largest declines were in the great whales (64%, range: 40–79%) during periods of increased catches [11], which is comparable to the declines we estimated for the baleen whales. Christensen’s overall marine mammal declines, however, presented a smaller decrease in terms of overall numbers than calculated in our study, likely because Christensen [11] relied mainly on the use of catch data-driven models and limited her assessment of cumulative marine mammal declines to 1800–2001. By this time some populations (e.g. North Atlantic right whales) had already been substantially depleted. A second recent study of historical baselines for large marine animal populations (not limited to a specific period) estimated a historical decline of approximately 96% for pinnipeds, otters and sirenians, and a recovery to approximately 25% of historical abundance [2]. Whales declined by approximately 82% and recovered to approximately 32% of historical levels. The pinniped, sea otter and sirenian category was not directly comparable to any of our categories, but for pinnipeds, our study presented smaller declines and larger recoveries. This may have been because the study included populations that did not exhibit any recovery, including extirpated populations, while our examination of n = 47 populations did not. The whale category only included great whale populations (mainly baleen) and was comparable to our baleen whale decline and recovery results [2].

Although there was relatively high variability in population recoveries after large historical declines in our study, many populations showed minimal population recovery following very high declines despite many decades of protection (e.g. right and bowhead whales since the 1930 s), thus possibly indicating Allee effects [89]. Only five populations out of 18 that underwent very large declines to <10% of the historical population abundance also showed very high (>90%) recoveries. This reflected a similar finding in certain fast-growing clupeid populations, which recovered to levels that were not observed in any other types of fish after declines of similar magnitude [39], [89]. Possible explanations include a faster life history and relatively early age at maturity (4–5.5 years) that allowed for more rapid population increase as well as a longer time since exploitation was halted and better protection [26], [90]–[96]. Other possible explanations might be that historical population estimates underestimated true pre-commercial exploitation population size, or ecosystem conditions changed in these areas to favor substantial population growth and larger abundances.

## Conclusion

Despite sparse data for many species and regions, lack of historical abundance estimates and often large error associated with available data, we were able to assess the recovery status for 182 marine mammal populations worldwide. Overall, 42% of populations for which we have good abundance time series are recovering from former depletions, especially pinnipeds, coastal cetaceans and other marine mammals (i.e. polar bears, otters and sirenians). Offshore cetaceans, all toothed whales, and just dolphins and porpoises showed relatively few recovering populations. On a species level, our results were comparable to assessments performed by IUCN, suggesting that our robust weighted regression over three generations is a useful and appropriate method for estimating general population trends. However, compared to all marine mammal species assessed by IUCN, our data over-represented recovering populations due to a lack of data for data-poor populations, most notably the sirenians, river dolphins and beaked whales. We also found that populations with smaller historical population declines were more likely to show stronger population recoveries in more recent times, while those that have been extensively depleted showed more variability in their recovery success, but tended to have smaller recoveries. This synthesis and compiled database are useful tools for other researchers interested in marine mammal population trends as well as decision makers to guide management and conservation efforts towards non-recovering populations. Moreover, our results stress the need for enhanced and increasingly innovative monitoring of offshore and cryptic marine mammals and those in low-latitude and developing nations that have not been extensively studied. Available and reliable abundance data are critical for better management and conservation, and will help to produce more complete and accurate trend estimates and recovery assessments in the future.

## Supporting Information

Figure S1 **Marine mammal population abundances over time and trends over three generations for robust log-linear (A) and robust linear (B) regressions.** Species and population areas are described in the upper left hand corner of each plot (n = 198 populations with duplicates, n = 182 populations without duplicate regular and pup count data). Population robust regression trend classification (long-linear (A) or linear (B)) is indicated in the upper right hand corner: I = Significantly Increasing, D = Significantly Decreasing, NS = Non-Significant Change, NA = Unknown. Solid lines = robust regression weighted by Abundance Confidence ID (ACID). Solid points = abundance data with quantitative error information (95% confidence interval bars). Empty points = abundance data with no stated quantitative error information. Black points = regular data that was collected from the entire population. Grey points = indicate pup count data.
(PDF)

Table S1 **Abundance data source information for study populations.** For each population, denoted by a numeric area code (Population Area ID) and area description, abundance data sources are listed along with the data collection and/or additional analysis methods used to obtain the population abundance estimates. Abundance Confidence ID (ACID) provides an uncertainty rank (1 to 6, 1 = lowest, 6 = highest). CPUE = catch per unit effort.
(DOC)

Table S2 **Overview of population time series: area descriptions, generation times, time spans, and data types.** All time references are in years. Time series refer to population abundance time series. List of abbreviations used in table: Species Type = general species taxon – cetacean, pinniped or other (sirenian, otter or polar bear). Regular Data Type = non-pup count data. Pup Count Data Type = only pup count data. N, S, E, W = North, South, East, West. Include Small/Large Area = was the population included if the smallest or largest non-nested populations were considered for the species? (1 = yes, 0 = no). Dominant Habitat = dominant habitat type. Sub-type = sub-type of marine mammal within species type. Dolphin or Porpoise? = whether the population is a type of a dolphin or porpoise.
(XLS)

Table S3 **Abundance trends for 127 marine mammal species listed by the IUCN (2008).** Species Type = general species taxon – cetacean, pinniped or other (sirenian, otter or polar bear). Data? = whether or not population level data for the species is included in this study. IUCN Trend = species trend listed on the IUCN Red List of Threatened Species (2008). IUCN typically describes abundance trends on a species level, while this study examines population level trends. Note, IUCN does not have a “Non-Significant” category as in this study, but does have a “Stable” category not used in our results.
(XLS)

Table S4 **Historical** **population declines and recent recoveries with respect to historical level.** For populations (n = 47) with historical, minimum and recent abundance estimates. Decline and recovery percentages are mean values for populations where more than one historical estimate is present. In some cases, recent abundance values are higher than historical population estimates, leading to recovery values of greater than 100%.
(XLS)

Table S5 **Results from scaled robust log-linear and robust linear regressions for marine mammal populations (n = 198).** The regressions were weighted by Abundance Confidence ID (ACID) over three generation times. Duplicate pup count/regular populations included in blue text. These will not be used in further analyses, resulting in 182 remaining populations (out of 198). Species Type = general species taxon (cetacean, pinniped or other (sirenian, otter or polar bear)). N, S, E, W = North, South, East, West. Include Small/Large Area = was the population included if the smallest or largest non-nested populations were considered for the species. Regression & Data Type  = *log* lmRob or lmRob and Regular (i.e. non-pup count data) or Pup Count (i.e. only pup count data). Coefficient or slope = the scaled rate of population growth (i.e. change in abundance over time). SE = standard error. CI95 = 95% confidence interval. tval = t-value. pval = p-value. Increasing_Sig  =  significantly increasing abundance trend. Increasing_NS = non-significant increasing abundance trend. Decreasing_Sig = significantly decreasing abundance trend. Decreasing_NS = non-significant decreasing abundance trend. NA indicates a lack of sufficient data over the three-generation time period necessary to perform a robust regression analysis. Dominant Habitat = dominant habitat type. Sub-type = sub-type of marine mammal within species type. Dolphin or Porpoise? = whether the population is a type of a dolphin or porpoise.
(XLS)

Text S1 **Abundance confidence ID (ACID) system: explanation and verification.**
(DOC)

Text S2 **R Statistical Software code for analyzing marine mammal population abundance trends.**
(DOCX)
