# Supplementary material for: Recovery Trends in Marine Mammal Populations
Source: PLoS One. 2013 Oct 30;8(10):e77908. doi: 10.1371/journal.pone.0077908 (PMC3813518; doi:10.1371/journal.pone.0077908)
Supplement: Figure S1 — Marine mammal population abundances over time and trends over three generations for robust log-linear (A) and robust linear (B) regressions. Species and population areas are described in the upper left hand corner of each plot (n = 198 populations with duplicates, n = 182 populations without duplicate regular and pup count data). Population robust regression trend classification (long-linear (A) or linear (B)) is indicated in the upper right hand corner: I = Significantly Increasing, D = Significantly Decreasing, NS = Non-Significant Change, NA = Unknown. Solid lines = robust regression weighted by Abundance Confidence ID (ACID). Solid points = abundance data with quantitative error information (95% confidence interval bars). Empty points = abundance data with no stated quantitative error information. Black points = regular data that was collected from the entire population. Grey points = indicate pup count data. (PDF) [file pone.0077908.s001.pdf]

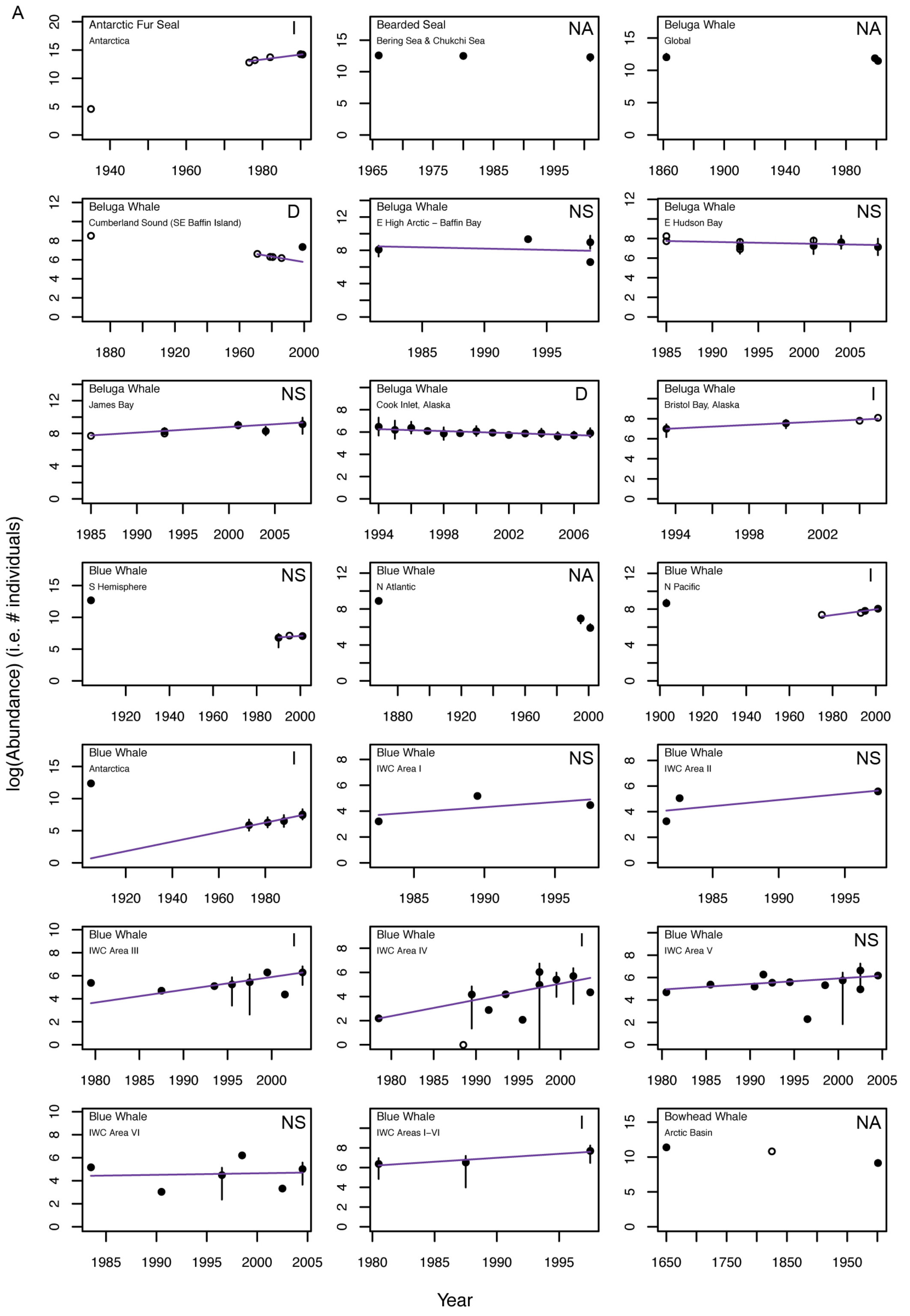

A
Antarctic Fur Seal
Antarctica
I
Bearded Seal
Bering Sea & Chukchi Sea
NA
Beluga Whale
Global
NA
Beluga Whale
Cumberland Sound (SE Baffin Island)
D
Beluga Whale
E High Arctic – Baffin Bay
NS
Beluga Whale
E Hudson Bay
NS
Beluga Whale
James Bay
NS
Beluga Whale
Cook Inlet, Alaska
D
Beluga Whale
Bristol Bay, Alaska
I
Blue Whale
S Hemisphere
NS
Blue Whale
N Atlantic
NA
Blue Whale
N Pacific
I
Blue Whale
Antarctica
I
Blue Whale
IWC Area I
NS
Blue Whale
IWC Area II
NS
Blue Whale
IWC Area III
I
Blue Whale
IWC Area IV
I
Blue Whale
IWC Area V
NS
Blue Whale
IWC Area VI
NS
Blue Whale
IWC Areas I–VI
I
Bowhead Whale
Arctic Basin
NA
log(Abundance) (i.e. # individuals)
Year

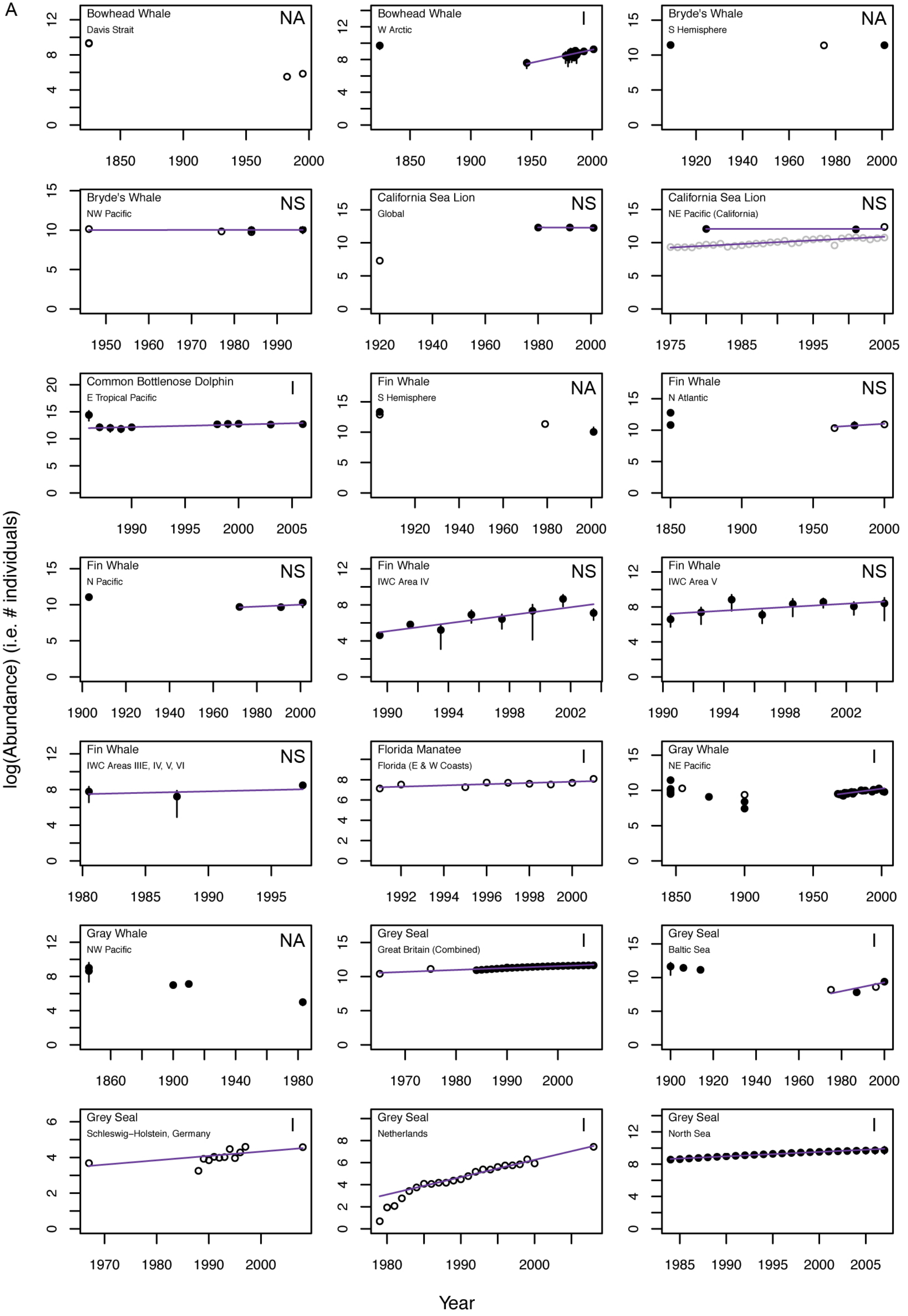
A
log(Abundance) (i.e. # individuals)
Year
Bowhead Whale
Davis Strait
NA
Bowhead Whale
W Arctic
I
Bryde's Whale
S Hemisphere
NA
Bryde's Whale
NW Pacific
NS
California Sea Lion
Global
NS
California Sea Lion
NE Pacific (California)
NS
Common Bottlenose Dolphin
E Tropical Pacific
I
Fin Whale
S Hemisphere
NA
Fin Whale
N Atlantic
NS
Fin Whale
N Pacific
NS
Fin Whale
IWC Area IV
NS
Fin Whale
IWC Area V
NS
Fin Whale
IWC Areas IIIE, IV, V, VI
NS
Florida Manatee
Florida (E & W Coasts)
I
Gray Whale
NE Pacific
I
Gray Whale
NW Pacific
NA
Grey Seal
Great Britain (Combined)
I
Grey Seal
Baltic Sea
I
Grey Seal
Schleswig–Holstein, Germany
I
Grey Seal
Netherlands
I
Grey Seal
North Sea
I

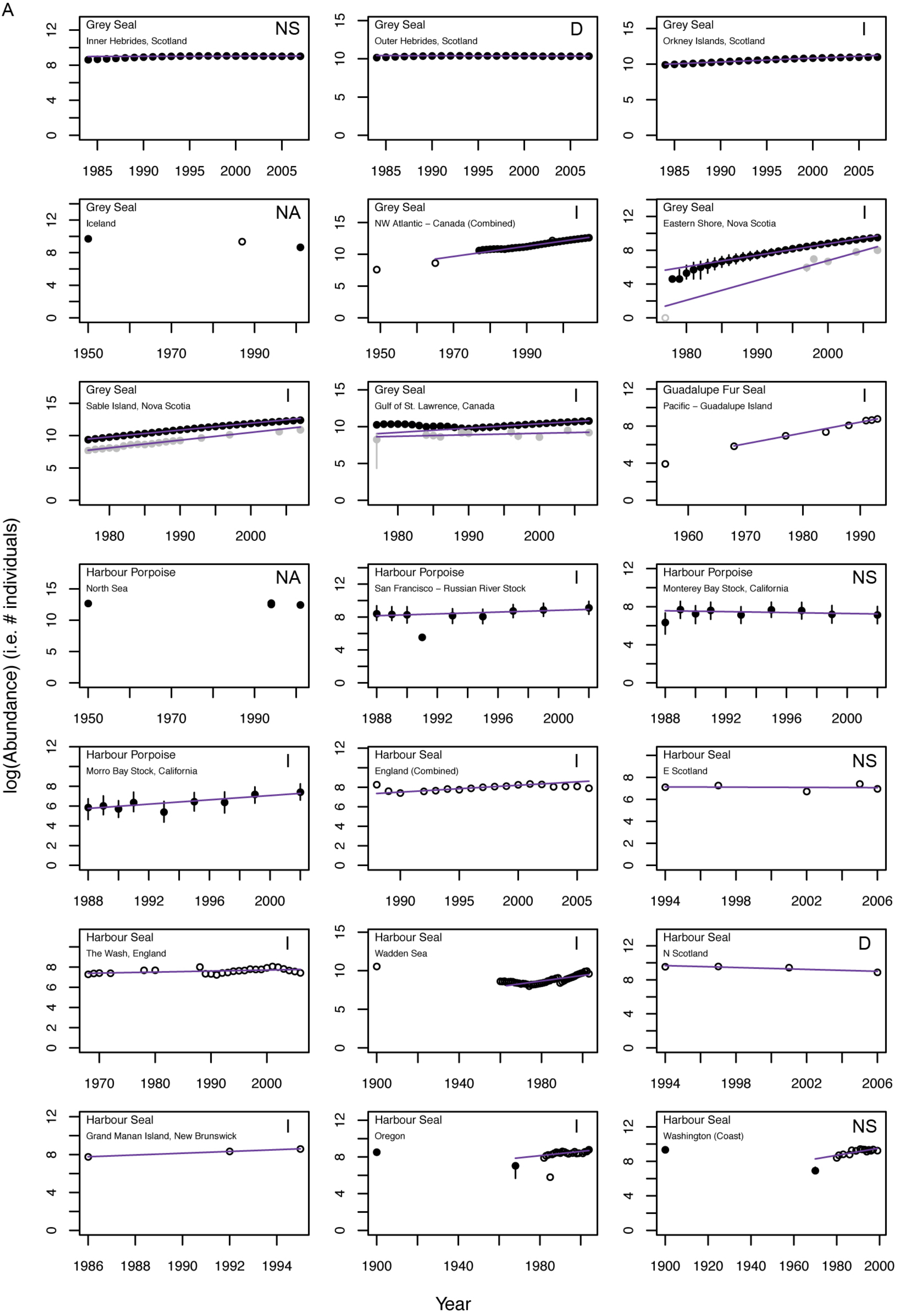
A
Grey Seal
Inner Hebrides, Scotland
NS
Grey Seal
Outer Hebrides, Scotland
D
Grey Seal
Orkney Islands, Scotland
I
Grey Seal
Iceland
NA
Grey Seal
NW Atlantic – Canada (Combined)
I
Grey Seal
Eastern Shore, Nova Scotia
I
Grey Seal
Sable Island, Nova Scotia
I
Grey Seal
Gulf of St. Lawrence, Canada
I
Guadalupe Fur Seal
Pacific – Guadalupe Island
I
Harbour Porpoise
North Sea
NA
Harbour Porpoise
San Francisco – Russian River Stock
I
Harbour Porpoise
Monterey Bay Stock, California
NS
Harbour Porpoise
Morro Bay Stock, California
I
Harbour Seal
England (Combined)
I
Harbour Seal
E Scotland
NS
Harbour Seal
The Wash, England
I
Harbour Seal
Wadden Sea
I
Harbour Seal
N Scotland
D
Harbour Seal
Grand Manan Island, New Brunswick
I
Harbour Seal
Oregon
I
Harbour Seal
Washington (Coast)
NS
log(Abundance) (i.e. # individuals)
Year

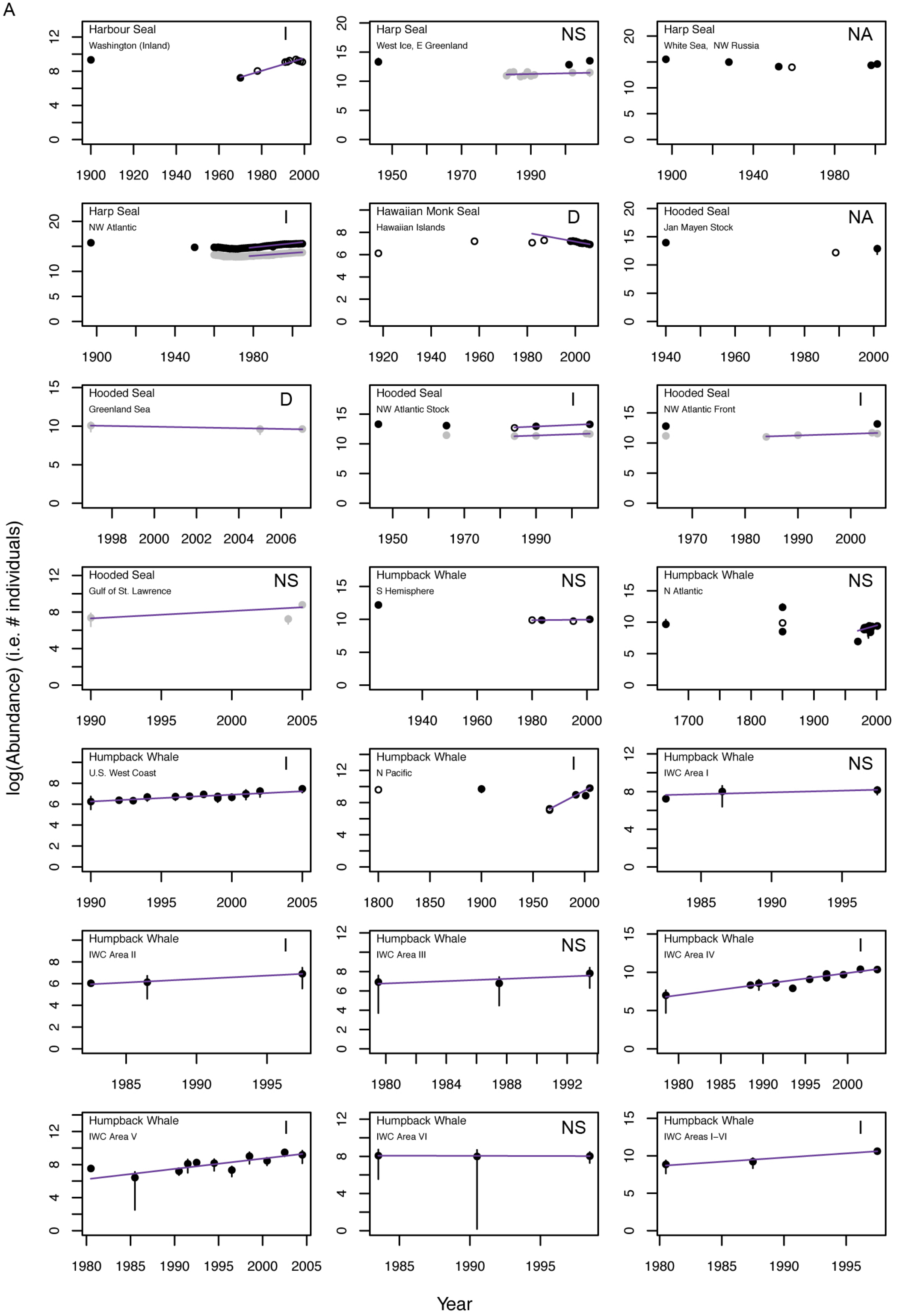
A
Harbour Seal
Washington (Inland)
I
Harp Seal
West Ice, E Greenland
NS
Harp Seal
White Sea, NW Russia
NA
Harp Seal
NW Atlantic
I
Hawaiian Monk Seal
Hawaiian Islands
D
Hooded Seal
Jan Mayen Stock
NA
Hooded Seal
Greenland Sea
D
Hooded Seal
NW Atlantic Stock
I
Hooded Seal
NW Atlantic Front
I
Hooded Seal
Gulf of St. Lawrence
NS
Humpback Whale
S Hemisphere
NS
Humpback Whale
N Atlantic
NS
Humpback Whale
U.S. West Coast
I
Humpback Whale
N Pacific
I
Humpback Whale
IWC Area I
NS
Humpback Whale
IWC Area II
I
Humpback Whale
IWC Area III
NS
Humpback Whale
IWC Area IV
I
Humpback Whale
IWC Area V
I
Humpback Whale
IWC Area VI
NS
Humpback Whale
IWC Areas I–VI
I
log(Abundance) (i.e. # individuals)
Year

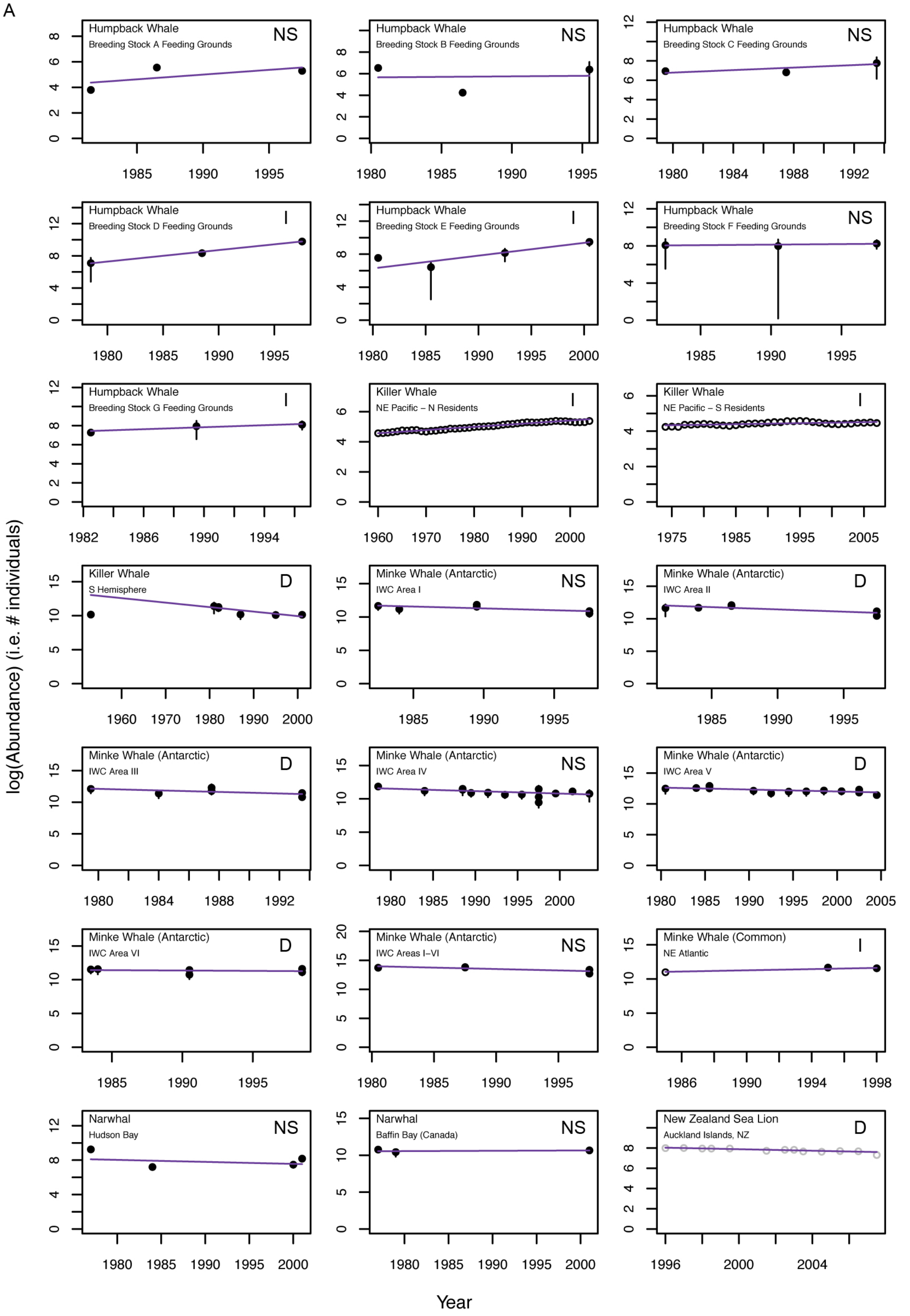
A
Humpback Whale
Breeding Stock A Feeding Grounds
NS
Humpback Whale
Breeding Stock B Feeding Grounds
NS
Humpback Whale
Breeding Stock C Feeding Grounds
NS
Humpback Whale
Breeding Stock D Feeding Grounds
I
Humpback Whale
Breeding Stock E Feeding Grounds
I
Humpback Whale
Breeding Stock F Feeding Grounds
NS
Humpback Whale
Breeding Stock G Feeding Grounds
I
Killer Whale
NE Pacific – N Residents
I
Killer Whale
NE Pacific – S Residents
I
Killer Whale
S Hemisphere
D
Minke Whale (Antarctic)
IWC Area I
NS
Minke Whale (Antarctic)
IWC Area II
D
Minke Whale (Antarctic)
IWC Area III
D
Minke Whale (Antarctic)
IWC Area IV
NS
Minke Whale (Antarctic)
IWC Area V
D
Minke Whale (Antarctic)
IWC Area VI
D
Minke Whale (Antarctic)
IWC Areas I–VI
NS
Minke Whale (Common)
NE Atlantic
I
Narwhal
Hudson Bay
NS
Narwhal
Baffin Bay (Canada)
NS
New Zealand Sea Lion
Auckland Islands, NZ
D
log(Abundance) (i.e. # individuals)
Year

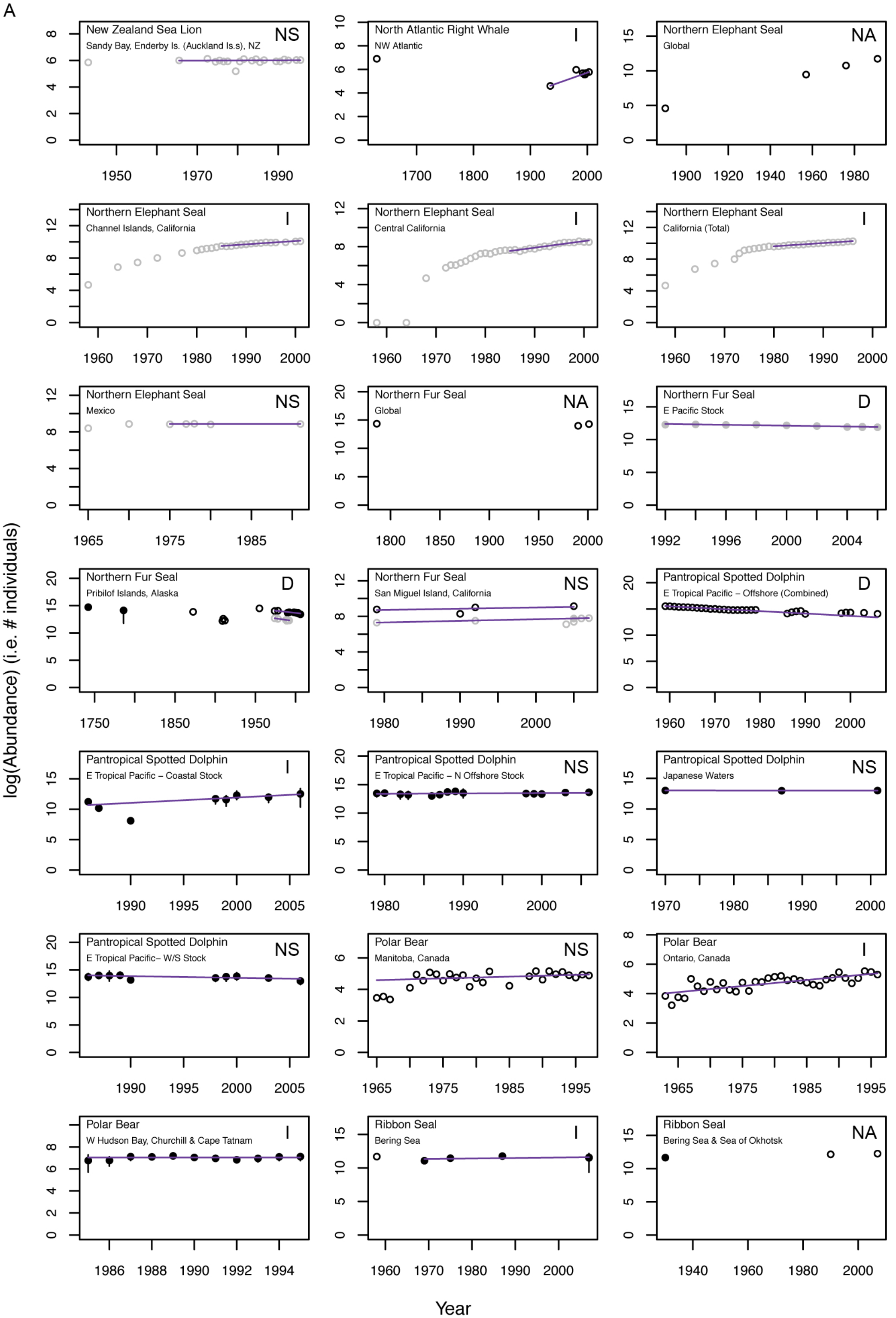

A
log(Abundance) (i.e. # individuals)
Year
New Zealand Sea Lion
Sandy Bay, Enderby Is. (Auckland Is.s), NZ
NS
North Atlantic Right Whale
NW Atlantic
I
Northern Elephant Seal
Global
NA
Northern Elephant Seal
Channel Islands, California
I
Northern Elephant Seal
Central California
I
Northern Elephant Seal
California (Total)
I
Northern Elephant Seal
Mexico
NS
Northern Fur Seal
Global
NA
Northern Fur Seal
E Pacific Stock
D
Northern Fur Seal
Pribilof Islands, Alaska
D
Northern Fur Seal
San Miguel Island, California
NS
Pantropical Spotted Dolphin
E Tropical Pacific – Offshore (Combined)
D
Pantropical Spotted Dolphin
E Tropical Pacific – Coastal Stock
I
Pantropical Spotted Dolphin
E Tropical Pacific – N Offshore Stock
NS
Pantropical Spotted Dolphin
Japanese Waters
NS
Pantropical Spotted Dolphin
E Tropical Pacific– W/S Stock
NS
Polar Bear
Manitoba, Canada
NS
Polar Bear
Ontario, Canada
I
Polar Bear
W Hudson Bay, Churchill & Cape Tatnam
I
Ribbon Seal
Bering Sea
I
Ribbon Seal
Bering Sea & Sea of Okhotsk
NA

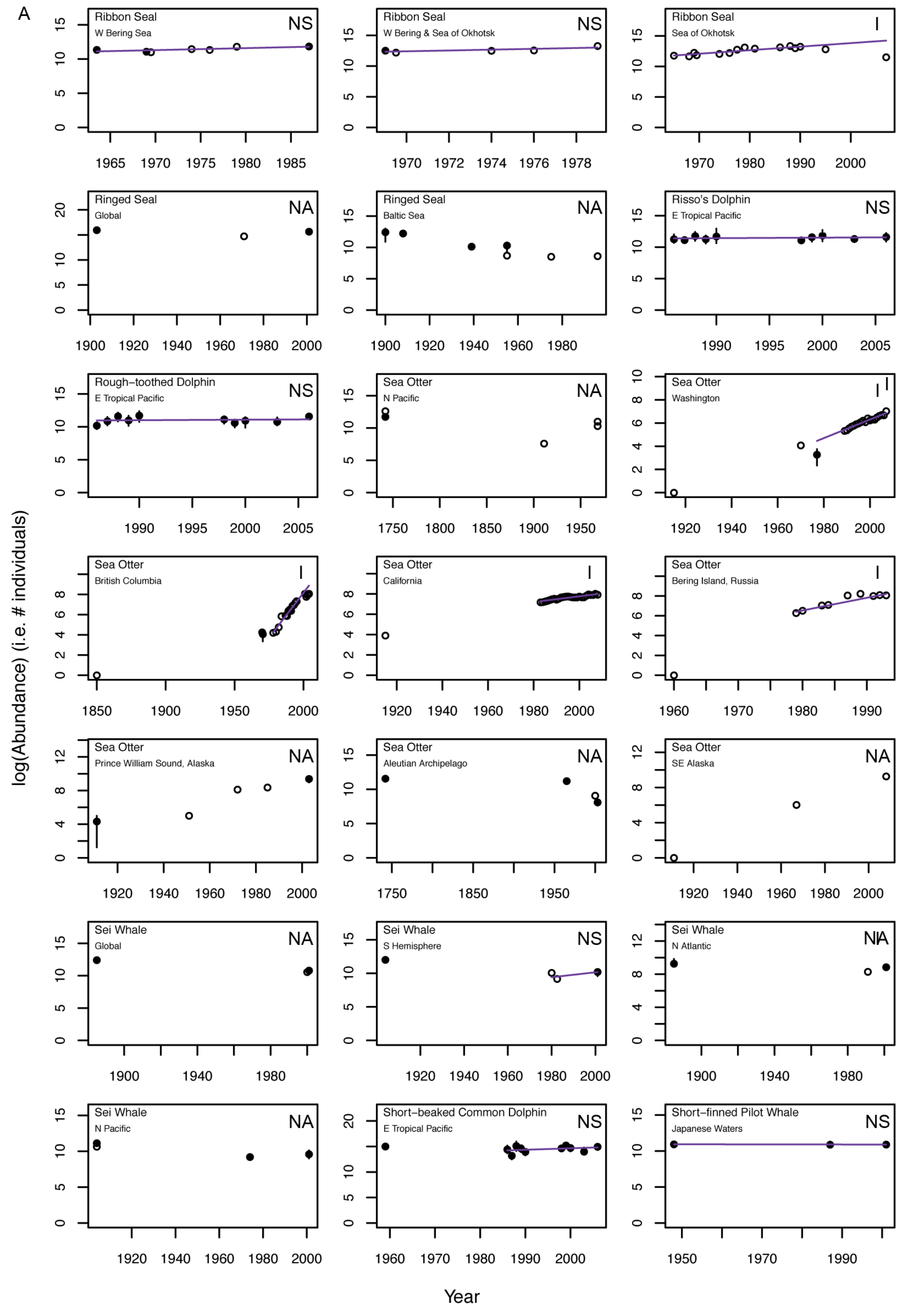
A
Ribbon Seal
W Bering Sea
NS
Ribbon Seal
W Bering & Sea of Okhotsk
NS
Ribbon Seal
Sea of Okhotsk
I
Ringed Seal
Global
NA
Ringed Seal
Baltic Sea
NA
Risso's Dolphin
E Tropical Pacific
NS
Rough–toothed Dolphin
E Tropical Pacific
NS
Sea Otter
N Pacific
NA
Sea Otter
Washington
I
Sea Otter
British Columbia
I
Sea Otter
California
I
Sea Otter
Bering Island, Russia
I
Sea Otter
Prince William Sound, Alaska
NA
Sea Otter
Aleutian Archipelago
NA
Sea Otter
SE Alaska
NA
Sei Whale
Global
NA
Sei Whale
S Hemisphere
NS
Sei Whale
N Atlantic
NA
Sei Whale
N Pacific
NA
Short–beaked Common Dolphin
E Tropical Pacific
NS
Short–finned Pilot Whale
Japanese Waters
NS
log(Abundance) (i.e. # individuals)
Year

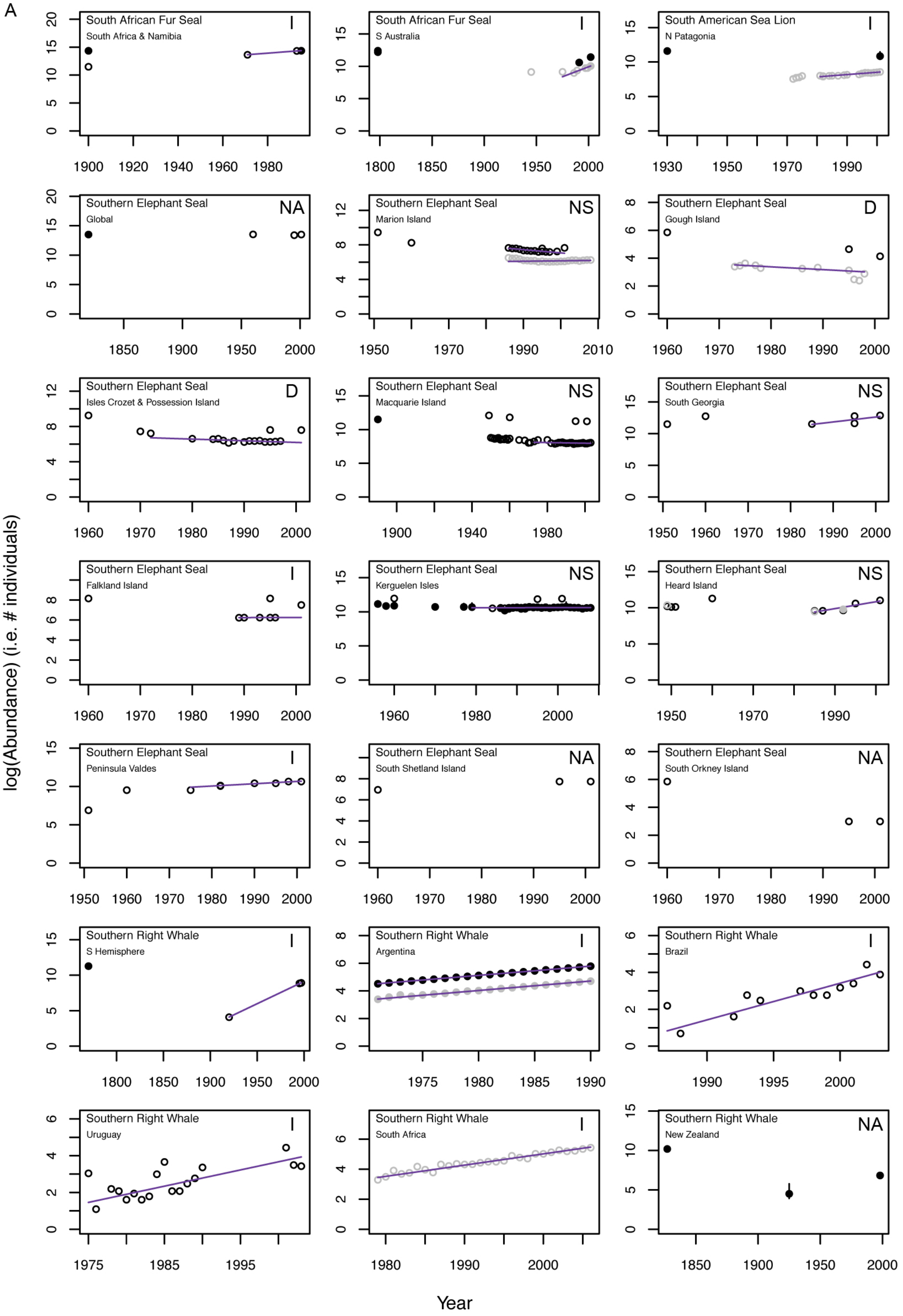
A
South African Fur Seal
South Africa & Namibia
I
South African Fur Seal
S Australia
I
South American Sea Lion
N Patagonia
I
Southern Elephant Seal
Global
NA
Southern Elephant Seal
Marion Island
NS
Southern Elephant Seal
Gough Island
D
Southern Elephant Seal
Isles Crozet & Possession Island
D
Southern Elephant Seal
Macquarie Island
NS
Southern Elephant Seal
South Georgia
NS
Southern Elephant Seal
Falkland Island
I
Southern Elephant Seal
Kerguelen Isles
NS
Southern Elephant Seal
Heard Island
NS
Southern Elephant Seal
Peninsula Valdes
I
Southern Elephant Seal
South Shetland Island
NA
Southern Elephant Seal
South Orkney Island
NA
Southern Right Whale
S Hemisphere
I
Southern Right Whale
Argentina
I
Southern Right Whale
Brazil
I
Southern Right Whale
Uruguay
I
Southern Right Whale
South Africa
I
Southern Right Whale
New Zealand
NA
log(Abundance) (i.e. # individuals)
Year

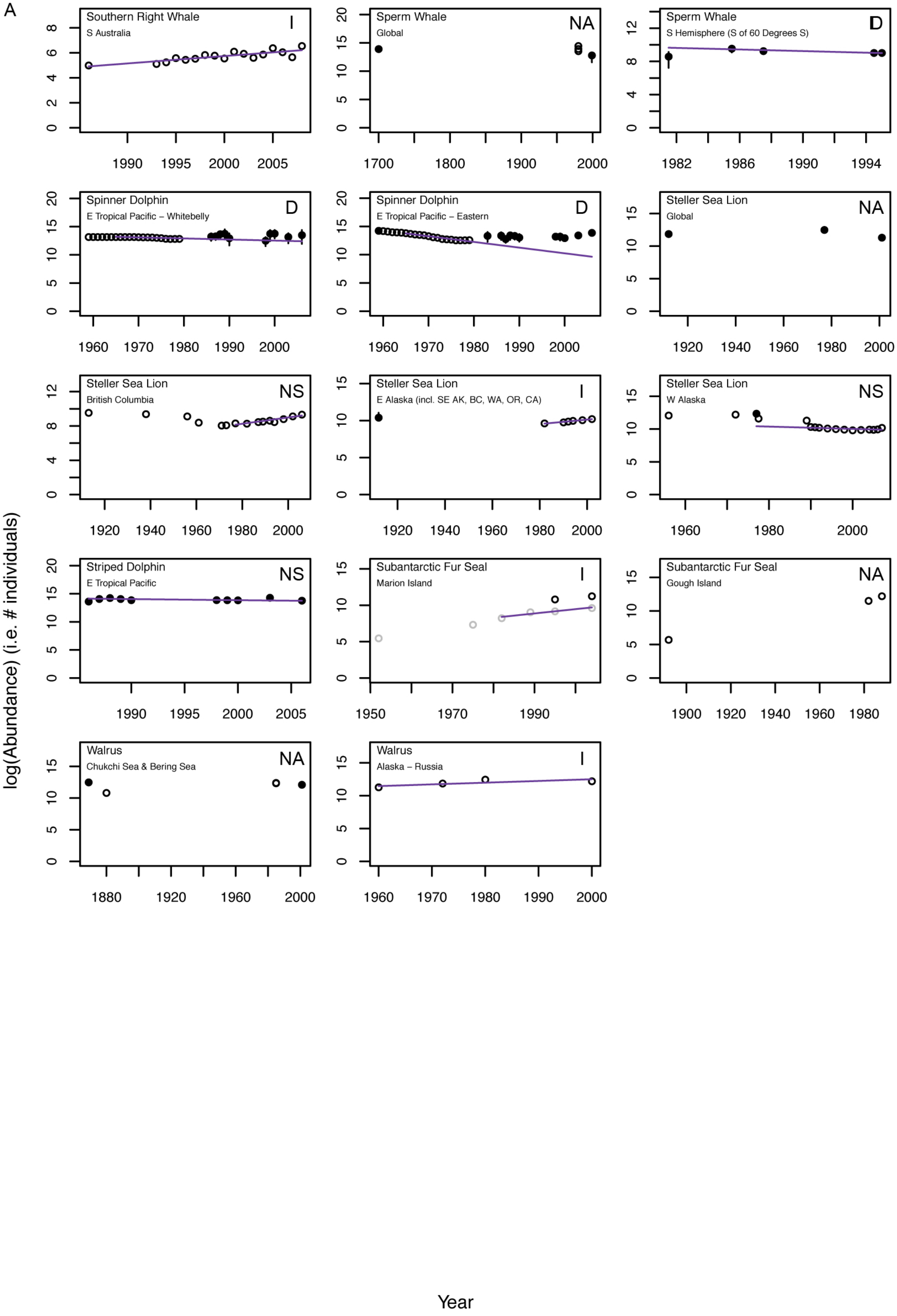

A
Southern Right Whale
S Australia
I
Sperm Whale
Global
NA
Sperm Whale
S Hemisphere (S of 60 Degrees S)
D
Spinner Dolphin
E Tropical Pacific – Whitebelly
D
Spinner Dolphin
E Tropical Pacific – Eastern
D
Steller Sea Lion
Global
NA
Steller Sea Lion
British Columbia
NS
Steller Sea Lion
E Alaska (incl. SE AK, BC, WA, OR, CA)
I
Steller Sea Lion
W Alaska
NS
Striped Dolphin
E Tropical Pacific
NS
Subantarctic Fur Seal
Marion Island
I
Subantarctic Fur Seal
Gough Island
NA
Walrus
Chukchi Sea & Bering Sea
NA
Walrus
Alaska – Russia
I
log(Abundance) (i.e. # individuals)
Year

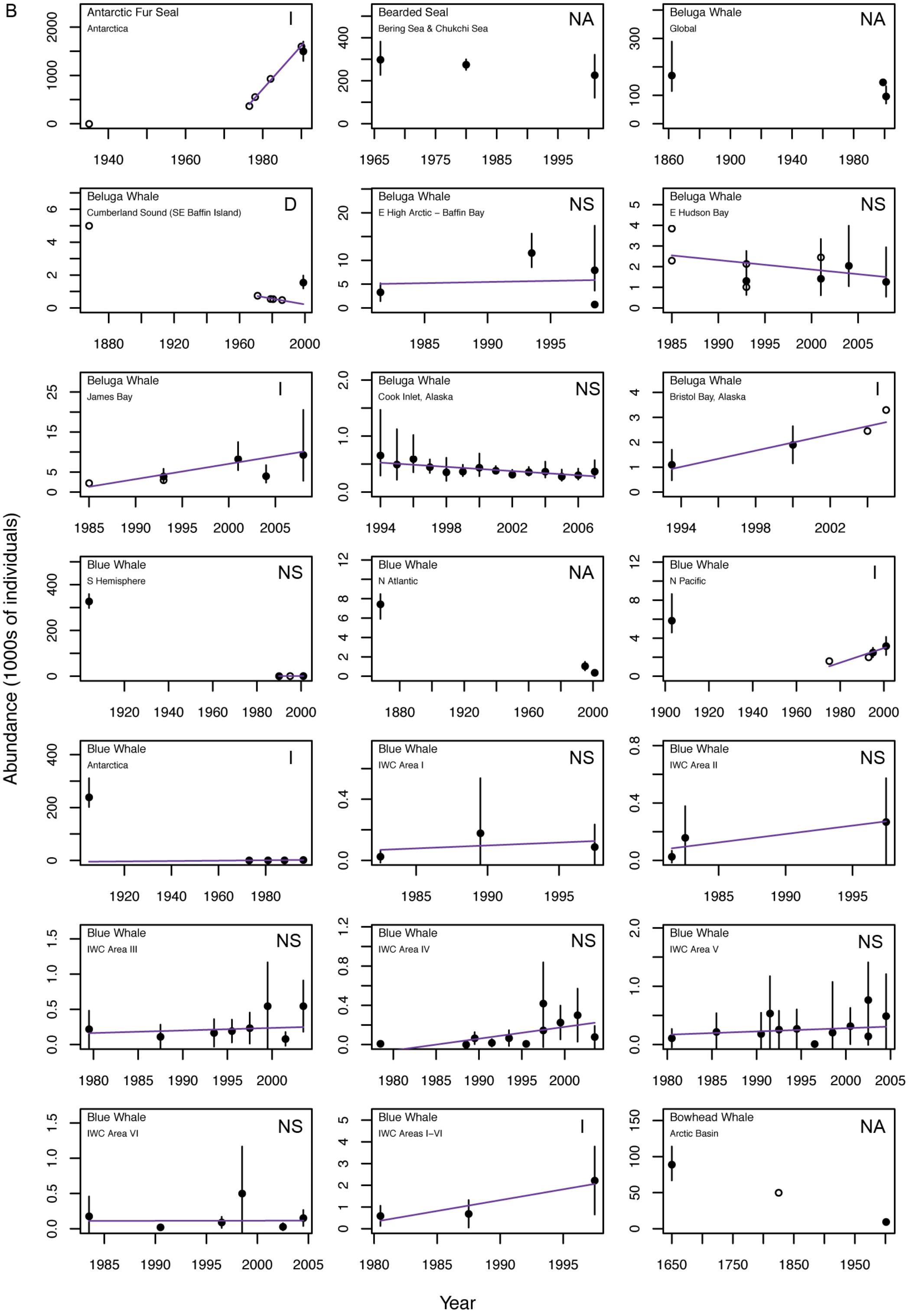
B
Abundance (1000s of individuals)
Year
Antarctic Fur Seal
Antarctica
I
Bearded Seal
Bering Sea & Chukchi Sea
NA
Beluga Whale
Global
NA
Beluga Whale
Cumberland Sound (SE Baffin Island)
D
Beluga Whale
E High Arctic – Baffin Bay
NS
Beluga Whale
E Hudson Bay
NS
Beluga Whale
James Bay
I
Beluga Whale
Cook Inlet, Alaska
NS
Beluga Whale
Bristol Bay, Alaska
I
Blue Whale
S Hemisphere
NS
Blue Whale
N Atlantic
NA
Blue Whale
N Pacific
I
Blue Whale
Antarctica
I
Blue Whale
IWC Area I
NS
Blue Whale
IWC Area II
NS
Blue Whale
IWC Area III
NS
Blue Whale
IWC Area IV
NS
Blue Whale
IWC Area V
NS
Blue Whale
IWC Area VI
NS
Blue Whale
IWC Areas I–VI
I
Bowhead Whale
Arctic Basin
NA

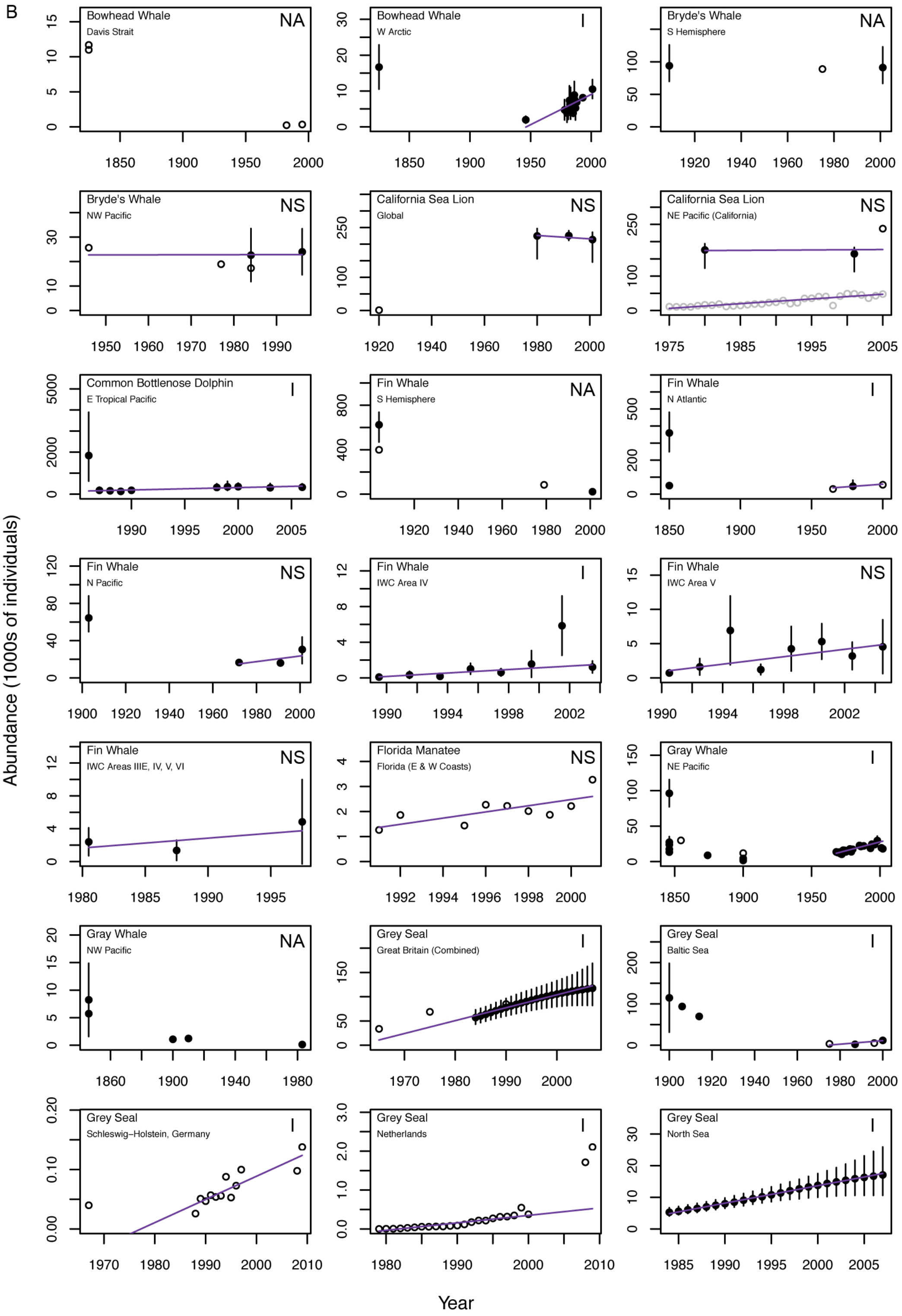

B
Abundance (1000s of individuals)
Year
Bowhead Whale
Davis Strait
NA
Bowhead Whale
W Arctic
I
Bryde's Whale
S Hemisphere
NA
Bryde's Whale
NW Pacific
NS
California Sea Lion
Global
NS
California Sea Lion
NE Pacific (California)
NS
Common Bottlenose Dolphin
E Tropical Pacific
I
Fin Whale
S Hemisphere
NA
Fin Whale
N Atlantic
I
Fin Whale
N Pacific
NS
Fin Whale
IWC Area IV
I
Fin Whale
IWC Area V
NS
Fin Whale
IWC Areas IIIE, IV, V, VI
NS
Florida Manatee
Florida (E & W Coasts)
NS
Gray Whale
NE Pacific
I
Gray Whale
NW Pacific
NA
Grey Seal
Great Britain (Combined)
I
Grey Seal
Baltic Sea
I
Grey Seal
Schleswig–Holstein, Germany
I
Grey Seal
Netherlands
I
Grey Seal
North Sea
I

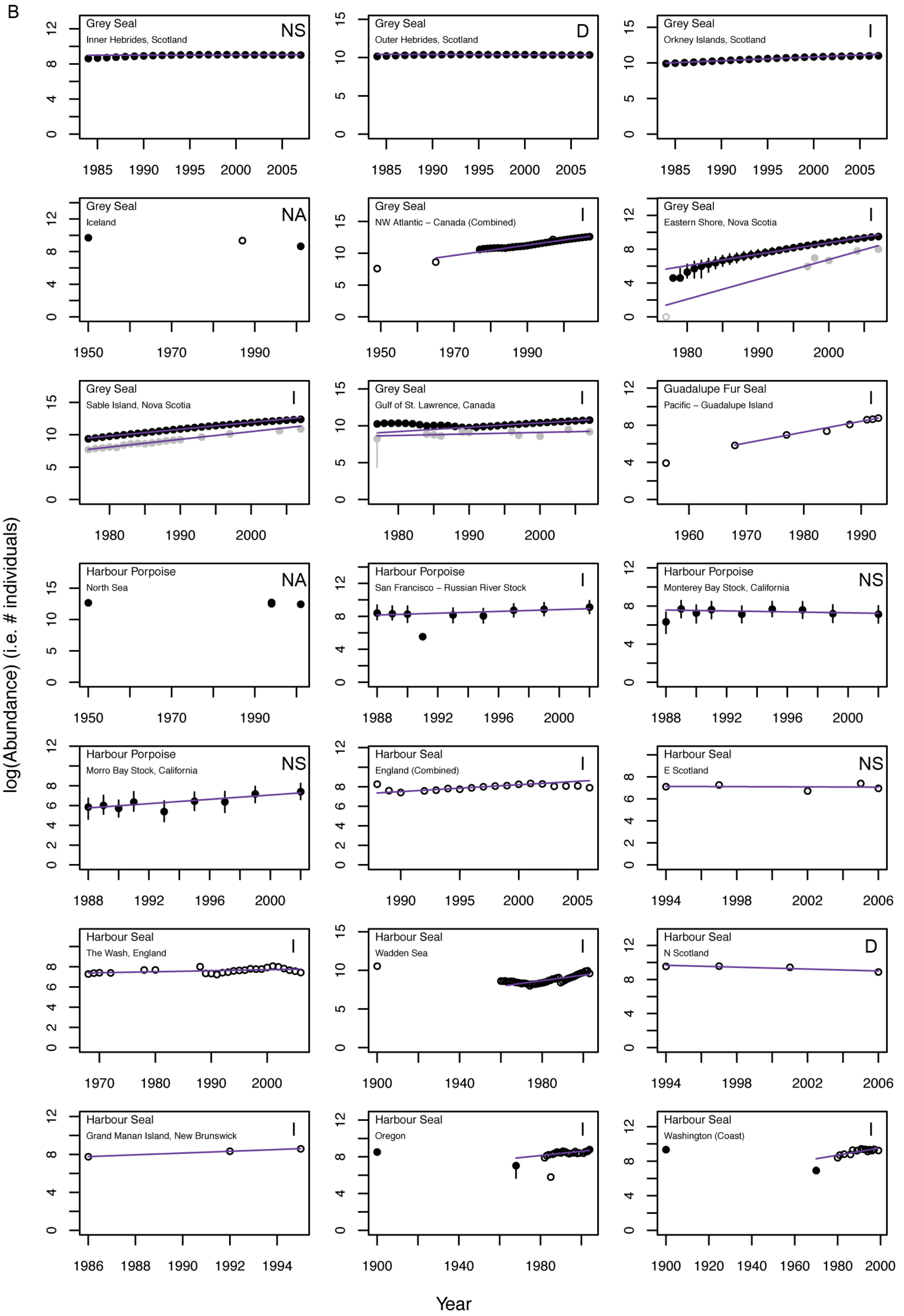
B
log(Abundance) (i.e. # individuals)
Year
Grey Seal
Inner Hebrides, Scotland
NS
Grey Seal
Outer Hebrides, Scotland
D
Grey Seal
Orkney Islands, Scotland
I
Grey Seal
Iceland
NA
Grey Seal
NW Atlantic – Canada (Combined)
I
Grey Seal
Eastern Shore, Nova Scotia
I
Grey Seal
Sable Island, Nova Scotia
I
Grey Seal
Gulf of St. Lawrence, Canada
I
Guadalupe Fur Seal
Pacific – Guadalupe Island
I
Harbour Porpoise
North Sea
NA
Harbour Porpoise
San Francisco – Russian River Stock
I
Harbour Porpoise
Monterey Bay Stock, California
NS
Harbour Porpoise
Morro Bay Stock, California
NS
Harbour Seal
England (Combined)
I
Harbour Seal
E Scotland
NS
Harbour Seal
The Wash, England
I
Harbour Seal
Wadden Sea
I
Harbour Seal
N Scotland
D
Harbour Seal
Grand Manan Island, New Brunswick
I
Harbour Seal
Oregon
I
Harbour Seal
Washington (Coast)
I

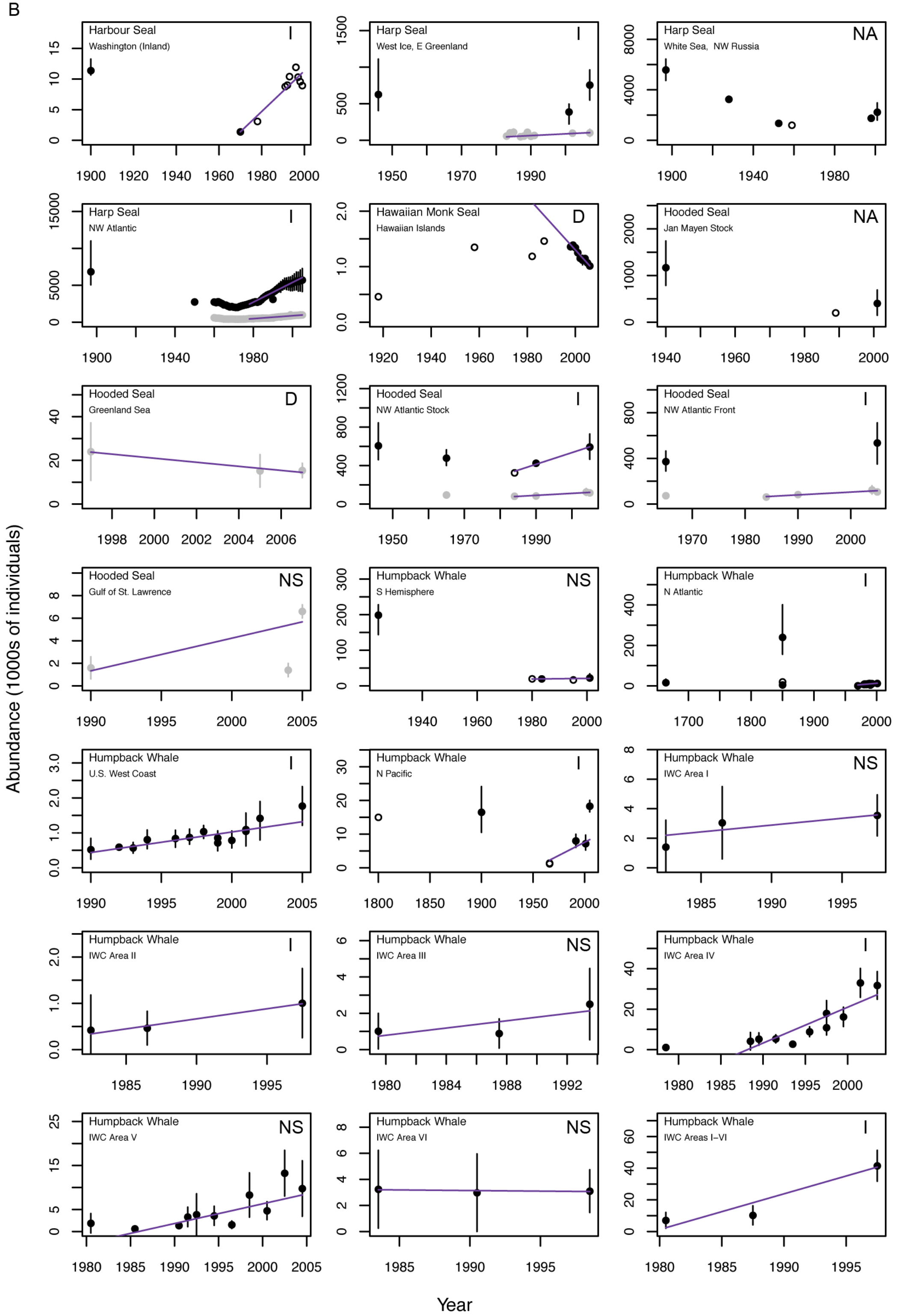
B
Harbour Seal
Washington (Inland)
I
Harp Seal
West Ice, E Greenland
I
Harp Seal
White Sea, NW Russia
NA
Harp Seal
NW Atlantic
I
Hawaiian Monk Seal
Hawaiian Islands
D
Hooded Seal
Jan Mayen Stock
NA
Hooded Seal
Greenland Sea
D
Hooded Seal
NW Atlantic Stock
I
Hooded Seal
NW Atlantic Front
I
Hooded Seal
Gulf of St. Lawrence
NS
Humpback Whale
S Hemisphere
NS
Humpback Whale
N Atlantic
I
Humpback Whale
U.S. West Coast
I
Humpback Whale
N Pacific
I
Humpback Whale
IWC Area I
NS
Humpback Whale
IWC Area II
I
Humpback Whale
IWC Area III
NS
Humpback Whale
IWC Area IV
I
Humpback Whale
IWC Area V
NS
Humpback Whale
IWC Area VI
NS
Humpback Whale
IWC Areas I–VI
I
Abundance (1000s of individuals)
Year

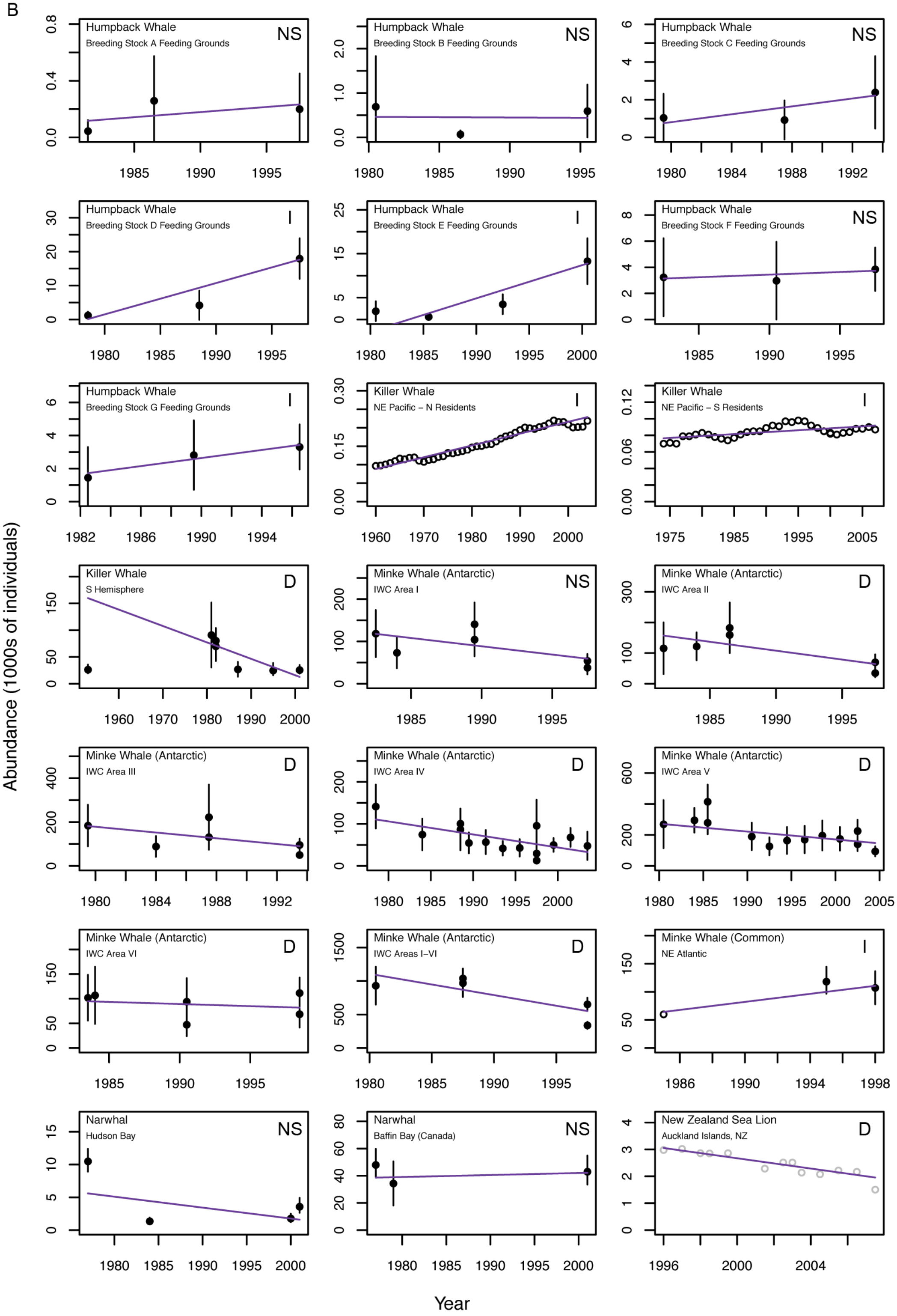

B
Humpback Whale
Breeding Stock A Feeding Grounds
NS
Humpback Whale
Breeding Stock B Feeding Grounds
NS
Humpback Whale
Breeding Stock C Feeding Grounds
NS
Humpback Whale
Breeding Stock D Feeding Grounds
I
Humpback Whale
Breeding Stock E Feeding Grounds
I
Humpback Whale
Breeding Stock F Feeding Grounds
NS
Humpback Whale
Breeding Stock G Feeding Grounds
I
Killer Whale
NE Pacific – N Residents
I
Killer Whale
NE Pacific – S Residents
I
Killer Whale
S Hemisphere
D
Minke Whale (Antarctic)
IWC Area I
NS
Minke Whale (Antarctic)
IWC Area II
D
Minke Whale (Antarctic)
IWC Area III
D
Minke Whale (Antarctic)
IWC Area IV
D
Minke Whale (Antarctic)
IWC Area V
D
Minke Whale (Antarctic)
IWC Area VI
D
Minke Whale (Antarctic)
IWC Areas I–VI
D
Minke Whale (Common)
NE Atlantic
I
Narwhal
Hudson Bay
NS
Narwhal
Baffin Bay (Canada)
NS
New Zealand Sea Lion
Auckland Islands, NZ
D
Abundance (1000s of individuals)
Year

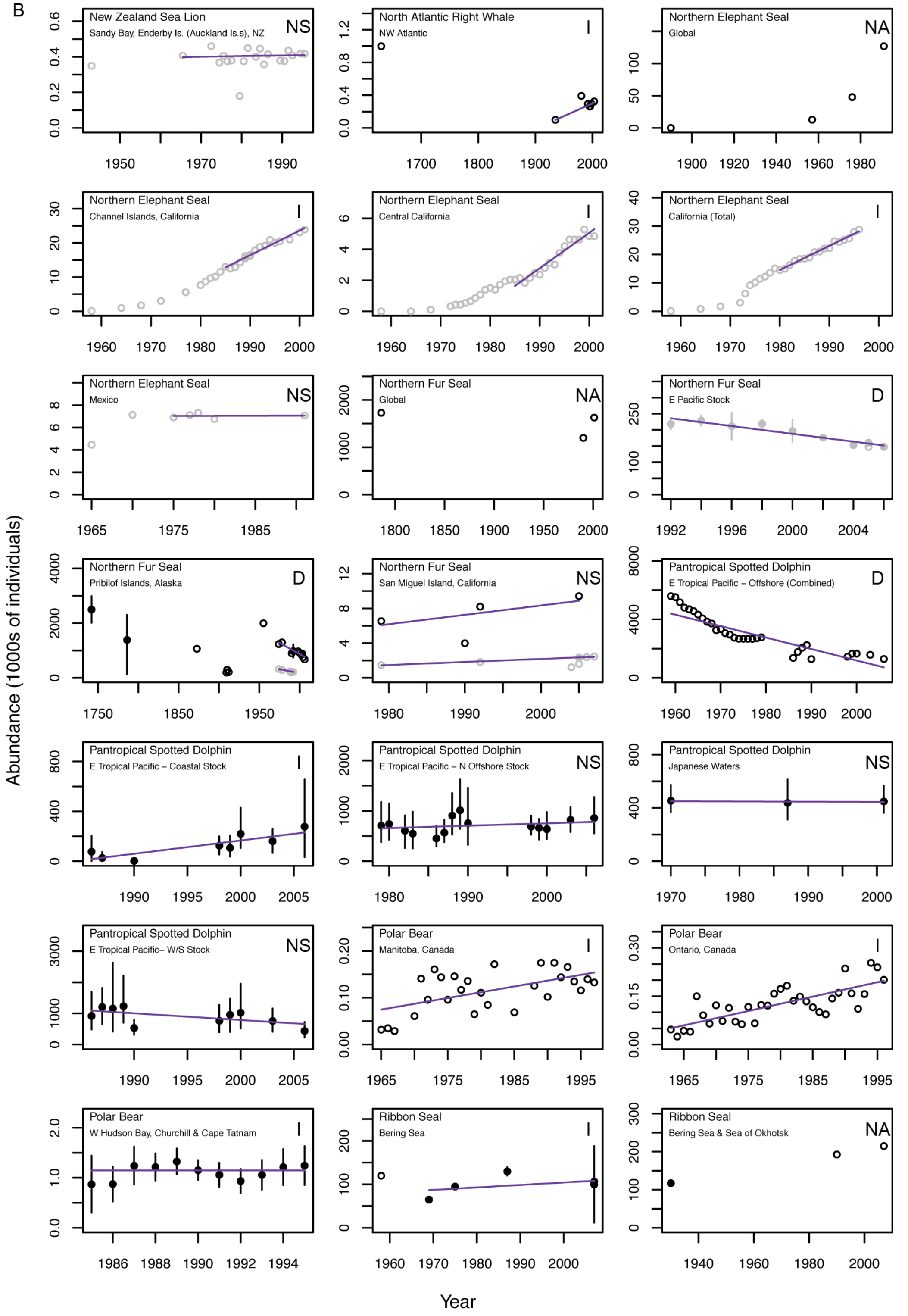
B
Abundance (1000s of individuals)
Year
New Zealand Sea Lion
Sandy Bay, Enderby Is. (Auckland Is.s), NZ
NS
North Atlantic Right Whale
NW Atlantic
I
Northern Elephant Seal
Global
NA
Northern Elephant Seal
Channel Islands, California
I
Northern Elephant Seal
Central California
I
Northern Elephant Seal
California (Total)
I
Northern Elephant Seal
Mexico
NS
Northern Fur Seal
Global
NA
Northern Fur Seal
E Pacific Stock
D
Northern Fur Seal
Pribilof Islands, Alaska
D
Northern Fur Seal
San Miguel Island, California
NS
Pantropical Spotted Dolphin
E Tropical Pacific – Offshore (Combined)
D
Pantropical Spotted Dolphin
E Tropical Pacific – Coastal Stock
I
Pantropical Spotted Dolphin
E Tropical Pacific – N Offshore Stock
NS
Pantropical Spotted Dolphin
Japanese Waters
NS
Pantropical Spotted Dolphin
E Tropical Pacific– W/S Stock
NS
Polar Bear
Manitoba, Canada
I
Polar Bear
Ontario, Canada
I
Polar Bear
W Hudson Bay, Churchill & Cape Tatnam
I
Ribbon Seal
Bering Sea
I
Ribbon Seal
Bering Sea & Sea of Okhotsk
NA

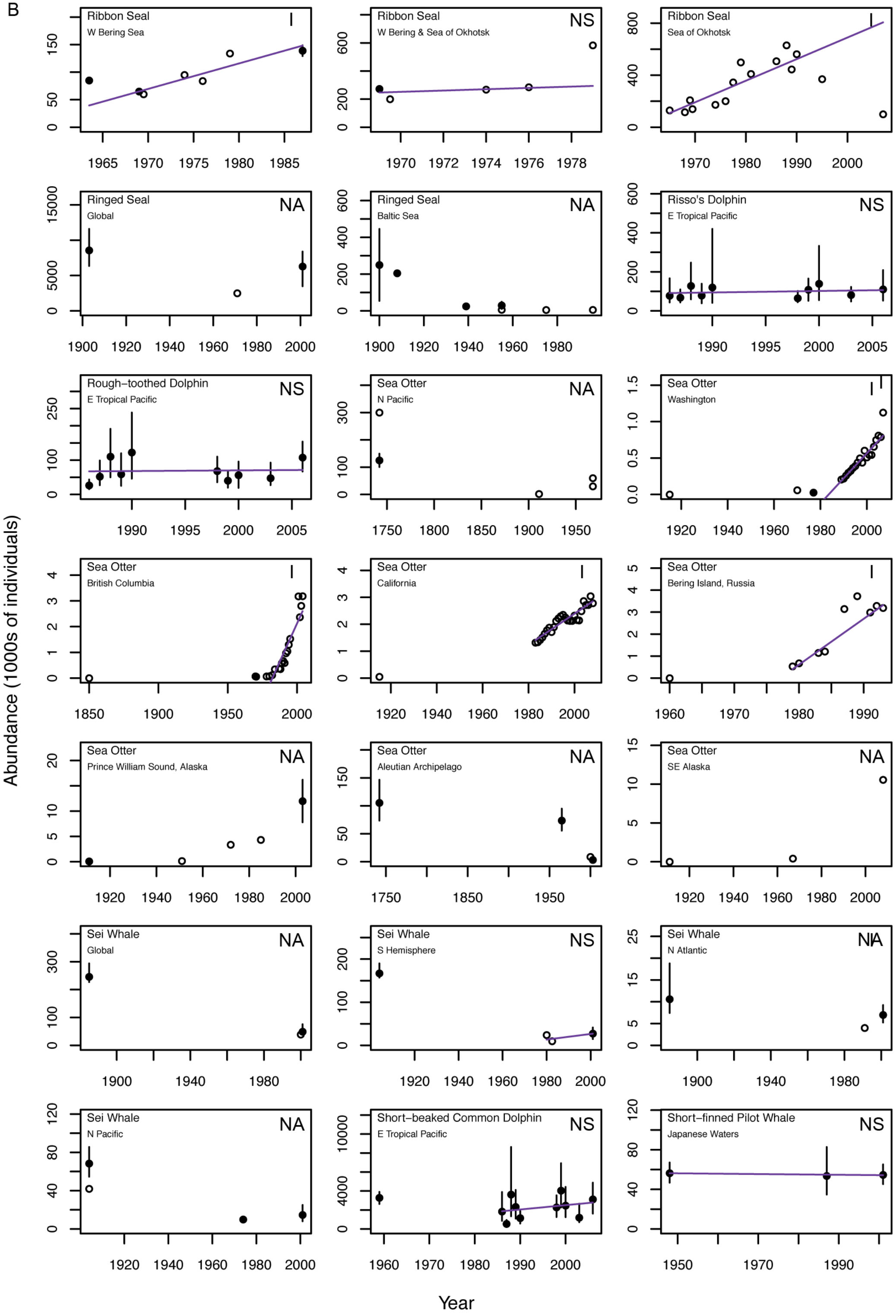
B
Ribbon Seal
W Bering Sea
I
Ribbon Seal
W Bering & Sea of Okhotsk
NS
Ribbon Seal
Sea of Okhotsk
I
Ringed Seal
Global
NA
Ringed Seal
Baltic Sea
NA
Risso's Dolphin
E Tropical Pacific
NS
Rough−toothed Dolphin
E Tropical Pacific
NS
Sea Otter
N Pacific
NA
Sea Otter
Washington
I
Sea Otter
British Columbia
I
Sea Otter
California
I
Sea Otter
Bering Island, Russia
I
Sea Otter
Prince William Sound, Alaska
NA
Sea Otter
Aleutian Archipelago
NA
Sea Otter
SE Alaska
NA
Sei Whale
Global
NA
Sei Whale
S Hemisphere
NS
Sei Whale
N Atlantic
NA
Sei Whale
N Pacific
NA
Short−beaked Common Dolphin
E Tropical Pacific
NS
Short−finned Pilot Whale
Japanese Waters
NS
Abundance (1000s of individuals)
Year

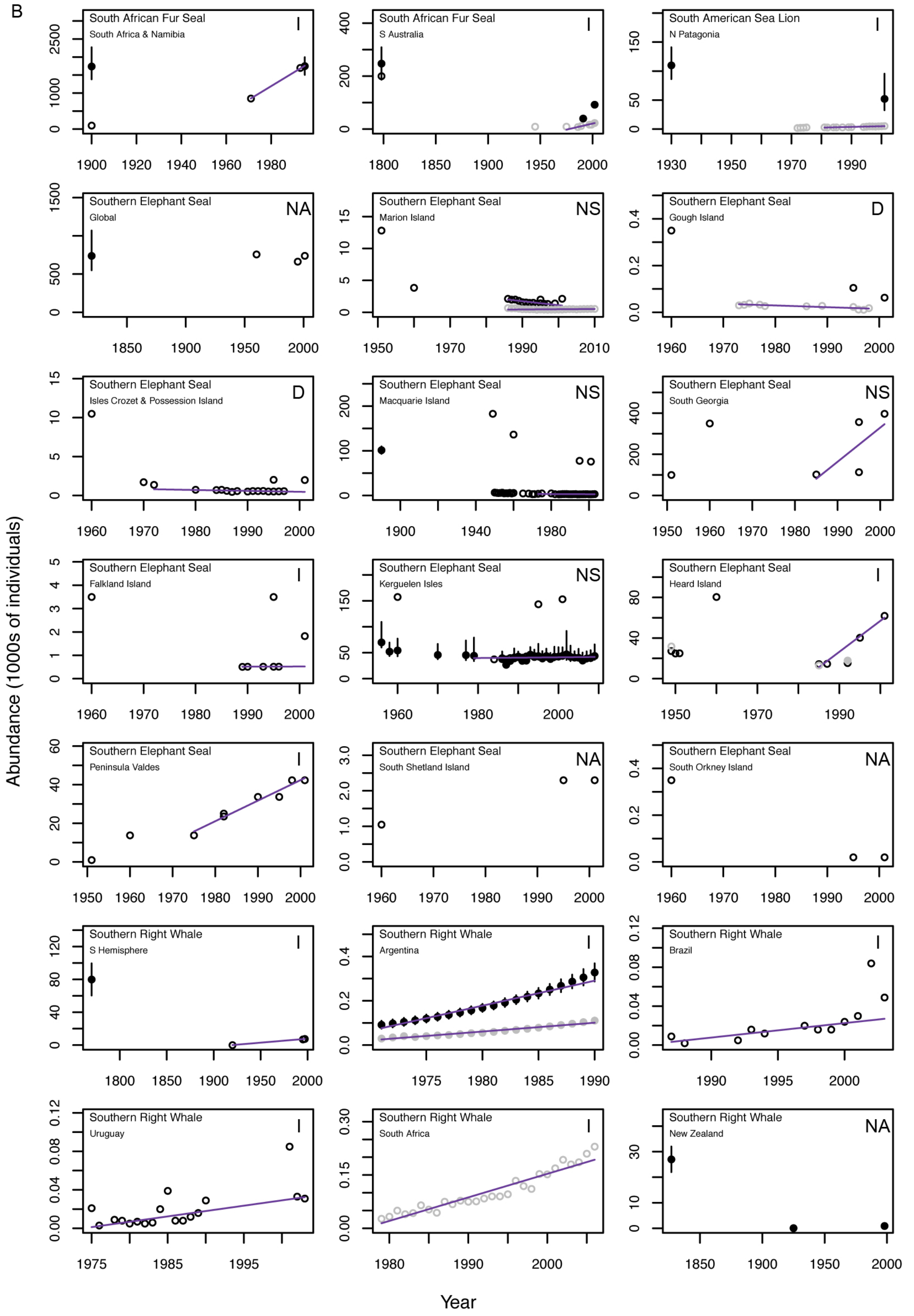
B
South African Fur Seal
South Africa & Namibia
I
South African Fur Seal
S Australia
I
South American Sea Lion
N Patagonia
I
Southern Elephant Seal
Global
NA
Southern Elephant Seal
Marion Island
NS
Southern Elephant Seal
Gough Island
D
Southern Elephant Seal
Isles Crozet & Possession Island
D
Southern Elephant Seal
Macquarie Island
NS
Southern Elephant Seal
South Georgia
NS
Southern Elephant Seal
Falkland Island
I
Southern Elephant Seal
Kerguelen Isles
NS
Southern Elephant Seal
Heard Island
I
Southern Elephant Seal
Peninsula Valdes
I
Southern Elephant Seal
South Shetland Island
NA
Southern Elephant Seal
South Orkney Island
NA
Southern Right Whale
S Hemisphere
I
Southern Right Whale
Argentina
I
Southern Right Whale
Brazil
I
Southern Right Whale
Uruguay
I
Southern Right Whale
South Africa
I
Southern Right Whale
New Zealand
NA
Abundance (1000s of individuals)
Year

B

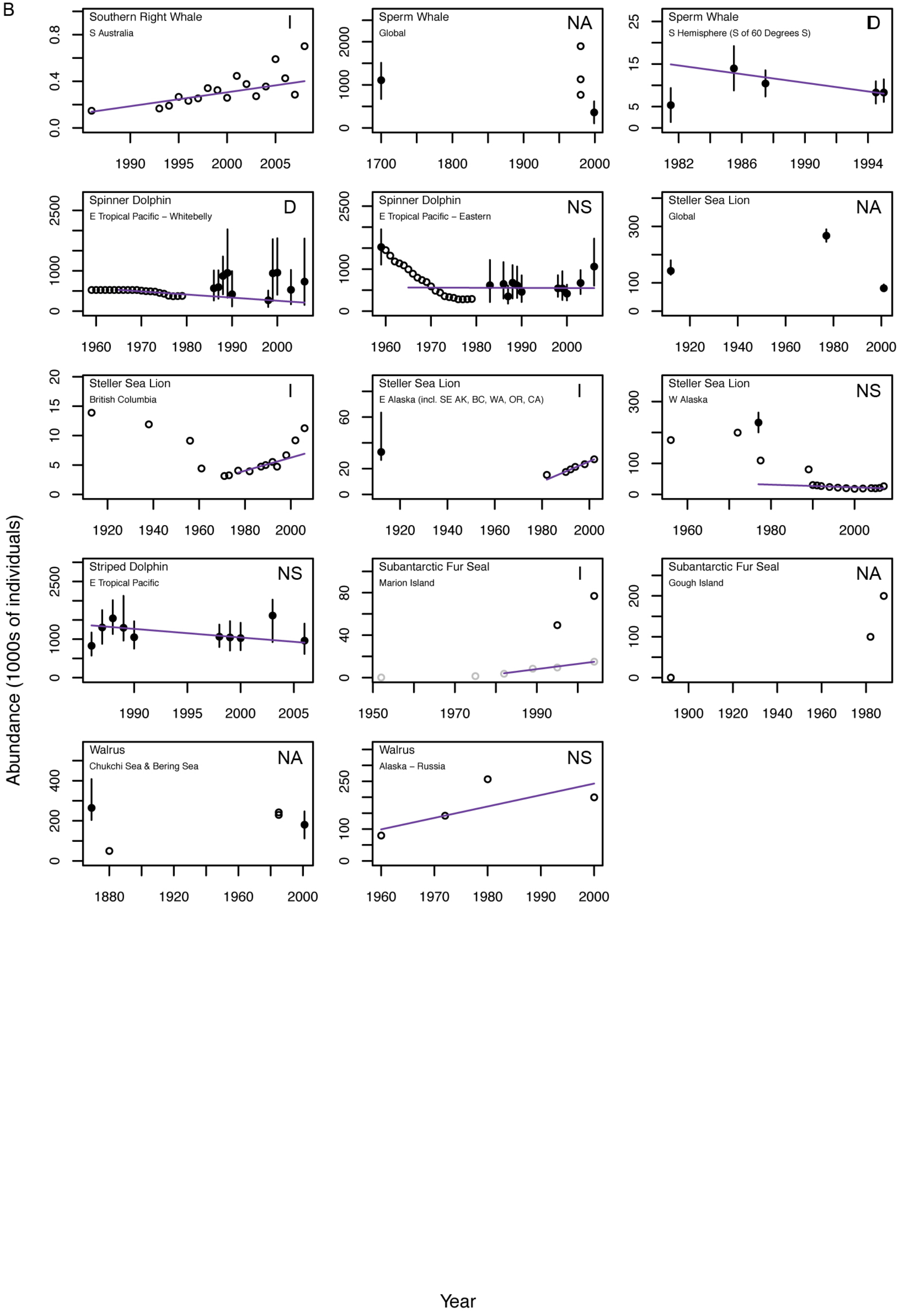

Southern Right Whale
S Australia
I
Sperm Whale
Global
NA
Sperm Whale
S Hemisphere (S of 60 Degrees S)
D
Spinner Dolphin
E Tropical Pacific – Whitebelly
D
Spinner Dolphin
E Tropical Pacific – Eastern
NS
Steller Sea Lion
Global
NA
Steller Sea Lion
British Columbia
I
Steller Sea Lion
E Alaska (incl. SE AK, BC, WA, OR, CA)
I
Steller Sea Lion
W Alaska
NS
Striped Dolphin
E Tropical Pacific
NS
Subantarctic Fur Seal
Marion Island
I
Subantarctic Fur Seal
Gough Island
NA
Walrus
Chukchi Sea & Bering Sea
NA
Walrus
Alaska – Russia
NS
Abundance (1000s of individuals)
Year
