# Supplementary material for: Recovery Trends in Marine Mammal Populations
Source: PLoS One. 2013 Oct 30;8(10):e77908. doi: 10.1371/journal.pone.0077908 (PMC3813518; doi:10.1371/journal.pone.0077908)
Supplement: Table S1 — Abundance data source information for study populations. For each population, denoted by a numeric area code (Population Area ID) and area description, abundance data sources are listed along with the data collection and/or additional analysis methods used to obtain the population abundance estimates. Abundance Confidence ID (ACID) provides an uncertainty rank (1 to 6, 1 = lowest, 6 = highest). CPUE = catch per unit effort. (DOC) [file pone.0077908.s002.doc]

**Table S1. Abundance data source information for study populations**

Each population is denoted by a numeric area code (Population Area ID) and area description. Abundance data sources are listed along with the data collection and/or additional analysis methods used to obtain the population abundance estimates. Abundance Confidence ID (ACID) provides an uncertainty rank (1 to 6, 1=lowest, 6=highest). CPUE = catch per unit effort.

| Species common name | Species scientific name | Popul-ation  Area ID | Population area description | Abundance estimate sources | Abundance data collection & analysis methods (as reported in sources) | Abundance Confidence IDs (ACIDs) |
| --- | --- | --- | --- | --- | --- | --- |
| Afro-Australian fur seal | *Arctocephalus pusillus* | 41000 | South Africa & Namibia | [[1](#_ENREF_1),[2](#_ENREF_2),[3](#_ENREF_3),[4](#_ENREF_4),[5](#_ENREF_5),[6](#_ENREF_6)] | catch or CPUE, dedicated observer data, Bayesian analysis, hindcasting, unknown | 4, 6 |
| Afro-Australian fur seal | *Arctocephalus pusillus* | 92000 | Southern Australia | [[3](#_ENREF_3),[7](#_ENREF_7),[8](#_ENREF_8),[9](#_ENREF_9),[10](#_ENREF_10),[11](#_ENREF_11),[12](#_ENREF_12),[13](#_ENREF_13)] | extrapolation based on pup production, aerial surveys, land-based surveys | 2, 3, 4, 6 |
| Antarctic fur seal | *Arctocephalus gazella* | 60000 | Antarctica | [[1](#_ENREF_1),[2](#_ENREF_2),[9](#_ENREF_9),[14](#_ENREF_14),[15](#_ENREF_15),[16](#_ENREF_16),[17](#_ENREF_17)] | dedicated observer data, unknown | 2, 6 |
| Bearded seal | *Erignathus barbatus* | 31302 | Bering Sea & Chukchi Sea | [[1](#_ENREF_1),[18](#_ENREF_18),[19](#_ENREF_19),[20](#_ENREF_20)] | catch or CPUE, Bayesian analysis, hindcasting, unknown | 2, 3, 4 |
| Beluga whale | *Delphinapterus leucas* | 10000 | Global | [[1](#_ENREF_1),[21](#_ENREF_21),[22](#_ENREF_22)] | catch or CPUE, Bayesian analysis, hindcasting, combined total based on literature, unknown | 2, 3, 4, 5 |
| Beluga whale | *Delphinapterus leucas* | 11001 | Cumberland Sound (Southeast Baffin Island) | [[21](#_ENREF_21),[23](#_ENREF_23),[24](#_ENREF_24),[25](#_ENREF_25),[26](#_ENREF_26)] | aerial surveys, on land census, unknown | 2, 5, 6 |
|  |  |  |  |  |  |  |
| Beluga whale | *Delphinapterus leucas* | 11500 | Eastern High Arctic – Baffin Bay | [[23](#_ENREF_23),[24](#_ENREF_24),[27](#_ENREF_27),[28](#_ENREF_28)] | aerial surveys | 2, 3 |
|  |  |  |  |  |  |  |
| Beluga whale | *Delphinapterus leucas* | 22112 | Eastern Hudson Bay | [[1](#_ENREF_1),[29](#_ENREF_29),[30](#_ENREF_30),[31](#_ENREF_31)] | aerial surveys, modelling approach | 2, 3, 4, 5, 6 |
| Beluga whale | *Delphinapterus leucas* | 22119 | James Bay | [[31](#_ENREF_31)] | aerial surveys, modelling approach | 2, 3, 5 |
|  |  |  |  |  |  |  |
| Beluga whale | *Delphinapterus leucas* | 36004 | Cook Inlet, Alaska | [[32](#_ENREF_32)] | aerial surveys | 2, 3 |
|  |  |  |  |  |  |  |
| Beluga whale | *Delphinapterus leucas* | 36005 | Bristol Bay, Alaska | [[18](#_ENREF_18),[32](#_ENREF_32)] | aerial surveys, unknown | 2, 4, 5 |
| Blue whale | *Balaenoptera musculus* | 12000 | Southern Hemisphere | [[1](#_ENREF_1),[19](#_ENREF_19),[33](#_ENREF_33)] | catch or CPUE, Bayesian analysis, hindcasting, unknown | 2, 3, 4, 6 |
| Blue whale | *Balaenoptera musculus* | 20000 | North Atlantic | [[1](#_ENREF_1),[34](#_ENREF_34),[35](#_ENREF_35)] | catch or CPUE, Bayesian analysis, hindcasting, unknown | 2, 3, 4 |
| Blue whale | *Balaenoptera musculus* | 30000 | North Pacific | [[1](#_ENREF_1),[19](#_ENREF_19),[34](#_ENREF_34),[35](#_ENREF_35),[36](#_ENREF_36),[37](#_ENREF_37)] | catch or CPUE, ship &/or aerial surveys, photo-identification & mark-recapture model, Bayesian analysis, hindcasting, unknown | 2, 3, 4, 6 |
| Blue whale | *Balaenoptera musculus* | 60000 | Antarctica | [[38](#_ENREF_38),[39](#_ENREF_39)] | ship &/or aerial surveys, Bayesian analysis | 2, 3 |
| Blue whale | *Balaenoptera musculus* | 60016 | IWC Area I | [[40](#_ENREF_40)] | ship surveys, modelling approach | 3 |
|  |  |  |  |  |  |  |
| Blue whale | *Balaenoptera musculus* | 60017 | IWC Area II | [[40](#_ENREF_40)] | ship surveys, modelling approach | 3 |
|  |  |  |  |  |  |  |
| Blue whale | *Balaenoptera musculus* | 60018 | IWC Area III | [[40](#_ENREF_40)] | ship surveys, modelling approach | 2, 3 |
|  |  |  |  |  |  |  |
| Blue whale | *Balaenoptera musculus* | 60019 | IWC Area IV | [[40](#_ENREF_40)] | ship surveys, modelling approach | 2, 3 |
|  |  |  |  |  |  |  |
| Blue whale | *Balaenoptera musculus* | 60020 | IWC Area V | [[40](#_ENREF_40)] | ship surveys, modelling approach | 2, 3 |
|  |  |  |  |  |  |  |
| Blue whale | *Balaenoptera musculus* | 60021 | IWC Area VI | [[40](#_ENREF_40)] | ship surveys, modelling approach | 2, 3 |
|  |  |  |  |  |  |  |
| Blue whale | *Balaenoptera musculus* | 60022 | IWC Area I-VI | [[40](#_ENREF_40)] | ship surveys, modelling approach | 2 |
|  |  |  |  |  |  |  |
| Bowhead whale | *Balaena mysticetus* | 11000 | Arctic Basin | [[1](#_ENREF_1),[41](#_ENREF_41),[42](#_ENREF_42),[43](#_ENREF_43),[44](#_ENREF_44)] | catch or CPUE, bycatch study, Bayesian analysis, hindcasting | 2, 6 |
| Bowhead whale | *Balaena mysticetus* | 22200 | Davis Strait | [[24](#_ENREF_24),[44](#_ENREF_44),[45](#_ENREF_45),[46](#_ENREF_46)] | combined total based on literature, photo-identification & mark-recapture model, unknown | 4, 6 |
|  |  |  |  |  |  |  |
| Bowhead whale | *Balaena mysticetus* | 31304 | Western Arctic | [[41](#_ENREF_41),[47](#_ENREF_47),[48](#_ENREF_48),[49](#_ENREF_49),[50](#_ENREF_50),[51](#_ENREF_51)] | on land census, acoustic surveys, extrapolation, unknown | 2, 3, 4 |
| Bryde’s whale | *Balaenoptera edeni* | 12000 | Southern Hemisphere | [[1](#_ENREF_1),[52](#_ENREF_52),[53](#_ENREF_53)] | catch or CPUE, Bayesian analysis, hindcasting, unknown | 2, 6 |
| Bryde’s whale | *Balaenoptera edeni* | 32000 | Western North Pacific | [[54](#_ENREF_54),[55](#_ENREF_55),[56](#_ENREF_56),[57](#_ENREF_57)] | catch or CPUE, ship surveys | 2, 5 |
| California sea lion | *Zalophus californianus* | 10000 | Global | [[1](#_ENREF_1),[58](#_ENREF_58)] | catch or CPUE, on land census, Bayesian analysis, hindcasting, unknown | 2, 3, 6 |
|  |  |  |  |  |  |  |
| California sea lion | *Zalophus californianus* | 31000 | Northeast Pacific (California) | [[1](#_ENREF_1),[59](#_ENREF_59)] | catch or CPUE, extrapolation based on pup production, regression analysis, Bayesian analysis, hindcasting | 2, 4, 5 |
| Common bottlenose dolphin | *Tursiops truncatus* | 17500 | Eastern Tropical Pacific | [[60](#_ENREF_60)] | ship surveys | 3 |
|  |  |  |  |  |  |  |
| Fin whale | *Balaenoptera physalus* | 12000 | Southern Hemisphere | [[1](#_ENREF_1),[19](#_ENREF_19)] | catch or CPUE, on land census, Bayesian analysis, hindcasting | 2, 3, 4 |
| Fin whale | *Balaenoptera physalus* | 20000 | North Atlantic | [[19](#_ENREF_19),[61](#_ENREF_61),[62](#_ENREF_62)] | genetic analysis, hindcasting, unknown | 2, 5, 6 |
| Fin whale | *Balaenoptera physalus* | 30000 | North Pacific | [[1](#_ENREF_1),[19](#_ENREF_19)] | catch or CPUE, on land census, Bayesian analysis, hindcasting | 2, 3 |
| Fin whale | *Balaenoptera physalus* | 60019 | IWC Area IV | [[40](#_ENREF_40)] | ship survey, modelling approach | 2, 3 |
|  |  |  |  |  |  |  |
| Fin whale | *Balaenoptera physalus* | 60020 | IWC Area V | [[40](#_ENREF_40)] | ship survey, modelling approach | 2 |
|  |  |  |  |  |  |  |
| Fin whale | *Balaenoptera physalus* | 60023 | IWC Area IIIE | [[40](#_ENREF_40)] | ship survey, modelling approach | 2, 3 |
|  |  |  |  |  |  |  |
| Fin whale | *Balaenoptera physalus* | 60025 | IWC Area IIIE, IV, V, VI | [[40](#_ENREF_40)] | ship survey, modelling approach | 2, 3 |
|  |  |  |  |  |  |  |
| Florida manatee | *Trichechus manatus* | 22900 | Florida (East and West Coasts) | [[63](#_ENREF_63)] | land & aerial surveys | 4 |
|  |  |  |  |  |  |  |
| Gray whale | *Eschrichtius robustus* | 31000 | Northeast Pacific | [[64](#_ENREF_64),[65](#_ENREF_65),[66](#_ENREF_66),[67](#_ENREF_67),[68](#_ENREF_68),[69](#_ENREF_69),[70](#_ENREF_70),[71](#_ENREF_71),[72](#_ENREF_72)] | combined total based on literature, land survey, genetic analysis, unknown | 1, 2, 5, 6 |
| Gray whale | *Eschrichtius robustus* | 32000 | Northwest Pacific | [[49](#_ENREF_49),[72](#_ENREF_72),[73](#_ENREF_73),[74](#_ENREF_74)] | combined total based on literature, hindcasting, unknown | 4, 5 |
| Grey seal | *Halichoerus grypus* | 21000 | Great Britain (Combined) | [[9](#_ENREF_9),[75](#_ENREF_75),[76](#_ENREF_76),[77](#_ENREF_77)] | extrapolation based on pup production, age or stage- based modelling, Bayesian analysis, unknown | 2, 6 |
| Grey seal | *Halichoerus grypus* | 21100 | Baltic Sea | [[9](#_ENREF_9),[78](#_ENREF_78),[79](#_ENREF_79),[80](#_ENREF_80),[81](#_ENREF_81),[82](#_ENREF_82)] | catch or CPUE, bycatch study, hindcasting, age or stage-based modelling | 2, 3, 4, 5 |
| Grey seal | *Halichoerus grypus* | 21110 | Schleswig-Holstein, Germany | [[83](#_ENREF_83),[84](#_ENREF_84)] | ship &/or aerial surveys, unknown | 5, 6 |
| Grey seal | *Halichoerus grypus* | 21221 | Netherlands | [[83](#_ENREF_83),[84](#_ENREF_84)] | ship &/or aerial surveys, unknown | 5, 6 |
|  |  |  |  |  |  |  |
| Grey seal | *Halichoerus grypus* | 21250 | North Sea | [[75](#_ENREF_75)] | extrapolation based on pup production, age or stage-based model, Bayesian analysis | 2, 3 |
| Grey seal | *Halichoerus grypus* | 21260 | Inner Hebrides, Scotland | [[75](#_ENREF_75)] | extrapolation based on pup production, age or stage-based model, Bayesian analysis | 2 |
| Grey seal | *Halichoerus grypus* | 21270 | Outer Hebrides, Scotland | [[75](#_ENREF_75)] | extrapolation based on pup production, age or stage-based model, Bayesian analysis | 2 |
| Grey seal | *Halichoerus grypus* | 21280 | Orkney Islands, Scotland | [[75](#_ENREF_75)] | extrapolation based on pup production, age or stage-based model, Bayesian analysis | 1, 2, 3 |
| Grey seal | *Halichoerus grypus* | 21530 | Iceland | [[1](#_ENREF_1),[9](#_ENREF_9),[85](#_ENREF_85)] | catch or CPUE, Bayesian analysis, hindcasting, unknown | 2, 3, 6 |
| Grey seal | *Halichoerus grypus* | 22000 | Northwest Atlantic – Canada (Combined) | [[86](#_ENREF_86),[87](#_ENREF_87),[88](#_ENREF_88),[89](#_ENREF_89)] | extrapolation based on pup production, age or stage-based model, Bayesian analysis, unknown | 2, 4, 6 |
| Grey seal | *Halichoerus grypus* | 22141 | Eastern Shore, Nova Scotia | [[86](#_ENREF_86)] | extrapolation based on pup production, age or stage-based model, Bayesian analysis | 2, 3 |
| Grey seal | *Halichoerus grypus* | 22142 | Sable Island, Nova Scotia | [[86](#_ENREF_86)] | extrapolation based on pup production, age or stage-based model, Bayesian analysis | 2 |
| Grey seal | *Halichoerus grypus* | 22300 | Gulf of St. Lawrence, Canada | [[86](#_ENREF_86)] | extrapolation based on pup production, age or stage-based model, Bayesian analysis | 2 |
| Guadalupe fur seal | *Arctocephalus townsendi* | 17000 | Pacific – Guadalupe Island | [[59](#_ENREF_59)] | on land census | 6 |
| Harbour porpoise | *Phocoena phocoena* | 21250 | North Sea | [[1](#_ENREF_1),[22](#_ENREF_22),[90](#_ENREF_90),[91](#_ENREF_91)] | catch or CPUE, dedicated observer programs, on land census, Bayesian analysis, hindcasting | 2 |
| Harbour porpoise | *Phocoena phocoena* | 31012 | San Francisco – Russian River Stock, California | [[59](#_ENREF_59)] | aerial & ship surveys | 3 |
| Harbour porpoise | *Phocoena phocoena* | 31013 | Monterey Bay Stock, California | [[59](#_ENREF_59)] | aerial & ship surveys | 3 |
| Harbour porpoise | *Phocoena phocoena* | 31014 | Morro Bay Stock, California | [[59](#_ENREF_59)] | aerial & ship surveys | 3 |
| Harbour seal | *Phoca vitulina* | 21001 | England (Combined) | [[92](#_ENREF_92)] | aerial surveys, regression analysis | 4 |
| Harbour seal | *Phoca vitulina* | 21002 | Eastern Scotland | [[92](#_ENREF_92)] | aerial surveys, regression analysis | 4 |
| Harbour seal | *Phoca vitulina* | 21007 | The Wash, England | [[92](#_ENREF_92)] | aerial surveys, regression analysis | 4 |
|  |  |  |  |  |  |  |
| Harbour seal | *Phoca vitulina* | 21211 | Wadden Sea | [[93](#_ENREF_93),[94](#_ENREF_94)] | aerial surveys, hindcasting | 4 |
| Harbour seal | *Phoca vitulina* | 21280 | Northern Scotland | [[92](#_ENREF_92)] | aerial surveys, regression analysis | 4 |
| Harbour seal | *Phoca vitulina* | 22122 | Grand Manan Island, New Brunswick | [[95](#_ENREF_95)] | aerial survey | 4 |
| Harbour seal | *Phoca vitulina* | 31001 | Oregon | [[96](#_ENREF_96)] | aerial surveys, deterministic model | 2, 4 |
| Harbour seal | *Phoca vitulina* | 31002 | Washington (Coast) | [[97](#_ENREF_97)] | aerial surveys, deterministic model | 2, 4 |
| Harbour seal | *Phoca vitulina* | 31008 | Washington (Inland) | [[97](#_ENREF_97)] | aerial surveys, deterministic model | 2, 4 |
| Harp seal | *Pagophilus groenlandicus* | 20000 | West Ice, Eastern Greenland | [[98](#_ENREF_98)] | catch or CPUE, aerial surveys, extrapolation based on pup production, Bayesian analysis, hindcasting, unknown | 2, 3, 4 |
|  |  |  |  |  |  |  |
| Harp seal | *Pagophilus groenlandicus* | 21520 | White Sea, Northwest Russia | [[1](#_ENREF_1),[99](#_ENREF_99),[100](#_ENREF_100)] | catch or CPUE, aerial surveys, extrapolation based on pup production, Bayesian analysis, hindcasting | 2, 3, 4 |
| Harp seal | *Pagophilus groenlandicus* | 22000 | Northwest Atlantic | [[101](#_ENREF_101),[102](#_ENREF_102),[103](#_ENREF_103),[104](#_ENREF_104),[105](#_ENREF_105),[106](#_ENREF_106)] | catch or CPUE, dedicated observer programs, aerial surveys, extrapolation based on pup production, deterministic model, Bayesian analysis, hindcasting, unknown | 2, 3 |
| Hawaiian monk seal | *Monachus schauinslandi* | 50000 | Hawaiian Islands | [[9](#_ENREF_9),[59](#_ENREF_59),[107](#_ENREF_107),[108](#_ENREF_108),[109](#_ENREF_109),[110](#_ENREF_110)] | land surveys, extrapolation, unknown | 4, 6 |
| Hooded seal | *Cystophora cristata* | 21000 | Jan Mayen Stock | [[1](#_ENREF_1),[9](#_ENREF_9),[111](#_ENREF_111)] | catch or CPUE, on land census, extrapolation based on pup production, Bayesian analysis, hindcasting, unknown | 3, 4 |
| Hooded seal | *Cystophora cristata* | 21400 | Greenland Sea | [[98](#_ENREF_98)] | aerial surveys, extrapolation based on pup production | 2 |
| Hooded seal | *Cystophora cristata* | 22000 | Northwest Atlantic Stock | [[1](#_ENREF_1),[9](#_ENREF_9),[104](#_ENREF_104),[112](#_ENREF_112)] | catch or CPUE, dedicated observer programs, aerial surveys, extrapolation based on pup production, Bayesian analysis, hindcasting | 2, 3, 4, |
| Hooded seal | *Cystophora cristata* | 22130 | Northwest Atlantic Front | [[112](#_ENREF_112)] | aerial survey, extrapolation based on pup production | 2 |
| Hooded seal | *Cystophora cristata* | 22301 | Gulf of St. Lawrence | [[112](#_ENREF_112),[113](#_ENREF_113)] | aerial survey, extrapolation based on pup production | 1, 2, 3 |
| Humpback whale | *Megaptera novaeangliae* | 12000 | Southern Hemisphere | [[1](#_ENREF_1),[19](#_ENREF_19),[52](#_ENREF_52),[114](#_ENREF_114)] | catch or CPUE, dedicated observer programs, Bayesian analysis, hindcasting, unknown | 2, 3, 4, 6 |
|  |  |  |  |  |  |  |
| Humpback whale | *Megaptera novaeangliae* | 20000 | North Atlantic | [[1](#_ENREF_1),[19](#_ENREF_19),[52](#_ENREF_52),[61](#_ENREF_61),[104](#_ENREF_104),[115](#_ENREF_115),[116](#_ENREF_116),[117](#_ENREF_117),[118](#_ENREF_118),[119](#_ENREF_119)] | photo-identification & mark-recapture models, catch or CPUE, dedicated observer programs, genetic analysis, extrapolation, Bayesian analysis, hindcasting, unknown | 1, 2, 3, 4, 5, 6 |
| Humpback whale | *Megaptera novaeangliae* | 31007 | U.S. West Coast | [[47](#_ENREF_47),[120](#_ENREF_120),[121](#_ENREF_121),[122](#_ENREF_122)] | photo-identification & mark-recapture models, dedicated observer programs, ship surveys | 1, 2 |
| Humpback whale | *Megaptera novaeangliae* | 35000 | North Pacific | [[1](#_ENREF_1),[123](#_ENREF_123),[124](#_ENREF_124),[125](#_ENREF_125)] | catch or CPUE, dedicated observer programs, photo-identification & mark-recapture analysis, genetic analysis, extrapolation, Bayesian analysis, hindcasting, unspecified modelling approach, unknown | 2, 3, 4, 6 |
| Humpback whale | *Megaptera novaeangliae* | 60016 | IWC Area I | [[40](#_ENREF_40),[126](#_ENREF_126)] | ship surveys, modelling approach | 1, 2, 3 |
|  |  |  |  |  |  |  |
| Humpback whale | *Megaptera novaeangliae* | 60017 | IWC Area II | [[40](#_ENREF_40),[126](#_ENREF_126)] | ship surveys, modelling approach | 2, 3 |
|  |  |  |  |  |  |  |
| Humpback whale | *Megaptera novaeangliae* | 60018 | IWC Area III | [[40](#_ENREF_40),[126](#_ENREF_126)] | ship surveys, modelling approach | 2 |
|  |  |  |  |  |  |  |
| Humpback whale | *Megaptera novaeangliae* | 60019 | IWC Area IV | [[40](#_ENREF_40),[126](#_ENREF_126),[127](#_ENREF_127)] | ship surveys, modelling approach | 1, 2, 3 |
|  |  |  |  |  |  |  |
| Humpback whale | *Megaptera novaeangliae* | 60020 | IWC Area V | [[40](#_ENREF_40),[126](#_ENREF_126),[127](#_ENREF_127)] | ship surveys, modelling approach | 1, 2, 3 |
|  |  |  |  |  |  |  |
| Humpback whale | *Megaptera novaeangliae* | 60021 | IWC Area VI | [[40](#_ENREF_40),[126](#_ENREF_126)] | ship surveys, modelling approach | 2, 3 |
|  |  |  |  |  |  |  |
| Humpback whale | *Megaptera novaeangliae* | 60022 | IWC Area I-VI | [[40](#_ENREF_40),[126](#_ENREF_126)] | ship surveys, modelling approach | 1, 2 |
|  |  |  |  |  |  |  |
| Humpback whale | *Megaptera novaeangliae* | 60026 | Breeding Stock A Feeding Grounds | [[40](#_ENREF_40),[126](#_ENREF_126)] | ship surveys, modelling approach | 3 |
|  |  |  |  |  |  |  |
| Humpback whale | *Megaptera novaeangliae* | 60027 | Breeding Stock B Feeding Grounds | [[40](#_ENREF_40),[126](#_ENREF_126)] | ship surveys, modelling approach | 3 |
|  |  |  |  |  |  |  |
| Humpback whale | *Megaptera novaeangliae* | 60028 | Breeding Stock C Feeding Grounds | [[40](#_ENREF_40),[126](#_ENREF_126)] | ship surveys, modelling approach | 2, 3 |
|  |  |  |  |  |  |  |
| Humpback whale | *Megaptera novaeangliae* | 60029 | Breeding Stock D Feeding Grounds | [[40](#_ENREF_40),[126](#_ENREF_126)] | ship surveys, modelling approach | 1, 2, 3 |
|  |  |  |  |  |  |  |
| Humpback whale | *Megaptera novaeangliae* | 60030 | Breeding Stock E Feeding Grounds | [[40](#_ENREF_40),[126](#_ENREF_126)] | ship surveys, modelling approach | 2, 3 |
|  |  |  |  |  |  |  |
| Humpback whale | *Megaptera novaeangliae* | 60031 | Breeding Stock F Feeding Grounds | [[40](#_ENREF_40),[126](#_ENREF_126)] | ship surveys, modelling approach | 1, 2, 3 |
|  |  |  |  |  |  |  |
| Humpback whale | *Megaptera novaeangliae* | 60032 | Breeding Stock G Feeding Grounds | [[40](#_ENREF_40),[126](#_ENREF_126)] | ship surveys, modelling approach | 2, 3 |
|  |  |  |  |  |  |  |
| Killer whale | *Orcinus orca* | 31006 | Eastern North Pacific – Northern Residents | [[59](#_ENREF_59)] | photo-identification & mark-recapture analysis | 3 |
| Killer whale | *Orcinus orca* | 31007 | Eastern North Pacific – Southern Residents | [[59](#_ENREF_59),[128](#_ENREF_128)] | photo-identification & mark-recapture analysis | 3 |
| Killer whale | *Orcinus orca* | 60000 | Southern Hemisphere | [[1](#_ENREF_1),[22](#_ENREF_22),[129](#_ENREF_129),[130](#_ENREF_130)] | catch or CPUE, dedicated observer programs, ship surveys, photo-identification & mark-recapture analysis, Bayesian analysis, hindcasting | 1, 2 |
| Minke whale (Common) | *Balaenoptera acutorostrata* | 21000 | Northeast Atlantic | [[131](#_ENREF_131),[132](#_ENREF_132)] | photo-identification, extrapolation, unknown | 2, 4 |
| Minke whale (Antarctic) | *Balaenoptera bonaerensis* | 60016 | IWC Area I | [[40](#_ENREF_40),[133](#_ENREF_133),[134](#_ENREF_134)] | ship surveys, modelling approach | 2 |
| Minke whale (Antarctic) | *Balaenoptera bonaerensis* | 60017 | IWC Area II | [[40](#_ENREF_40),[133](#_ENREF_133),[134](#_ENREF_134)] | ship surveys, modelling approach | 2 |
| Minke whale (Antarctic) | *Balaenoptera bonaerensis* | 60018 | IWC Area III | [[40](#_ENREF_40),[133](#_ENREF_133),[134](#_ENREF_134)] | ship surveys, modelling approach | 2 |
| Minke whale (Antarctic) | *Balaenoptera bonaerensis* | 60019 | IWC Area IV | [[40](#_ENREF_40),[133](#_ENREF_133),[134](#_ENREF_134),[135](#_ENREF_135)] | ship surveys, modelling approach | 2 |
| Minke whale (Antarctic) | *Balaenoptera bonaerensis* | 60020 | IWC Area V | [[40](#_ENREF_40),[133](#_ENREF_133),[134](#_ENREF_134),[135](#_ENREF_135)] | ship surveys, modelling approach | 2 |
| Minke whale (Antarctic) | *Balaenoptera bonaerensis* | 60021 | IWC Area VI | [[40](#_ENREF_40),[133](#_ENREF_133),[134](#_ENREF_134)] | ship surveys, modelling approach | 2 |
| Minke whale (Antarctic) | *Balaenoptera bonaerensis* | 60022 | IWC Area I-VI | [[40](#_ENREF_40),[133](#_ENREF_133),[134](#_ENREF_134)] | ship surveys, modelling approach | 2 |
| Narwhal | *Monodon monoceros* | 22115 | Hudson Bay | [[1](#_ENREF_1),[136](#_ENREF_136)] | catch or CPUE, aerial surveys, Bayesian analysis, hindcasting | 2, 3 |
|  |  |  |  |  |  |  |
| Narwhal | *Monodon monoceros* | 21400 | Baffin Bay | [[1](#_ENREF_1),[136](#_ENREF_136)] | catch or CPUE, aerial surveys, Bayesian analysis, hindcasting | 2, 3 |
| New Zealand sea lion | *Phocarctos hookeri* | 70007 | Auckland Islands, NZ | [[137](#_ENREF_137)] | unknown | 6 |
| New Zealand sea lion | *Phocarctos hookeri* | 70008 | Sandy Bay, Enderby Island (Auckland Islands), NZ | [[138](#_ENREF_138)] | photo-identification& mark-recapture study, unknown | 4, 6 |
| North Atlantic right whale | *Eubalaena glacialis* | 22000 | Northwest Atlantic | [[1](#_ENREF_1),[104](#_ENREF_104),[132](#_ENREF_132),[139](#_ENREF_139),[140](#_ENREF_140)] | catch or CPUE, aerial surveys, photo-identification & mark-recapture analysis, extrapolation, combined total based on literature, Bayesian analysis, hindcasting | 3, 4 |
| Northern elephant seal | *Mirounga angustirostris* | 10000 | Global | [[9](#_ENREF_9),[141](#_ENREF_141),[142](#_ENREF_142),[143](#_ENREF_143)] | combined total based on literature, extrapolated, unknown | 3, 6 |
|  |  |  |  |  |  |  |
| Northern elephant seal | *Mirounga angustirostris* | 31009 | Channel Islands, California | [[121](#_ENREF_121)] | unknown | 6 |
| Northern elephant seal | *Mirounga angustirostris* | 31010 | Central California | [[121](#_ENREF_121)] | unknown | 6 |
| Northern elephant seal | *Mirounga angustirostris* | 31011 | California (Total) | [[121](#_ENREF_121),[143](#_ENREF_143)] | dedicated observer programs, unknown | 2, 6 |
| Northern elephant seal | *Mirounga angustirostris* | 31015 | Mexico | [[143](#_ENREF_143)] | dedicated observer programs | 5 |
|  |  |  |  |  |  |  |
| Northern fur seal | *Callorhinus ursinus* | 10000 | Global | [[1](#_ENREF_1),[9](#_ENREF_9),[142](#_ENREF_142)] | catch or CPUE, combined total based on literature, Bayesian analysis, hindcasting, unknown | 4, 6 |
| Northern fur seal | *Callorhinus ursinus* | 17500 | Eastern Pacific Stock | [[18](#_ENREF_18),[47](#_ENREF_47),[121](#_ENREF_121)] | unknown | 4, 6 |
| Northern fur seal | *Callorhinus ursinus* | 31003 | Pribilof Islands, Alaska | [[1](#_ENREF_1),[144](#_ENREF_144),[145](#_ENREF_145),[146](#_ENREF_146),[147](#_ENREF_147),[148](#_ENREF_148),[149](#_ENREF_149)] | catch or CPUE, aerial surveys, extrapolation based on pup production, Bayesian analysis, hindcasting, unknown | 3, 4, 6 |
|  |  |  |  |  |  |  |
| Northern fur seal | *Callorhinus ursinus* | 31004 | San Miguel Island, California | [[9](#_ENREF_9),[147](#_ENREF_147),[148](#_ENREF_148),[150](#_ENREF_150),[151](#_ENREF_151)] | extrapolation based on pup production, unknown | 4, 6 |
| Pantropical spotted dolphin | *Stenella attenuata* | 17500 | Eastern Tropical Pacific - Offshore (Combined) | [[60](#_ENREF_60),[152](#_ENREF_152)] | catch or CPUE, bycatch study, ship surveys, extrapolation, hindcasting | 4 |
| Pantropical spotted dolphin | *Stenella attenuata* | 17501 | Eastern Tropical Pacific – Coastal Stock | [[60](#_ENREF_60)] | ship surveys | 3 |
| Pantropical spotted dolphin | *Stenella attenuata* | 31000 | Eastern Tropical Pacific – Northern Offshore Stock | [[60](#_ENREF_60),[153](#_ENREF_153)] | ship surveys | 2, 3 |
| Pantropical spotted dolphin | *Stenella attenuata* | 32400 | Japanese Waters | [[1](#_ENREF_1),[154](#_ENREF_154),[155](#_ENREF_155)] | catch or CPUE, ship surveys, Bayesian analysis, hindcasting | 2 |
|  |  |  |  |  |  |  |
| Pantropical  Spotted dolphin | *Stenella attenuata* | 52000 | Eastern Tropical Pacific – Western/ Southern Stock | [[60](#_ENREF_60)] | ship surveys | 3 |
| Polar bear | *Ursus maritimus* | 22113 | Manitoba, Canada | [[156](#_ENREF_156)] | aerial surveys | 4 |
| Polar bear | *Ursus maritimus* | 22114 | Ontario, Canada | [[156](#_ENREF_156)] | aerial surveys | 4 |
|  |  |  |  |  |  |  |
| Polar bear | *Ursus maritimus* | 22115 | Western Hudson Bay, Churchill & Cape Tatnam Study Area (Manitoba, Canada) | [[157](#_ENREF_157)] | photo-identification& mark-recapture study | 1, 2 |
| Ribbon seal | *Histriophoca fasciata* | 31100 | Bering Sea | [[18](#_ENREF_18),[158](#_ENREF_158),[159](#_ENREF_159),[160](#_ENREF_160)] | aerial surveys, extrapolation, unknown | 3, 4, 6 |
| Ribbon seal | *Histriophoca fasciata* | 31101 | Bering Sea & Sea of Okhotsk | [[9](#_ENREF_9),[158](#_ENREF_158)] | aerial surveys, extrapolation, unknown | 2, 4, 6 |
| Ribbon seal | *Histriophoca fasciata* | 31102 | Western Bering Sea | [[158](#_ENREF_158),[159](#_ENREF_159)] | aerial surveys, unknown | 3, 4 |
| Ribbon seal | *Histriophoca fasciata* | 31103 | Western Bering Sea & Sea of Okhotsk | [[158](#_ENREF_158)] | aerial surveys | 3, 4 |
| Ribbon seal | *Histriophoca fasciata* | 32200 | Sea of Okhotsk | [[158](#_ENREF_158),[159](#_ENREF_159),[161](#_ENREF_161)] | aerial surveys, extrapolation, unknown | 4, 6 |
|  |  |  |  |  |  |  |
| Ringed seal | *Pusa hispida* | 10000 | Global | [[1](#_ENREF_1),[9](#_ENREF_9),[142](#_ENREF_142),[162](#_ENREF_162)] | catch or CPUE, combined estimate based on literature, Bayesian analysis, hindcasting, unknown | 2, 4, 6 |
| Ringed seal | *Pusa hispida* | 21100 | Baltic Sea | [[9](#_ENREF_9),[78](#_ENREF_78),[79](#_ENREF_79),[162](#_ENREF_162),[163](#_ENREF_163)] | catch or CPUE, aerial surveys, age or stage-based modelling, hindcasting, unknown | 3, 4 |
| Risso’s dolphin | *Grampus griseus* | 17500 | Eastern Tropical Pacific | [[60](#_ENREF_60)] | ship surveys | 3 |
| Rough-toothed dolphin | *Steno bredanensis* | 17500 | Eastern Tropical Pacific | [[60](#_ENREF_60)] | ship surveys | 3 |
| Sea otter | *Enhydra lutris* | 30000 | North Pacific | [[164](#_ENREF_164),[165](#_ENREF_165),[166](#_ENREF_166)] | unknown | 4, 6 |
| Sea otter | *Enhydra lutris* | 31002 | Washington | [[167](#_ENREF_167)] | aerial, land & ship surveys, unspecified modelling approach, unknown | 2, 4, 6 |
|  |  |  |  |  |  |  |
| Sea otter | *Enhydra lutris* | 31005 | British Columbia | [[164](#_ENREF_164),[168](#_ENREF_168),[169](#_ENREF_169),[170](#_ENREF_170)] | aerial & ship surveys, unknown | 4, 6 |
| Sea otter | *Enhydra lutris* | 31011 | California | [[171](#_ENREF_171),[172](#_ENREF_172),[173](#_ENREF_173)] | aerial & land surveys, unknown | 4, 6 |
| Sea otter | *Enhydra lutris* | 31100 | Bering Island, Russia | [[174](#_ENREF_174)] | aerial surveys, unknown | 4, 5 |
| Sea otter | *Enhydra lutris* | 36001 | Prince William Sound, Alaska | [[174](#_ENREF_174),[175](#_ENREF_175)] | aerial & land surveys, unknown | 4 |
| Sea otter | *Enhydra lutris* | 38000 | Aleutian Archipelago | [[176](#_ENREF_176),[177](#_ENREF_177),[178](#_ENREF_178)] | aerial & ship surveys, unknown | 2, 4 |
| Sea otter | *Enhydra lutris* | 41000 | Southeast Alaska | [[179](#_ENREF_179)] | Unknown | 6 |
| Sei whale | *Balaenoptera borealis* | 10000 | Global | [[1](#_ENREF_1),[142](#_ENREF_142)] | catch or CPUE, combined total based on literature, Bayesian analysis, hindcasting | 2, 3, 4 |
| Sei whale | *Balaenoptera borealis* | 12000 | Southern Hemisphere | [[1](#_ENREF_1),[19](#_ENREF_19),[49](#_ENREF_49),[180](#_ENREF_180),[181](#_ENREF_181),[182](#_ENREF_182)] | catch or CPUE, ship surveys, extrapolation, Bayesian analysis, hindcasting, unknown | 2, 3, 4, 6 |
| Sei whale | *Balaenoptera borealis* | 20000 | North Atlantic | [[1](#_ENREF_1),[19](#_ENREF_19),[132](#_ENREF_132)] | catch or CPUE, Bayesian analysis, hindcasting | 3, 4 |
| Sei whale | *Balaenoptera borealis* | 30000 | North Pacific | [[1](#_ENREF_1),[183](#_ENREF_183),[184](#_ENREF_184),[185](#_ENREF_185)] | catch or CPUE, Bayesian analysis, hindcasting, unknown | 2, 3, 6 |
| Short-beaked common dolphin | *Delphinus delphis* | 17500 | Eastern Tropical Pacific | [[1](#_ENREF_1),[60](#_ENREF_60)] | catch or CPUE, ship surveys, Bayesian analysis, hindcasting | 2, 3 |
| Short-finned pilot whale | *Globicephala macrorhynchus* | 32000 | Japanese Waters | [[1](#_ENREF_1),[155](#_ENREF_155)] | catch or CPUE, ship surveys, Bayesian analysis, hindcasting | 2, 3 |
| South American sea lion | *Otaria flavescens* | 42000 | Northern Patagonia | [[1](#_ENREF_1),[186](#_ENREF_186)] | catch or CPUE, Bayesian analysis, hindcasting, unknown | 2, 3, 6 |
| Southern elephant seal | *Mirounga leonina* | 10000 | Global | [[1](#_ENREF_1),[187](#_ENREF_187)] | catch or CPUE, Bayesian analysis, hindcasting, unknown | 3, 6 |
| Southern elephant seal | *Mirounga leonina* | 60003 | Marion Island | [[187](#_ENREF_187),[188](#_ENREF_188),[189](#_ENREF_189)] | land based surveys, unknown | 5, 6 |
| Southern elephant seal | *Mirounga leonina* | 60004 | Gough Island | [[187](#_ENREF_187),[190](#_ENREF_190)] | unknown | 5, 6 |
| Southern elephant seal | *Mirounga leonina* | 60005 | Isles Crozet & Possession Island | [[187](#_ENREF_187),[191](#_ENREF_191)] | land based surveys, unknown | 5, 6 |
|  |  |  |  |  |  |  |
| Southern elephant seal | *Mirounga leonina* | 60006 | Macquarie Island | [[187](#_ENREF_187),[192](#_ENREF_192),[193](#_ENREF_193)] | land based surveys, unknown | 4, 5, 6 |
|  |  |  |  |  |  |  |
| Southern elephant seal | *Mirounga leonina* | 60007 | South Georgia | [[187](#_ENREF_187),[194](#_ENREF_194)] | land based surveys, unknown | 5, 6 |
| Southern elephant seal | *Mirounga leonina* | 60008 | Falkland Island | [[187](#_ENREF_187),[194](#_ENREF_194),[195](#_ENREF_195)] | land based surveys, unknown | 5, 6 |
| Southern elephant seal | *Mirounga leonina* | 60009 | Kerguelen Isles | [[191](#_ENREF_191),[196](#_ENREF_196)] | land based surveys, unknown | 4, 5, 6 |
| Southern elephant seal | *Mirounga leonina* | 60010 | Heard Island | [[187](#_ENREF_187),[192](#_ENREF_192),[197](#_ENREF_197)] | land based surveys, unknown | 5, 6 |
|  |  |  |  |  |  |  |
| Southern elephant seal | *Mirounga leonina* | 60011 | Peninsula Valdes | [[187](#_ENREF_187),[198](#_ENREF_198),[199](#_ENREF_199)] | unknown | 6 |
| Southern elephant seal | *Mirounga leonina* | 60012 | South Shetland Island | [[199](#_ENREF_199)] | unknown | 6 |
| Southern elephant seal | *Mirounga leonina* | 60013 | South Orkney Island | [[199](#_ENREF_199)] | unknown | 6 |
|  |  |  |  |  |  |  |
| Southern right whale | *Eubalaena australis* | 12000 | Southern Hemisphere | [[19](#_ENREF_19),[200](#_ENREF_200),[201](#_ENREF_201),[202](#_ENREF_202)] | photo-identification & mark-recapture analysis, Bayesian analysis, hindcasting, unknown | 3, 5, 6 |
| Southern right whale | *Eubalaena australis* | 70006 | New Zealand | [[203](#_ENREF_203)] | photo-identification & mark-recapture analysis, genetic analysis, Bayesian analysis, hindcasting | 1, 2, 5 |
| Sperm whale | *Physeter macrocephalus* | 10000 | Global | [[142](#_ENREF_142),[204](#_ENREF_204),[205](#_ENREF_205)] | total based on literature, extrapolation | 2, 3, 4 |
| Sperm whale | *Physeter macrocephalus* | 12001 | Southern Hemisphere (South of 60 Degrees South) | [[19](#_ENREF_19),[130](#_ENREF_130)] | ship surveys, extrapolation | 2, 3 |
| Spinner dolphin | *Stenella longirostris* | 17000 | Eastern Tropical Pacific – Whitebelly Subspecies | [[60](#_ENREF_60),[152](#_ENREF_152)] | bycatch studies, ship surveys, extrapolation, unknown | 4 |
|  |  |  |  |  |  |  |
| Spinner dolphin | *Stenella longirostris* | 17500 | Eastern Tropical Pacific – Eastern Subspecies | [[60](#_ENREF_60),[152](#_ENREF_152),[153](#_ENREF_153),[206](#_ENREF_206)] | bycatch studies, aerial & ship surveys, extrapolation, regression analysis, unknown | 2, 3, 4 |
| Steller sea lion | *Eumetopias jubatus* | 10000 | Global | [[1](#_ENREF_1),[59](#_ENREF_59)] | catch or CPUE, aerial surveys, Bayesian analysis, hindcasting | 2, 3 |
| Steller sea lion | *Eumetopias jubatus* | 31005 | British Columbia | [[9](#_ENREF_9),[207](#_ENREF_207)] | aerial surveys, unknown | 4, 6 |
| Steller sea lion | *Eumetopias jubatus* | 36002 | Eastern Alaska Stock (Includes SE AK, BC, WA, OR, CA) | [[1](#_ENREF_1),[47](#_ENREF_47)] | catch or CPUE, aerial & ship surveys, Bayesian analysis, hindcasting | 3, 4 |
| Steller sea lion | *Eumetopias jubatus* | 36003 | Western Alaska Stock | [[47](#_ENREF_47),[208](#_ENREF_208),[209](#_ENREF_209)] | aerial, ship & land based surveys, unknown | 3, 4, 6 |
| Striped dolphin | *Stenella coeruleoalba* | 17500 | Eastern Tropical Pacific | [[60](#_ENREF_60)] | ship surveys | 3 |
| Sub-antarctic fur seal | *Arctocephalus tropicalis* | 60003 | Marion Island | [[210](#_ENREF_210),[211](#_ENREF_211)] | land surveys, photo-identification & mark-recapture analysis | 4 |
| Sub-antarctic fur seal | *Arctocephalus tropicalis* | 60004 | Gough Island | [[9](#_ENREF_9),[20](#_ENREF_20)] | unknown | 6 |
| Walrus | *Odobenus rosmarus* | 31302 | Chukchi Sea & Bering Sea | [[1](#_ENREF_1),[9](#_ENREF_9),[212](#_ENREF_212)] | catch or CPUE, aerial surveys, extrapolation, Bayesian analysis, hindcasting | 2, 3, 4 |
| Walrus | *Odobenus rosmarus* | 35000 | Alaska - Russia | [[213](#_ENREF_213),[214](#_ENREF_214)] | aerial surveys, unknown | 4, 6 |

**References**

1. Christensen LB (2006) Marine mammal populations: Reconstructing historical abundance at the global scale. Vancouver: University of British Columbia Fisheries Centre. 167 p.

2. Arnould JPY (2002) Southern Fur Seals: (*Arctocephalus* sp.). In: Perrin WF, Wursig B, Thewissen JGM, editors. Encyclopedia of Marine Mammals. San Diego: Academic Press.

3. Arnould JPY, Boyd IL, Warneke RM (2003) Historical dynamics of the Australian fur seal population: evidence of regulation by man? Canadian Journal of Zoology 81: 1428-1436.

4. David J, van Sittert L (2008) A reconstruction of the Cape (South African) fur seal harvest 1653-1899 and a comparison with the 20th-century harvest. South African Journal of Science 104: 107-110.

5. Butterworth DS, Punt AE, Oosthuizen WH, Wickens PA (1995) The effects of future consumption by the Cape fur seal on catches and catch rates of the Cape hakes 3: Modelling the dynamics of the Cape fur seal *Arctocephalus pusillus pusillus*. South African Journal of Science 16: 161-183.

6. Shaughnessy PD (1982) The status of seals in South Africa and Namibia. Rome: Food and Agriculture Organization of the United Nations. 383-410 p.

7. Kirkwood R, Gales R, Terauds A, Arnould JPY, Pemberton D, et al. (2005) Pup production and population trends of the Australian fur seal (*Arctocephalus pusillus doriferus*). Marine Mammal Science 21: 260-282.

8. Warneke RM, Shaughnessy PD (1985) *Arctocephalus pusillus*, the South African and Australian fur seal: Taxonomy, evolution, biogeography, and life history. In: Ling JK, Bryden MM, editors. Studies of sea mammals in south latitudes. Adelaide, Australia: South Australian Museum. p. 53-77.

9. Reijnders P, Brasseur S, vanderToorn J, vanderWolf P, Boyd I, et al. (1993) Seals, fur seals, sea lions and walrus. Gland, Switzerland: IUCN. 98 p.

10. Goldworthy SD, Shaughnessy PD (1991) Status report on eared seals (Otariidae) in Australia and its Territories. Australia National Parks and Wildlife Service. 18 p.

11. Warneke RM (1988) Report on an aerial survey of Australian fur seal sites in Victoria and Tasmania during the 1986 breeding season. Canberra: Australian Department of the Environment and Heritage. 34 p.

12. Pemberton D, Kirkwood RJ (1994) Pup production and distribution of the Australian fur seal, *Arctocephalus pusillus*, in Tasmania. Wildlife Research 21: 341-352.

13. Pemberton D, Gales R (2004) Australian fur seals (*Arctocephalus pusillus doriferus)* breeding in Tasmania: Population size and status. Wildlife Research 31: 301-309.

14. Payne MR (1979) Growth in the Antarctic fur seal *Arctocephalus gazella*. Journal of Zoology (London) 187: 67-79.

15. Guinet C, Jouventin P, Georges J-Y (1994) Long term population changes of fur seals *Arctocephalus gazella* and *Arctocephalus tropicalis* on subantarctic (Crozet) and subtropical (St. Paul and Amsterdam) islands and their possible relationship to El Nino southern oscillation. Antarctic Science 6: 473-478.

16. Laws RM (1984) Seals. In: Laws RM, editor. Antarctic Ecology. London, UK: Academic Press. p. 621-714.

17. Knox GA (1994) Seals. In: Knox GA, editor. The biology of the southern ocean. Cambridge: Cambridge University Press. p. 141-160.

18. Angliss RP, Lodge KL (2002) Alaska Marine Mammal Stock Assessments, 2002. U.S. Department of Commerce, National Oceanic and Atmospheric Administration, National Marine Fisheries Service, Alaska Fisheries Science Center. NOAA-TM-NMFS-AFSC-133. 224 p.

19. Perry SL, DeMaster DP, Silber GK (1999) Special issue: The great whales: History and status of six species listed as endangered under the U.S. Endangered Species Act of 1973. Marine Fisheries Review 61: 1-74.

20. Bonner WN (1981) Southern Fur Seals, *Arctocephalus* (Geoffroy Saint-Hilaire and Cuvier, 1826). In: Ridgeway SH, Harrison RJ, editors. Handbook of Marine Mammals, vol 1: The walrus, sea lions, fur seals and sea otter. London: Academic Press. p. 161-208.

21. IWC (2000) Report of the Sub-Committee on Small Cetaceans - Annex I. Journal of Cetacean Research and Management 2 (Supplement): 235-262.

22. Culik B (2002) Review of Small Cetaceans: Distribution, Behaviour, Migration and Threats. UNEP and the Secretariat of CMS.

23. COSEWIC (2004) COSEWIC Assessment and Update Status Report on the Beluga Whale *Delphinapterus leucas* in Canada. Ottawa: Committee on the Status of Endangered Wildlife in Canada. ix + 71 p.

24. Mitchell ED, Reeves R (1981) Catch history and cumulative catch estimates of initial population size of cetaceans in the Canadian eastern arctic. Reports of the International Whaling Commission 31: 645-682.

25. Sergeant DE, Brodie PF (1975) Identity, abundance, and present status of populations of white whales, (*Delphinapterus leucas*) in North America. Journal of the Fisheries Research Board of Canada 32: 1047-1054.

26. Brodie PF (1971) A reconsideration of aspects of growth, reproduction, and behaviour of the white whale (*Delphinapterus leucas*) with reference to the Cumberland Sound, Baffin Island, population. Journal of the Fisheries Research Board of Canada 28: 1309-1318.

27. Richard PR, Orr JR (1986) A review of the status and harvest of white whales (*Delphinapterus leucas*) in the Cumberland Sound area, Baffin Island. 25 p.

28. Innes S, Stewart REA (2002) Population size and yield of Baffin Bay beluga (*Delphinapterus leucas*) stocks. NAMMCO. 225-238 p.

29. Reeves RR, Mitchell E (1987) Distribution and migration, exploitation, and former abundance of white whales (*Delphinapterus leucas*) in Baffin Bay and adjacent waters. 34 p.

30. Bourdages HV, Lesage V, Hammill MO, de March B (2002) Impact of harvesting on population trends of beluga in eastern Hudson Bay. Department of Fisheries and Oceans Canada. 45 p.

31. Gosselin JF, Lesage V, Hammill MO (2009) Index estimates of abundance for beluga in eastern Hudson Bay, James Bay and Ungava Bay in Summer 2008. Canadian Science Advisory Secretariat Research Document 2009/006: 25.

32. Angliss RP, Allen BM (2009) Alaska Marine Mammal Stock Assessments, 2008. U.S. Department of Commerce, National Oceanic and Atmospheric Administration, National Marine Fisheries Service, Alaska Fisheries Science Center. 269 p.

33. IWC (2000) Report of the Sub-Committee on the Comprehensive Assessment of Other Whale Stocks - Annex G. Journal of Cetacean Research and Management 2 (Suppl.): 167-208.

34. Gambell R (1976) World whale stocks. Mammal Review 6: 41-53.

35. Sears R (2002) Blue Whale: (*Balaenoptera musculus*). In: Perrin WF, Wursig B, Thewissen JGM, editors. Encyclopedia of marine mammals San Diego: Academic Press.

36. Clapham P, Young SB, Brownell RL (1999) Baleen whales: conservation issues and the status of the most endangered populations. Mammal Reviews 29: 35-60.

37. Barlow J (1995) The abundance of cetaceans in California waters: Part I. Ship surveys in summer and fall of 1991. Fishery Bulletin 93: 1-14.

38. Branch T, Matsuoka K, Miyashita T (2004) Evidence for increases in Antarctic Blue whales based on Bayesian modelling. Marine Mammal Science 20: 726-754.

39. Branch TA, Raydemeyer RA (2003) Blue whale estimates from the IDCR- SOWER surveys: Updated comparisons including results from the 1998/99 to 2000/01 surveys. Journal of Cetacean Research and Management 5 (Supplement): 291-292.

40. Leaper R, Bannister JL, Branch TA, Clapham P, Donovan GP, et al. (2008) A review of abundance, trends and foraging parameters of baleen whales in the Southern Hemisphere. CCAMLR Workshop 6: 51.

41. Woodby DA, Botkin DB (1993) Stock sizes prior to commercial whaling. Society for Marine Mammalogy. 387-407 p.

42. Hacquebord L, Leinenga R (1994) The ecology of Greenland whales in relation to whaling and climate change in the 17th and 18th centuries. Tijdschrift voor Geschiendenis (in Dutch) 107: 415-438.

43. Weslawksi JM, Hacquebord L, Stempniewicz L, Malinga M (2000) Greenland whales and walruses in the Svalbard food web before and after exploitation. Oceanologia 42: 37-56.

44. Finley KJ (2001) Natural history and conservation of the Greenland whale, or bowhead, in the northwest Atlantic. Arctic 54: 55-76.

45. Shelden KEW, Rugh DJ (1995) The bowhead whale (*Balaena mysticetus*): status review. Marine Fisheries Review 57: 1-20.

46. Finley KJ (1990) Isabella Bay, Baffin-Island - an Important Historical and Present-Day Concentration Area for the Endangered Bowhead Whale (*Balaena mysticetus*) of the Eastern Canadian Arctic. Arctic 43: 137-152.

47. Angliss RP, Outlaw RB (2008) Alaska Marine Mammal Stock Assessments, 2007. U.S. Department of Commerce, National Oceanic and Atmospheric Administration, National Marine Fisheries Service, Alaska Fisheries Science Center. NMFS-AFSC-180 NMFS-AFSC-180. 252 p.

48. George JC, Zeh J, Suydam R, Clark C (2004) Abundance and population trend (1978-2001) of western arctic bowhead whales surveyed near Barrow, Alaska. Marine Mammal Science 20: 755-773.

49. Klinowska M (1991) Dolphins, Porpoises, and Whales of the World: The IUCN Red Data Book. Gland, Switzerland: IUCN - The World Conservation Union. 429 p.

50. Tillman MF (1984) Report of the Scientific Committee. International Whaling Commission. 35-181 p.

51. Reeves RR, Leatherwood S (1985) Bowhead whale Balaena mysticetus (Linnaeus, 1758). In: Ridgway SH, Harrison RH, editors. The Sirenians and Baleen whales - Handbook of Marine Mammals. London: Academic Press. p. 305-344.

52. Tamura T, Ohsumi S (1999) Estimation of total food consumption by cetaceans in the world's ocean. Tokyo: Institute of Cetacean Research. 15 p.

53. Ohsumi S (1981) Further estimation of population sizes of Bryde's whales in the South Pacific and Indian Ocean using sighting data. Reports of the International Whaling Commission 31: 407-415.

54. IWC (1997) Report of the Sub-Committee on North Pacific Bryde's Whales - Annex G. Reports of the International Whaling Commission - (Scientific Committee). Cambridge, UK: IWC. p. 163-168.

55. Kato H (2002) Bryde's whale: (*Balaenoptera edeni* and *B. brydei*). In: Perrin WF, Wursig B, Thewissen JGM, editors. Encyclopedia of Marine Mammals. San Diego: Academic Press.

56. Holt S (1986) Aspects of the assessment and regulation of Bryde 's whales in the Northwest Pacific. Reports of the International Whaling Commission 36: 257-262.

57. Miyashita T (1986) Sighting estimate for the Bryde's whale stock in the western North Pacific. Reports of the International Whaling Commission 36: 249-252.

58. Heath CB (2002) California, Galapagos, and Japanese Sea Lions: (*Zalophus californianus*, *Z. wollebaeki*, and *Z. japonicus*) In: Perrin WF, Wursig B, Thewissen JGM, editors. Encyclopedia of Marine Mammals. San Diego: Academic Press.

59. Carretta JV, Forney KA, Lowry MS, Barlow J, Baker J, et al. (2008) U.S. Pacific Marine Mammal Stock Assessments: 2007. U.S. Department of Commerce, National Oceanic and Atmospheric Administration, National Marine Fisheries Service, Southwest Fisheries Science Center. 321 p.

60. Gerrodette T, Watters GM, Perryman W, Ballance L (2008) Estimates of 2006 Dolphin Abundance in the Eastern Tropical Pacific, with Revised Estimates from 1986-2003. U.S. Department of Commerce, National Oceanic and Atmospheric Administration, National Marine Fisheries Service. 43 p.

61. Roman J, Palumbi SR (2003) Whales before whaling in the North Atlantic. Science 301: 508-510.

62. IWC (2004) Whale population estimates. International Whaling Commission.

63. USFWS (2001) Florida Manatee Recovery Plan (*Trichechus manatus latirostris*) Third Revision. Atlanta, Georgia: U.S. Fish and Wildlife Service. 144 p. + aendices.

64. Alter SE, Rynes E, Palumbi SR (2007) DNA evidence for historic population size and past ecosystem impacts of gray whales. Proceedings of the National Academy of Sciences of the United States of America 104: 15162-15167.

65. Reilly SB (1981) Population assessment and population dynamics of the California gray whale (*Eschrichtius robustus*) [PhD]. Washington: University of Washington.

66. Rugh DJ, Hobbs RC, Lerczak JA, Breiwick JM (2005) Estimates of abundance of the eastern North Pacific stock of gray whales (*Eschrichtius robustus*) 1997-2002. Journal of Cetacean Research and Management 7: 1-12.

67. Butterworth DS, Korrubel JL, Punt AE (2002) What is needed to make a simple density-dependent response population model consistent with data for the eastern gray whales? Journal of Cetacean Research and Management 4: 63-76.

68. Scammon CM (1874) The Marine Mammals of the North-western Coast of North America, Described and Illustrated: Together with an Account of the American Whale-Fishery. San Francisco: John H. Carmany.

69. Reilly SB (1992) Population biology and status of eastern Pacific gray whales: recent developments. In: McCullough DR, Barrett RH, editors. Wildlife 2001: Populations London: Elsevier Alied Science Publishers. p. 1062-1074.

70. Henderson DA (1984) Nineteenth century gray whaling: grounds, catches and kills, practices and depletion of the whale population. In: Jones ML, Swartz SL, Leatherwood S, editors. The Gray Whale, *Eschrichtius robustus* Orlando, FL.: Academic Press, Inc. p. 159-186.

71. Wade PR, Perryman W (2002) Shimonoseki, Japan: IWC Scientific Committee. SC/54/BRG7 SC/54/BRG7.

72. Swartz SL, Taylor BL, Rugh DJ (2006) Gray whale *Eschrichtius robustus* population and stock identity. Mammal Review 1: 66-84.

73. Weller DW, Burdin AM, Würsig B, Taylor BL, Brownell RLJ (2002) The western gray whale: A review of past exploitation, current status and potential threats. Journal of Cetacean Research and Management 4: 7-12.

74. Vladimirov VL (1994) Recent distribution and abundance levels of whales in Russian far-eastern Seas. Russian Journal of Marine Biology 20: 1-9.

75. Thomas L, Harwood J (2008) Estimating the size of the UK grey seal population between 1984 and 2007. St. Andrews, UK: NERC Sea Mammal Research Unit and Centre for Research into Ecological and Environmental Modelling, University of St Andrews. 18 p.

76. Hiby L, Duck CD, Thompson D (1992) Seal stock in Great Britain: Surveys conducted in 1990 and 1991. NERC News: 30-31.

77. Beddington JR, Beverton RJH, Lavigne DMe (1985) Marine Mammals and Fisheries; Beddington JR, Beverton RJH, Lavigne DM, editors. London: George Allen & Unwin.

78. Kokko H, Helle E, Lindstroem J, Ranta E, Sipilae T, et al. (1999) Backcasting population sizes of ringed and grey seals in the Baltic and Lake Saimaa during the 20th century. Annales Zoologici Fennici 36: 65-73.

79. Harding KC, Härkönen TJ (1999) Development in the Baltic grey seal (*Halichoerus grypus*) and ringed seal (*Phoca hispida*) populations during the 20th century. Ambio 28: 619-627.

80. Stenman O, Helle E. Etat des populations de phoques dans la Mer Baltique; 1987; Oslo, Norway. p. 61-62.

81. ICES. Report of the Working Group on Marine Mammal Ecology (CM 2003/ACE); 2003; Hel, Poland. p. 95.

82. Hiby L, Lundberg T, Karlsson O, Helander B (2001) An estimate of the size of the Baltic grey seal population based on photo-id data. Lanstyrelsen I Vasternorrlands Lan and Naturvardsverket. 29-11-2001 29-11-2001.

83. TSEG (2009) Aerial surveys of grey seals in the Wadden Sea in the seasons of 2007- 2008 and 2008-2009. Trilateral Seal Expert Group (TSEG). 2 p.

84. TSEG (2002) Common and grey seals in the Wadden Sea. Trilateral Seal Expert Group - Plus. 1-96 p.

85. Hauksson E. The status of the Icelandic seal population in 1986-1990; 1987; Oslo, Norway. p. 91-104.

86. Thomas L, Hammill MO, Bowen WD (2008) Assessment of Population Consequences of Harvest Strategies for the Northwest Atlantic grey seal population. Fisheries and Oceans Canada. 13 p.

87. Waring GT, Quintal JM, Fairfield CP, Clapham PJ, Cole TVN, et al. (2003) U.S. Atlantic and Gulf of Mexico Marine Mammal Stock Assessments - 2003. U.S. Department of Commerce, National Oceanic and Atmospheric Administration, National Marine Fisheries Service Northeast Fisheries Science Center. 300 p.

88. Anonymous (1986) Seals and Sealing in Canada. Royal Commission on Seals and the Sealing Industry in Canada.

89. Waring GT, Josephson E, Fairfield Walsh CP, Maze-Foley K (2005) U.S. Atlantic and Gulf of Mexico Marine Mammal Stock Assessments 2008. U.S. Department of Commerce, National Oceanic and Atmospheric Administration, National Marine Fisheries Service Northeast Fisheries Science Center. 358 p.

90. Hammond PS, Berggren P, Benke H, Borchers DL, Collet A, et al. (2002) Abundance of harbour porpoise and other cetaceans in the North Sea and adjacent waters. Journal of Alied Ecology 39: 361-376.

91. Read AJ (1999) Harbour propoise *Phocoena phocoena* (Linnaeus, 1758). In: Ridgway SH, Harrison RH, editors. The Second Book of Dolphins and the Porpoises - Handbook of Marine Mammals. London: Academic Press. p. 323-355.

92. Lonergan M, Duck CD, Thompson D, Mackey BL, Cunningham L, et al. (2007) Using sparse survey data to investigate the declining abundance of British harbour seals. Journal of Zoology 271: 261-269.

93. Reineking B (2002) Phocine Distemper Epidemic Amongst Seals in 2002 Wilhelmshaven: Common Wadden Sea Secretariat. 3-8 p.

94. Reijnders PJH. Retrospective population analysis and related future management perspectives for the harbour seal *Phoca vitulina* in the Wadden Sea. In: Dankers N, Smit CJ, Scholl M, editors. 22-26 Oct., 1990; 1992 22-26 Oct., 1990; Ameland, The Netherlands. p. 193-197.

95. Fowler GM, Stobo WT (2005) Sources of Variability in Aerial Survey Counts of Harbour Seals on Haul-Out Sites in the Bay of Fundy. iv + 26 p.

96. Brown RF, Wright BE, Riemer SD, Laake J (2005) Trends in abundance and current status of harbor seals in Oregon: 1977-2003. Marine Mammal Science 21: 657-670.

97. Jeffries S, Huber H, Calambokidis J, Laake J (2003) Trends and status of harbor seals in Washington state: 1978-1999. Journal of Wildlife Management 67: 207-218.

98. ICES (2008) Report of the Working Group on Harp and Hooded Seals (WGHARP). Tromsø, Norway: International Council for the Exploration of the Sea.

99. Lavigne DM (2002) Harp Seal: (*Pagophilus groenlandicus*) In: Perrin WF, Wursig B, Thewissen JGM, editors. Encyclopedia of Marine Mammals. San Diego: Academic Press.

100. Sergeant DE (1991) Harp Seals, Man and Ice. Ottawa. 153 p.

101. Hammill MO, Stenson GB (2009) Unpublished data.

102. Roff DA, Bowen WD (1986) Further analysis of population trends in the northwest Atlantic harp seal (*Phoca groenlandica*) from 1967 to 1985. Canadian Journal of Fisheries and Aquatic Sciences 43: 553-564.

103. Hammill MO, Stenson G (2005) Abundance of Northwest Atlantic harp seals (1960-2005). Fisheries and Oceans Canada. 38 p.

104. Waring GT, Quintal JM, Fairfield CP, Clapham PJ, Cole TVN, et al. (2002) U.S. Atlantic and Gulf of Mexico Marine Mammal Stock Assessments - 2002. U.S. Department of Commerce, National Oceanic and Atmospheric Administration, National Marine Fisheries Service Northeast Fisheries Science Center. NMFS-NE-169 NMFS-NE-169. 183 p.

105. Bowen WD, Sergeant DE (1983) A mark-recapture estimate of 1983 harp seal pup production in the northwest Atlantic. Northwest Atlantic Fisheries Organization. 14 p.

106. Warren WG, Shelton PA, Stenson GB (1997) Quantifying some of the major sources of uncertainty associated with estimates of harp seal prey consumption. Part 1: Uncertainty in the estimates of harp seal population size. Journal of Northwest Atlantic Fishery Science 22: 289-302.

107. Hiruki LM, Ragen TJ (1992) A compilation of historical (*Monachus schauinslandi*) monk seal counts. US Department of Commerce.

108. Rice DW (1960) Population dynamics of the Hawaiian monk seal. Journal of Marine Mammalogy 41: 376-385.

109. Schultz JK, Baker JD, Toonen RJ, Bowen BW (2009) Extremely Low Genetic Diversity in the Endangered Hawaiian Monk Seal (Monachus schauinslandi). Journal of Heredity 100: 25-33.

110. Johnson A, Delong R, Fiscus C (1982) Population status of the Hawaiian monk seal (*Monachus schauinslandi*), 1978. Journal of Mammalogy 63: 415-421.

111. ICES. Report of the joint ICES/NAFO working group on Harp and Hooded seals (CM 1991); 1991. International Council of the Exploration of the Sea.

112. Hammill MO, Stenson G (2006) Abundance of Northwest Atlantic hooded seals (1960 – 2005) Fisheries and Oceans Canada. 23 p.

113. Hammill MO, Lydersen C, Kovacs KM, Sjare B (1997) Estimated Fish consumption by hooded seals (*Cystophora cristata*) in the Gulf of St Lawrence. Journal of Northwest Atlantic Fishery Science 22: 259-257.

114. IWC (1996) Report of the Sub-Committee on Southern Hemisphere Baleen Whales - Annex E. In: Donovan GP, editor. Reports of the International Whaling Commission. Cambridge, UK: IWC. p. 117-131.

115. Stevick PT, Allen J, Clapham PJ, Friday NA, Katona SK, et al. (2003) North Atlantic humpback whale abundance and rate of increase four decades after protection from whaling. Marine Ecology Progress Series 258: 263-273.

116. COSEWIC (2003) COSEWIC Assessment and Update Status Report on the Humpback Whale *Megaptera novaeangliae* in Canada. Ottawa: Committee on the Status of Endangered Wildlife in Canada. viii + 25 p.

117. Whitehead H (1987) Updated status of the humpback whale, *Megaptera novaeangliae*, in Canada. Canadian Field-Naturalist 101: 284-294.

118. Whitehead H, Glass C (1985) The Significance of the Southeast Shoal of the Grand Bank to Humpback Whales and Other Cetacean Species. Canadian Journal of Zoology 63: 2617-2685.

119. Mitchell E (1973) Draft report on humpback whales taken under special scientific permit by eastern Canadian land stations, 1969-1971. Report of the International Whaling Commission 23: 183-154.

120. Calambokidis J, Steiger G, Straley JM, Herman L (2001) Movements and Population Structure of Humpback Whales in the North Pacific. Marine Mammal Science 17: 769-794.

121. Carretta JV, Muto MM, Barlow J, Baker J, Forney KA, et al. (2002) U.S. Pacific Marine Mammal Stock Assessments - 2002. U.S. Department of Commerce. 290 p.

122. Calambokidis J, Barlow J (2004) Abundance of Blue and Humpback Whales in the Eastern North Pacific Estimated by Capture-Recapture and Line-Transect Methods. Marine Mammal Science 20: 63-85.

123. Calambokidis J, Steiger JM, Straley JM, Quinn II TJ, Herman LM, et al. (1997) Abundance and population structure of humpback whales in the North Pacific Basin. La Jolla, California: National Marine Fisheries Service. 71 p.

124. Johnson JH, Wolman AA (1984) The humpback whale, *Megaptera novaeangliae*. Marine Fisheries Review 46: 30-37.

125. Calambokidis J, Falcone EA, Quinn TJ, Burdin AM, Clapham PJ, et al. (2008) SPLASH: Structure of Populations, Levels of Abundance and Status of Humpback Whales in the North Pacific. Olympia, Washington: Cascadia Research. 57 p.

126. Branch T (2006) Humpback abundance south of 60 degrees South from three completed sets of IDRC/SOWER circumpolar surveys. J Cetacean Res Manage 9: 87-96.

127. Matsuoka K, Hakamada T, Kiwada H, Murase H, Nishiwaki S (2006) Distribution and abundance estimates of humpback whales in the Antarctic Areas IV and V (70 degrees E - 170 degress W). IWC Paper SC/A06/HW57: 21.

128. Ford JKB, Ellis GM, Balcomb KC (2000) Killer Whales: The Natural History and Genealogy of *Orcinus orca* in British Columbia and Washington. Vancouver, B. C. and Seattle, Washington: University of British Columbia Press and University of Washington Press.

129. Kasamatsu F, Yamamoto Y, Zenitani R, Ishikawa H, Ishibashi T, et al. (1988) Distribution of Cetacean Sightings in the Antarctic: Results Obtained from the IWC/IDCR Minke Whale Assessment Cruises, 1978/79 to 1983/84. 449-482 p.

130. Branch TA, Butterworth DS (2001) Estimates of abundance south of 60°S for cetacean species sighted frequently on the 1978/79 to 1997/98 IWC/IDCR-SOWER sighting surveys. Journal of Cetacean Research and Management 3: 251-270.

131. Skaug HJ, Øien N, Schweder T, Bøthun G (2004) Current abundance of minke whales in the northeastern Atlantic; variability in time and space. Canadian Journal of Fisheries and Aquatic Sciences 61: 870–886.

132. Tamura T, Ohsumi S (2000) Regional assessments of prey consumption by marine cetaceans in the world. 52nd meeting of the IWC Scientific Committee. 42 p.

133. Branch TA (2006) Abundance estimates for Antarctic minke whales from three completed circumpolar sets of surveys, 1978/79 to 2003/04. IWC Paper SC/58/IA18: 28.

134. Okamura H, Kitakado T (2008) Abundance estimates of Antarctic minke whales from the historical IDCR/SOWER survey data using the OK Method. IWC Paper SC/60/IA8: 19.

135. Hakamada T, Matsuoka K, Nishiwaki S (2006) Abundance trend of Antarctic minke whales in Areas IV and V based on JARPA data. IWC Paper SC/58/IA7: 4.

136. COSEWIC (2005) COSEWIC Assessment and Update Status Report on the Narwhal *Monodon monoceros* in Canada. Ottawa: Committee on the Status of Endangered Wildlife in Canada. vii + 50 p.

137. New Zealand Department of Conservation (2009) New Zealand sea lion species management plan: 2009-2014. Wellington: New Zealand Department of Conservation. 33 p.

138. Childerhouse S, Gales N (1998) Historical and modern distribution and abundance of the New Zealand sea lion *Phocarctos hookeri*. New Zealand Journal of Zoology 25: 1-16.

139. Waring GT, Josephson E, Fairfield Walsh CP, Maze-Foley K (2007) U.S. Atlantic and Gulf of Mexico Marine Mammal Stock Assessments - 2007. U.S. Department of Commerce, National Oceanic and Atmospheric Administration, National Marine Fisheries Service Northeast Fisheries Science Center. 426 p.

140. Reeves RR, Breiwick JM, Mitchell E. Pre-exploitation abundance of right whales off the eastern United States. In: Hain J, editor. N.E. Fisheries Center Document; 1992. N.E. Fisheries Center. p. 5-7.

141. Hindell MA (2002) Elephant seals: (*Mirounga angustirostris* and *M. leonina*). In: Perrin WF, Wursig B, Thewissen JGM, editors. Encyclopedia of Marine Mammals. San Diego: Academic Press.

142. Trites AW, Christensen V, Pauly D (1997) Competition Between Fisheries and Marine Mammals for Prey and Primary Production in the Pacific Ocean. Journal of Northwest Atlantic Fishery Science 22: 173-187.

143. Stewart BS, Yochem PK, Huber HR, DeLong RL, Jameson RJ, et al. (1994) History and present status of northern elephant seal population. In: Le Boeuf BJ, Laws RM, editors. Elephant Seals: Population Ecology, Behavior, and Physiology. Berkeley, Los Angeles, London: University of California Press. p. 29-48.

144. COSEWIC (2006) COSEWIC Assessment and Update Status Report on the Northern Fur Seal *Callorhinus ursinus* in Canada. Ottawa: Committee on the Status of Endangered Wildlife in Canada. vii + 33 p.

145. Roel AY, Davey SP (1965) Evolution of fur seal management on the Pribilof Islands. The Journal of Wildlife Management 29: 448-463.

146. Towell RG, Ream RR (2006) Decline in Northern Fur Seal (*Callorhinus ursinus*) pup production on the Pribilof Islands. Marine Mammal Science 22: 486-491.

147. Sinclair EH (1994) Fur Seal Investigations, 1993. U.S. Department of Commerce. 93 p.

148. Sinclair EH (1994) Fur Seal Investigations, 1992. U.S. Department of Commerce. 190 p.

149. Anonymous (2004) Northern Fur Seal Exploitation. NOAA.

150. Testa JW (2007) Fur seal investigations, 2004-2006. U.S. Department of Commerce. 76 p.

151. Testa JW (2008) Fur seal investigations, 2006-2007. U.S. Department of Commerce. 76 p.

152. Smith TD (1983) Changes in the size of three dolphin (*Stenella* sp.) populations in the Eastern Tropical Pacific. Fishery Bulletin 81: 1-13.

153. Gerrodette T, Forcada J (2005) Non-recovery of two spotted and spinner dolphin populations in the eastern tropical Pacific Ocean. Marine Ecology Progress Series 291: 1-21.

154. Miyashita T (1993) Distribution and abundance of some dolphins taken in the North Pacific driftnet fisheries. International North Pacific Fisheries Commission Bulletin 53: 435-449.

155. Miyashita T (1993) Abundance of dolphin stocks in the western North Pacific taken by the Japanese drive fishery. Reports of the International Whaling Commission 43: 417-437.

156. Stirling I, Lunn NJ, Iacozza J, Elliott C, Obbard M (2004) Polar bear distribution and abundance on the Southwestern Hudson Bay Coast during open water season, in relation to population trends and annual ice patterns. Arctic 57: 15-26.

157. Lunn NJ, Stirling I, Andriashek D, Kolenosky GB (1997) Re-estimating the size of the polar bear population in western Hudson Bay. Arctic 50: 234-240.

158. Boveng PL, Bengtson JL, Buckley TW, Cameron MF, Dahle SP, et al. (2008) Status Review of the Ribbon Seal (*Histriophoca fasciata*). U.S. Department of Commerce. 131 p.

159. Fedoseev G (2002) Ribbon seal: (*Histriophoca fasciata)*. In: Perrin WF, Wursig B, Thewissen HGM, editors. Encyclopedia of Marine Mammals. San Diego: Academic Press.

160. Burns JJ (1994) Ribbon Seal. Juneau, Alaska: Alaska Department of Fish and Game.

161. Mizuno AW, Wada A, Ishinazaka T, Hattori K, Watanabe Y, et al. (2002) Distribution and abundance of spotted seals (*Phoca largha*) and ribbon seals (*Phoca fasciata*) in the southern Sea of Okhotsk. Ecological Research 17: 79-96.

162. Frost KJ, Lowry LF (1981) Ringed, Baikal and Caspian seals, Phoca hispida Schreber 1775, Phoca siberica Gmelin 1788, Phoca caspica Gmelin 1788. In: Ridgway SH, Harrison RJ, editors. Handbook of Marine Mammals: Academic Press London. p. 29-53.

163. Härkönen TJ, Stenman O, Jüssi M, Jüssi I, Sagitov R, et al. (1998) Population size and distribution of the Baltic ringed seal (*Phoca hispida botnica*). Ringed seals in the North Atlantic. Tromsø, Norway: North Atlantic Marine Mammal Commission (NAMMCO). p. 167-180.

164. Sea Otter Recovery Team (2007) Recovery Strategy for the Sea Otter (*Enhydra lutris*) in Canada. Vancouver, B. C.: Fisheries and Oceans Canada. vii + 56 p.

165. Kenyon KW (1969) The sea otter in the eastern Pacific Ocean. North American Fauna 68: 1-352.

166. Johnson AM (1982) The sea otter, *Enhydra lutris*. 521-525 p.

167. USFWS (2008) Sea Otter (*Enhydra lutris*) Washington Stock. Lacey, Washington: U.S. Fish and Wildlife Service. 7 p.

168. Estes JA (1990) Growth and equilibrium in sea otter populations. Journal of Animal Ecology 59: 385-401.

169. Nichol LM, Watson JC, Ellis GM, Ford JKB (2005) An assessment of abundance and growth of the sea otter population (*Enhydra lutris*) in British Columbia. Nanaimo, BC: Fisheries and Oceans Canada. 22 p.

170. Watson JC (1993) The effects of the sea otter (*Enhydra lutris*) foraging on shallow rocky communities off northwestern Vancouver Island, British Columbia. [Ph.D.]. Santa Cruz: University of California. 169 p.

171. USFWS (2008) Southern Sea Otter (*Enhydra lutris nereis*). Ventura, California: U.S. Fish and Wildlife Service. 11 p.

172. Laidre KL, Jameson RJ, DeMaster DP (2001) An estimation of carrying capacity for sea otters along the California coast. Marine Mammal Science 17: 294-309.

173. Bryant HC (1916) Sea otters near Point Sur. California Department of Fish and Game. 134-135 p.

174. Bodkin JL, Jameson RJ, Estes JA (1995) Sea otters in the North Pacific Ocean. Washington, D.C.: U.S. Department of the Interior, National Biological Service. 353-356 p.

175. Bodkin JL, Monson DH (2003) Sea otter population structure and ecology on Alaska. Arctic Research of the United States 16: 31-36.

176. Estes JA, Tinker MT, Doroff AM, Burn DM (2005) Continuing sea otter population declines in the Aleutian archipelago. Marine Mammal Science 21: 169-172.

177. Burn DM, Doroff AM, Tinker MT (2003) Carrying capacity and pre-decline abundance of sea otters (*Enhydra lutris kenyoni*) in the Aleutian Islands. Northwestern Naturalist 84: 145–148.

178. Doroff AM, Estes JA, Tinker MT, Burn DM, Evans TJ (2003) Sea otter population declines in the Aleutian archipelago. Journal of Mammalogy 84: 55-64.

179. USFWS (2008) Northern Sea Otter (*Enhydra lutris kenyoni*): Southeast Alaska Stock. U.S. Fish and Wildlife Service.

180. Horwood J (2002) Sei whale: (*Balaenoptera borealis*). In: Perrin WF, Wursig B, Thewissen JGM, editors. Encyclopedia of Marine Mammals. San Diego: Academic Press.

181. Tamura T, Ohsumi S (2000) Regional assessments of prey consumption by marine cetaceans in the world. IWC Scientific Committee. 42 p.

182. Gambell R (1985) Sei Whale *Balaenoptera borealis*. In: Ridgway SH, Harrison R, editors. Handbook of Marine Mammals, Vol 3: The Sirenians and Baleen Whales. Orlando: Academic Press Inc. p. 155-170.

183. Tillman MF (1977) Estimates of population size for the North Pacific sei whale. In: G.P.Donovan, editor. Sei and Bryde's Whales - Reports of the International Whaling Commission (Special issue 1). Cambridge, UK: IWC. p. 98-106.

184. Carretta JV, Muto MM, Barlow J, Baker J, Forney KA, et al. (2001) U.S. Pacific Marine Mammal Stock Assessments - 2001. U.S. Department of Commerce. 280 p.

185. Carretta JV, Muto MM, Barlow J, Baker J, Forney KA, et al. (2003) U.S. Pacific Marine Mammal Stock Assessments - 2003. U.S. Department of Commerce. 291 p.

186. Dans SL, Crespo EA, Pedraza SN, Alonso MK (2004) Recovery of the South American sea lion (*Otaria flavescens*) population in northern Patagonia. Canadian Journal of Fisheries and Aquatic Sciences 61: 1681-1690.

187. McMahon CR, Bester MN, Burton H, Hindell MA, Bradshaw CJA (2005) Population status, trends and a re-examination of the hypotheses explaining the recent declines of the southern elephant seal *Mirounga leonina*. Mammal Review 35: 82-100.

188. Pistorius PA, Bester MN, Kirkman SP (1999) Dynamic age-distributions in a declining population of southern elephant seals. Antarctic Science 11: 445-450.

189. Pistorius P, DeBruyn P, Bester M (2011) Population dynamics of southern elephant seals: a synthesis of three decades of demographic research at Marion Island. . African Journal of Marine Science 33: 523-534.

190. Bester MN, Moller H, Wium J, Enslin B (2001) An update on the status of southern elephant seals on Gough Island. South African Journal of Wildlife Research 31: 68-71.

191. Guinet C, Jouventin P, Weimerskirch H (1999) Recent population change of the southern elephant seal at Iles Crozet and Iles Kerguélen: the end of the decrease. Antarctic Science 11: 193-197.

192. Australian Government Department of the Environment and Heritage (2004) Biology, threats and conservation status of the sub-antarctic fur seal and southern elephant seal in Australian waters. Canberra. 65 p.

193. DeLittle SC, Bradshaw CJA, McMahon CR, Hindell MA (2007) Complex interplay between intrinsic and extrinsic drivers of long-term survival trends in southern elephant seals. BMC Ecology 7: 12.

194. Boyd IL, Walker TR, Poncet J (1996) Status of southern elephant seals at South Georgia. Antarctic Science 8: 237–244.

195. Galimberti F, Boitani L (1999) Demography and breeding biology of a small, localized population of

southern elephant seals (Mirounga leonina). Marine Mammal Science 15: 159-178.

196. Authier M, Delord K, Guinet C (2011) Population trends of female Elephant Seals breeding on the Courbet Peninsula, îles Kerguelen. Polar Biology 34: 319-328.

197. Slip DJ, Burton HR (1999) Population status and seasonal haulout patterns of the southern elephant seal (*Mirounga leonina*) at Heard Island. Antarctic Science 11: 38-47.

198. Lewis M, Campagna C, Quintana F, Falabella V (1998) Estado actual y distribucion de la poblacion elefante marino del sur en la Peninsula Valdes, Argentina. Mastozoologia Neotropical 5: 29-40.

199. Laws RM (1994) History and present status of southern elephant seal populations. In: Boeuf BJL, Laws RM, editors. Elephant seals: population ecology, behavior and physiology. Berkeley: University of California Press. p. 49-65.

200. Baker CS, Clapham PJ (2004) Modelling the past and future of whales and whaling. Trends in Ecology & Evolution 19: 365-371.

201. IWC (2001) Report of the workshop on the comprehensive assessment of right whales: a worldwide comparison. Journal of Cetacean Research and Management 2: 1-61.

202. Cummings WC (1985) Right whales – *Eubalaena glacialis* (Muller, 1776) and *Eubalaena australis* (Desmoulins, 1822). In: Ridgway SH, Harrison R, editors. Handbook of Marine Mammals. London: Academic Press. p. 275-304.

203. Jackson JA, Carroll E, Smith TD, Patenaude N, Baker CS (2009) Taking Stock: the historical demography of the New Zealand right whale (the Tohora). 72 p. p.

204. Whitehead HP (2002) Estimates of the current global population size and historical trajectory for sperm whales. Marine Ecology Progress Series 242: 295-304.

205. Rice DW (1989) Sperm whale. *Physeter macrocephalus* Linnaeus, 1758. In: Ridgway SH, Harrison R, editors. Handbook of marine mammals. London: Academic Press. p. 177-233.

206. Wade PR, Gerrodette T (1993) Estimates of cetacean abundance and distribution in the Eastern Tropical Pacific. Reports of the International Whaling Commission 43: 477-493.

207. Fisheries and Oceans Canada (2008) Population Assessment: Steller Sea Lion (*Eumetopias jubatus*). Fisheries and Oceans Canada Science Pacific Region. 11 p.

208. Fritz L, Lynn M, Kunisch E, Sweeney K (2008) Aerial, Ship and Land-Based Surveys of Steller Sea Lions (*Eumetopias jubatus*) in Alaska, June and July 2005 - 2007. U.S. Department of Commerce. 70 p.

209. Trites AW, Larkin PA (1992) The Status of Steller Sea Lion Populations and the Development of Fisheries in the Gulf of Alaska and Aleutian Islands. Vancouver, B. C.: Fisheries Centre, University of British Columbia.

210. Hofmeyr GJG, Bester MN, Makhado AB, Pistorius PA (2006) Population changes in Subantarctic and Antarctic fur seals at Marion Island. South African Journal of Wildlife Research 36: 55-68.

211. Hofmeyr GJG, Bester MN, Jonker FC (1997) Changes in population sizes and distribution of fur seals at Marion Island. Polar Biology 17: 150-158.

212. Fay FH, Kelly BP, Sease JL (1989) Managing the exploitation of Pacific walruses: a tragedy of delayed response and poor communication. Marine Mammal Science 5: 1-16.

213. Kastelein RA (2002) Walrus: (*Odobenus rosmarus*) In: Perrin WF, Wursig B, Thewissen JGM, editors. Encycylopedia of Marine Mammals. San Diego: Academic Press.

214. Fay FH, Eberhardt LL, Kelly BP, Burns JJ, Quakenbush LT (1997) Status of the Pacific Walrus Population, 1950-1989. Marine Mammal Science 13: 537-565.
