# Supplementary material for: Recovery Trends in Marine Mammal Populations
Source: PLoS One. 2013 Oct 30;8(10):e77908. doi: 10.1371/journal.pone.0077908 (PMC3813518; doi:10.1371/journal.pone.0077908)
Supplement: Text S1 — Abundance confidence ID (ACID) system: explanation and verification. (DOC) [file pone.0077908.s007.doc]

**Text S1. Abundance confidence ID (ACID) system: explanation and verification.**

**1. Explanation**

We used a modified version of an Abundance Confidence ID (ACID) rating system developed by Kaschner (2004) to incorporate both qualitative (e.g. method of data collection, reliability) and, where available, quantitative (e.g. standard error, confidence interval) information into the weighting of each abundance observation in the database. This system was developed by ([Kaschner 2004](#_ENREF_5)), and allows for error information to be incorporated and compared between abundance estimates with and without quantitative statements of error. Quantitative statements ranked higher than qualitative statements of error ([Kaschner 2004](#_ENREF_5)).

Since the Abundance Confidence ID (referred to as ACID from this point forward) is an amalgam of both quantitative and qualitative information, we used Table S1.1 and the following rules to determine the ACID for each abundance observation.

- In assessing qualitative data quality, we considered the data collection or generation method, and assigned an ACID accordingly (see Table S1.2).
- We ranked the ACID of each data point based on the both the quantitative and qualitative error information available.
- When the two categories suggest differing ACIDs, we took the average of the two as the ACID.
- In cases where a mean abundance was generated from a range of values (e.g. 2000-3000 whales), and the statistics were generated in a similar way, an additional quantitative category ranking of 4 was adding to the averaging of the ACID (i.e. to produce an average of three numbers).

**Table S1.1. Abundance Confidence ID (ACID) criteria categories (based on Kaschner, 2004).** More information on assigning qualitative error is contained in Table S1.2

| **ACID** | **Label** | **Quantitative Error** | **Qualitative Error** |
| --- | --- | --- | --- |
| 1 | very high | CV < 0.2 | -Dedicated survey with known survey area (map or clearly defined area) and information about uncertainties  - Photo-identification, mark-recapture analysis or dedicated acoustic survey  - Modelling methods |
| 2 | high | 0.2 < CV < 0.5 | - Dedicated survey without definite area description or map and information about uncertainties  - Photo-identification, mark-recapture analysis or dedicated acoustic survey  - Dedicated observer program, dedicated aerial/ship/land-based survey or pup-count extrapolation  - Modelling methods |
| 3 | medium | CV > 0.5 | - Survey without area description or time period, but giving a range (e.g. minimum to maximum estimates)  - Photo-identification, mark-recapture analysis or dedicated acoustic survey without range  - Dedicated observer program, dedicated aerial/ship/land-based survey or pup-count extrapolation  - Opportunistic survey, bycatch study, catch-per-unit effort, stranding, genetic diversity analysis, bone remains, extrapolation, or total based on literature  - Modelling methods |
| 4 | low | Large min/max range OR range provided and mean abundance estimate needs to be estimated | - Very general estimate, no specific time period or area, no uncertainties (mostly secondary references)  - General global estimates  - Dedicated observer program, dedicated aerial/ship/land-based survey or pup-count extrapolation  - Opportunistic survey, bycatch study, catch-per-unit effort, stranding, genetic diversity analysis, bone remains, extrapolation, or total based on literature  - Modelling methods |
| 5 | very low | No real range provided or “guess-estimate” OR inferred from other species | - Outdated general estimates  - Single points estimate with no error, but with sampling method stated  - Opportunistic survey, bycatch study, catch-per-unit effort, stranding, genetic diversity analysis, bone remains, extrapolation, or total based on literature  - Modelling methods |
| 6 | “guess-timate” | Nothing | - Guesstimate or inferred from other species  - Single abundance estimates with no range  - No survey method recorded |

**Table S1.2. Abundance data collection or estimation methods used in deriving qualitative error rank (based on Kaschner, 2004).** The method or technique used to generate an abundance estimate is often species and situation-specific, depending on species behaviour, ease and appropriateness of sampling through different methods, population size/range, funding and cost-effectiveness. On a scale of 1:6, 1 is highest (best) rank, while 6 is the lowest (worst).

| **Sampling Method** | **Comments** | **References** | **Qualitative Error Rank** |
| --- | --- | --- | --- |
| Photo-identification or mark-recapture analysis | Useful and accurate for small populations where all individuals are accounted for and have identifiable distinguishing markers, such as distinct fin shapes or markings, (e.g. resident killer whales) and have an equal probability of being captured and re-captured (e.g. migrating humpback whales). | ([Berta et al. 2006](#_ENREF_2))  ([Forney & Wade 2006](#_ENREF_4))  ([Seber 1992](#_ENREF_8))  ([Whitehead et al. 2000](#_ENREF_11)) | 1 – 3  (depending on other error information provided) |
| Dedicated observer program, dedicated  aerial/ship/land-based survey, acoustic survey or pup-count extrapolation | Standard methods for assessing marine mammal abundances, and can be especially useful when consistently performed measurements are collected over time. Acoustic methods are especially useful for populations that are hard to observe, may occur over vast areas and communicate acoustically (e.g. sei and blue whales). | ([Berta et al. 2006](#_ENREF_2))  ([Buckland & York 2002](#_ENREF_3))  ([Forney & Wade 2006](#_ENREF_4))  ([Seber 1992](#_ENREF_8))  (Širović et al. 2009)  ([Skalski et al. 2005](#_ENREF_10))  ([Whitehead et al. 2000](#_ENREF_11)) | 2 – 4  (depending on other error information provided) |
| Opportunistic survey, bycatch study, catch-per-unit effort, stranding, genetic diversity analysis, bone remains, extrapolation, or total based on literature | Likely more difficult to obtain accurate abundance estimates over time from these methods, and historical population estimates may have large uncertainty associated with them. | ([Forney & Wade 2006](#_ENREF_4))  ([Roman & Palumbi 2003](#_ENREF_6)) | 3 – 5  (depending on other error information provided) |
| Modelling techniques | Different techniques may be more or less appropriate and accurate for estimating abundance of a population, depending on the situation and sample data available. | ([Baker & Clapham 2004](#_ENREF_1))  ([Skalski et al. 2005](#_ENREF_10))  ([S.T. Buckland et al. 2007](#_ENREF_7)) | 1 – 6  (can be any level depending on appropriateness of model for species and data available (situation specific) and resulting quantitative error estimation) |
| No stated method | NA | NA | 6 |

**2. Verification for Weighting Regressions**

We tested whether the Abundance Confidence ID (ACID) system, which allows us to incorporate both quantitative and qualitative error information, was an appropriate weighting scheme and comparable to weighting by standard error (SE). It adhered well to standard error data when it was available. We examined the population data for the populations that did have standard error information associated with their data points (n = 53 populations). We ran robust linear regressions on the scaled data, and compared the results of the regressions using two different weighting systems: (1) SE and (2) ACID. We then compared whether the trend information (i.e. Increasing, Stable, Decreasing or Unknown as denoted by the slope) and whether the results were significant or not agreed among the regressions. We found similar signals 77% of the time (Table S1.3) leading us to believe that the ACID weighting system yields a good representation of quantitative error information in the weighting of abundance data points in a regression for trend determination.

**Table S1.3. Comparisons of scaled population abundance trends (i.e. Increasing, Decreasing, Stable or Unknown), for populations with standard error information (n=53), using two types of weighting in regression: (1) SE and (2) ACID.** “Agree” and “Do Not Agree” denote whether all compared regression weighting method trend results agree or not.

| Populations | Agree | Do Not Agree | Total |
| --- | --- | --- | --- |
| N | 41 | 12 | 53 |
| % | 77 | 23 | 100 |

**References**

Baker, C. S., and P. J. Clapham. 2004. Modelling the past and future of whales and whaling. Trends in Ecology & Evolution **19**:365-371.

Berta, A., J. L. Sumich, and K. M. Kovacs. 2006. Population Structure and Dynamics. Pages 416-455. Marine Mammals - Evolutionary Biology. Academic Press, London.

Buckland, S.T., K.B. Newman, C. Fernández, L. Thomas, and J. Harwood. 2007. Embedding population dynamics models in inference. Statistical Science **22**:44-58.

Buckland, S. T., and A. E. York. 2002. Abundance Estimation in W. F. Perrin, B. Wursig, and J. G. M. Thewissen, editors. Encyclopedia of Marine Mammals. Academic Press, San Diego.

Forney, K. A., and P. R. Wade. 2006. Worldwide Distribution and Abundance of Killer Whales. Pages 145-162 in J. A. Estes, D. P. Demaster, D. F. Doak, T. M. Williams, and R. L. Brownell Jr., editors. Whales, Whaling, and Ocean Ecosystems. University of California Press, Los Angeles and London.

Kaschner, K. 2004. Modelling and Mapping Resource Overlap between Marine Mammals and Fisheries on a Global Scale. 241 pp. Department of Zoology. University of British Columbia, Vancouver.

Roman, J., and S. R. Palumbi. 2003. Whales before whaling in the North Atlantic. Science **301**:508-510.

Seber, G. A. F. 1992. A Review of Estimating Animal Abundance II International Statistical Review **60**:129-166.

Širović, A., J. A. Hildebrand, S. M. Wiggins, and D. Thiele. 2009. Blue and fin whale acoustic presence around Antarctica during 2003 and 2004. Marine Mammal Science **25**:125-136.

Skalski, J. R., K. E. Ryding, and J. J. Millspaugh 2005. Wildlife demography: Analysis of sex, age, and count data. Academic Press, San Diego.

Whitehead, H., J. Christal, and P. L. Tyack. 2000. Studying Cetacean Social Structure in Space and Time: Innovative Techniques. Pages 65-87 in J. Mann, R. C. Connor, P. L. Tyack, and H. Whitehead, editors. Cetacean Societies - Field Studies of Dolphins and Whales. University of Chicago Press, Chicago and London.
