# Supplementary material for: Recovery Trends in Marine Mammal Populations
Source: PLoS One. 2013 Oct 30;8(10):e77908. doi: 10.1371/journal.pone.0077908 (PMC3813518; doi:10.1371/journal.pone.0077908)
Supplement: Text S2 — R Statistical Software code for analyzing marine mammal population abundance trends. (DOCX) [file pone.0077908.s008.docx]

Text S2. R Statistical Software Code for Analyzing Marine Mammal Population Abundance Trends (R 2.11.1 for Mac)

##### LOAD DATA

setwd #locates working directory

setwd("~/WORKING DIRECTORY NAME/ ") #sets working directory

##### RENAME DATA FOR USE IN FUNCTION

data0 <- read.csv("ABUNDANCE DAT FILE NAME.csv")

# load species .csv file with population abundance data; load separately for each species

##### SET UP DATAFRAME & REMOVE NA

data1 <- as.data.frame(data0) # create a data frame to manipulate columns

data1_2 <- subset(data1, !is.na(data1$AbundMean))

# Or use PupCountMean instead of AbundMean if using pup count data

# remove values for which mean abundance value not available

data2 <- subset(data1_2, !is.na(data1_2$YearRead))

# remove values for which year value not available

# SPECIFY INFORMATION FOR EACH SPECIES

Species <- "gray_whale_scaled" # label -> character string name

# label data as pup count as needed to distinguish it from

# regular data

Pop_int <- c(31000, 32000) # vector of populations of interest, indicated by Area_ID

GenT <- 22.9 # generation time in years

Species_com <- "Gray Whale" # species common name

Stype <- "Cetacean" # Cetacean, Pinniped or Other

##### SET UP AN EMPTY MATRIX TO STORE REGRESSION RESULTS

# AND THEN DELIVER RESULTS TO IT

# EACH TIME A SET OF REGRESSIONS IS PERFORMED FOR A POPULATION

# calls reg.rev1() command listed later

# run ten types of regression:

# (1) linear non-scaled (lm_ns),

# (2) linear non-scaled weighted (lm_ns_w),

# (3) linear scaled (lm_sds),

# (4) linear scaled weighted (lm_sds_w),

# (5) robust scaled (lmRob_sds),

# (6) robust scaled weighted (lmRob_sds_w),

# (7) robust non-scaled (lmRob_ns),

# (8) robust non-scaled weighted (lmRob_ns_w)

# (9) log linear (abundance) regression scaled non-weighted (log_lm_sds)

# (10) log linear (abundance) regression scaled weighted (log_lm_sds_w)

# (11) robust log linear (abundance) regression scaled non-weighted (log_lmRob_sds)

# (12) robust log linear (abundance) regression scaled weighted (log_lmRob_sds_w)

# 12 rows of results = 12 types of regression over one time period (tp3, i.e. 3 generations) for each population

library(MASS) # weighted regression command included in here

library(robust) # for lmRob() function

pick.pops <- function(data2, Species, Pop_int, GenT)

{

results_mat<-NULL

MinY <- c()

MaxY <- c()

Area_Des <- c()

iter <- 0

for (i in Pop_int){

iter <- iter + 1

data3 <- subset(data2, data2$AREAID==i) # organize by population Area ID

res1 <- reg.rev1(data3, Species, i, GenT, paste(Species,"_",i,"_",sep=""))

results_mat <- rbind(results_mat,res1)

# for each species, multiple population results are added in sequential order in output

# column labels for output data matrix

Spp_Common_Name <- rep(Species_com, 12*length(Pop_int))

Species_Type <- rep(Stype, 12*length(Pop_int))

Area_ID <- rep(Pop_int, each=12)

Area_Des[iter] <- as.character(data3$AreaDes)

GenTime <- rep(GenT, 12*length(Pop_int))

print(as.numeric(tapply(data3$YearRead, data3$AREAID, min)))

MinY[iter] <- as.numeric(tapply(data3$YearRead, data3$AREAID, min))

MaxY[iter] <- as.numeric(tapply(data3$YearRead, data3$AREAID, max))

}

Area_Des <- rep(Area_Des, each=12)

MinYearPop <- rep(MinY, each=12)

MaxYearPop <- rep(MaxY, each=12)

TimePeriod_Reg <- rep(rep(3, each=12), length(Pop_int)) # repeat 3 (time period) 12 times (one for each regression kind) for each time period

RegType <- rep(c("lm_ns", "lm_ns_w", "lm_sds", "lm_sds_w", "lmRob_sds", "lmRob_sds_w", "lmRob_ns", "lmRob_ns_w", "log_lm_sds", "log_lm_sds_w", "log_lmRob_sds", "log_lmRob_sds_w"), (1*length(Pop_int)))

# Or if pup count data, use the following

# RegType <- rep(c("pc_lm_ns", "pc_lm_ns_w", "pc_lm_sds", "pc_lm_sds_w", "pc_lmRob_sds", "pc_lmRob_sds_w","pc_lmRob_ns", "pc_lmRob_ns_w", "pc_log_lm_sds", "pc_log_lm_sds_w", "pc_log_lmRob_sds", "pc_log_lmRob_sds_w"), (1*length(Pop_int)))

results <- data.frame(Spp_Common_Name, Species_Type, Area_ID, Area_Des, GenTime, MinYearPop, MaxYearPop, TimePeriod_Reg, RegType, results_mat)

return(results)

}

###### FUNCTION FOR RUNNING REGRESSIONS reg.rev1

# calls function regressions4, which is specified later

# runs regressions over tp3 (3 generations)

# generates columns of NAs in matrix if data are insufficient to perform robust regression

# 2 types of x and y data

# (1) scaled data, simple linear regression,

# (2) raw x & y abundance values

# build data matrix (results mat) with 13 columns and 12 rows for each time period, and specify inputs for each column

# will be added on to previously specified columns for each population for each species

# coef[2,] gives 1st 4 values, coef[1,1] gives intercept, no values get NA

# last two columns give bias test pvals for Mest and LSest

# Rows:

# (1) linear non-scaled (lm_ns),

# (2) linear non-scaled weighted (lm_ns_w),

# (3) linear scaled (lm_sds),

# (4) linear scaled weighted (lm_sds_w),

# (5) robust scaled (lmRob_sds),

# (6) robust scaled weighted (lmRob_sds_w),

# (7) robust non-scaled (lmRob_ns),

# (8) robust non-scaled weighted (lmRob_ns_w)

# (9) log linear (abundance) regression scaled non-weighted (log_lm_sds)

# (10) log linear (abundance) regression scaled weighted (log_lm_sds_w)

# (11) robust log linear (abundance) regression scaled non-weighted (log_lmRob_sds)

# (12) robust log linear (abundance) regression scaled weighted (log_lmRob_sds_w)

reg.rev1 <- function(data3, Species, Pop_int, GenT, pdf_prefix)

{

years <- data3$YearRead

maxyear <- max(years)

three_gen <- maxyear - (3*GenT)

time_per3 <- years >= three_gen & years <= maxyear

if ((sum(time_per3) > 2) & (length(unique(years[time_per3])) > 2))

# use PupCountMean instead of AbundMean, ACID_w_pc instead of ACID_w, and PCAbundCID instead of AbundCID in this section if using pup count data

# produce scaled x and y, regular x and y, and ACID weight

{ y_lm_scale <- (data3$AbundMean[time_per3] - mean(data3$AbundMean[time_per3]))/(sqrt(var(data3$AbundMean[time_per3])))

y_log <- log(data3$AbundMean[time_per3])

y_log_scale <- (y_log - mean(y_log))/(sqrt(var(y_log)))

x_lm_scale <- (data3$YearRead[time_per3] - mean(data3$YearRead[time_per3]))/(sqrt(var(data3$YearRead[time_per3])))

y_ns <- data3$AbundMean[time_per3]

x_ns <- data3$YearRead[time_per3]

ACID_w <- 1/(data3$AbundLCID[time_per3]^2)

print(y_lm_scale)

print(y_log)

print(y_log_scale)

print(x_lm_scale)

print(y_ns)

print(x_ns)

print(ACID_w)

# RUN regressions

reg_ns <- lm(y_ns ~ x_ns)

reg_ns_w <- lm(y_ns ~ x_ns, weights = ACID_w)

reg_sds <- lm(y_lm_scale ~ x_lm_scale)

reg_sds_w <- lm(y_lm_scale ~ x_lm_scale, weights = ACID_w)

rreg_sds <- lmRob(y_lm_scale ~ x_lm_scale)

rreg_sds_w <- lmRob(y_lm_scale ~ x_lm_scale, weights = ACID_w)

rreg_ns <- lmRob(y_ns ~ x_ns)

rreg_ns_w <- lmRob(y_ns ~ x_ns, weights = ACID_w)

log_reg_sds <- lm(y_log_scale ~ x_lm_scale)

log_reg_sds_w <- lm(y_log_scale ~ x_lm_scale, weights = ACID_w)

log_rreg_sds <- lmRob(y_log_scale ~ x_lm_scale)

log_rreg_sds_w <- lmRob(y_log_scale ~ x_lm_scale, weights = ACID_w)

results_mat <- matrix(0,12,13)

# create results matrix with various different columns headings

dimnames(results_mat) <- list(NULL, c("coeff_est", "SE", "tval", "pval", "CI95_Lower", "CI95_Upper", "RSq", "RSq_Adj", "sqrtRSq", "sqrtRSq_Adj", "Intercept", "Mest_biaspval", "LSest_biaspval"))

results_mat[1,] <- c(summary(reg_ns)$coef[2,], ((summary(reg_ns)$coef[2,1])-(1.96*(summary(reg_ns)$coef[2,2]))), ((summary(reg_ns)$coef[2,1])+(1.96*(summary(reg_ns)$coef[2,2]))), summary(reg_ns)$r.squared, summary(reg_ns)$adj.r.squared, sqrt(abs(summary(reg_ns)$r.squared)), sqrt(abs(summary(reg_ns)$adj.r.squared)), summary(reg_ns)$coef[1,1], NA, NA)

results_mat[2,] <- c(summary(reg_ns_w)$coef[2,], ((summary(reg_ns_w)$coef[2,1])-(1.96*(summary(reg_ns_w)$coef[2,2]))), ((summary(reg_ns_w)$coef[2,1])+(1.96*(summary(reg_ns_w)$coef[2,2]))), summary(reg_ns_w)$r.squared, summary(reg_ns_w)$adj.r.squared, sqrt(abs(summary(reg_ns_w)$r.squared)), sqrt(abs(summary(reg_ns_w)$adj.r.squared)), summary(reg_ns_w)$coef[1,1], NA, NA)

results_mat[3,] <- c(summary(reg_sds)$coef[2,], ((summary(reg_sds)$coef[2,1])-(1.96*(summary(reg_sds)$coef[2,2]))), ((summary(reg_sds)$coef[2,1])+(1.96*(summary(reg_sds)$coef[2,2]))), summary(reg_sds)$r.squared, summary(reg_sds)$adj.r.squared, sqrt(abs(summary(reg_sds)$r.squared)), sqrt(abs(summary(reg_sds)$adj.r.squared)), summary(reg_sds)$coef[1,1], NA, NA)

results_mat[4,] <- c(summary(reg_sds_w)$coef[2,], ((summary(reg_sds_w)$coef[2,1])-(1.96*(summary(reg_sds_w)$coef[2,2]))), ((summary(reg_sds_w)$coef[2,1])+(1.96*(summary(reg_sds_w)$coef[2,2]))), summary(reg_sds_w)$r.squared, summary(reg_sds_w)$adj.r.squared, sqrt(abs(summary(reg_sds_w)$r.squared)), sqrt(abs(summary(reg_sds_w)$adj.r.squared)), summary(reg_sds_w)$coef[1,1], NA, NA)

results_mat[5,] <- c(summary(rreg_sds)$coef[2,], ((summary(rreg_sds)$coef[2,1])-(1.96*(summary(rreg_sds)$coef[2,2]))), ((summary(rreg_sds)$coef[2,1])+(1.96*(summary(rreg_sds)$coef[2,2]))), summary(rreg_sds)$r.squared, NA, sqrt(abs(summary(rreg_sds)$r.squared)), NA, summary(rreg_sds)$coef[1,1], summary(rreg_sds)$biasTest[1,2], summary(rreg_sds)$biasTest[2,2])

results_mat[6,] <- c(summary(rreg_sds_w)$coef[2,], ((summary(rreg_sds_w)$coef[2,1])-(1.96*(summary(rreg_sds_w)$coef[2,2]))), ((summary(rreg_sds_w)$coef[2,1])+(1.96*(summary(rreg_sds_w)$coef[2,2]))), summary(rreg_sds_w)$r.squared, NA, sqrt(abs(summary(rreg_sds_w)$r.squared)), NA, summary(rreg_sds_w)$coef[1,1], summary(rreg_sds_w)$biasTest[1,2], summary(rreg_sds_w)$biasTest[2,2])

results_mat[7,] <- c(summary(rreg_ns)$coef[2,], ((summary(rreg_ns)$coef[2,1])-(1.96*(summary(rreg_ns)$coef[2,2]))), ((summary(rreg_ns)$coef[2,1])+(1.96*(summary(rreg_ns)$coef[2,2]))), summary(rreg_ns)$r.squared, NA, sqrt(abs(summary(rreg_ns)$r.squared)), NA, summary(rreg_ns)$coef[1,1], summary(rreg_ns)$biasTest[1,2], summary(rreg_ns)$biasTest[2,2])

results_mat[8,] <- c(summary(rreg_ns_w)$coef[2,], ((summary(rreg_ns_w)$coef[2,1])-(1.96*(summary(rreg_ns_w)$coef[2,2]))), ((summary(rreg_ns_w)$coef[2,1])+(1.96*(summary(rreg_ns_w)$coef[2,2]))), summary(rreg_ns_w)$r.squared, NA, sqrt(abs(summary(rreg_ns_w)$r.squared)), NA, summary(rreg_ns_w)$coef[1,1], summary(rreg_ns_w)$biasTest[1,2], summary(rreg_ns_w)$biasTest[2,2])

results_mat[9,] <- c(summary(log_reg_sds)$coef[2,], ((summary(log_reg_sds)$coef[2,1])-(1.96*(summary(log_reg_sds)$coef[2,2]))), ((summary(log_reg_sds)$coef[2,1])+(1.96*(summary(log_reg_sds)$coef[2,2]))), summary(log_reg_sds)$r.squared, summary(log_reg_sds)$adj.r.squared, sqrt(abs(summary(log_reg_sds)$r.squared)), sqrt(abs(summary(log_reg_sds)$adj.r.squared)), summary(log_reg_sds)$coef[1,1], NA, NA)

results_mat[10,] <- c(summary(log_reg_sds_w)$coef[2,], ((summary(log_reg_sds_w)$coef[2,1])-(1.96*(summary(log_reg_sds_w)$coef[2,2]))), ((summary(log_reg_sds_w)$coef[2,1])+(1.96*(summary(log_reg_sds_w)$coef[2,2]))), summary(log_reg_sds_w)$r.squared, summary(log_reg_sds_w)$adj.r.squared, sqrt(abs(summary(log_reg_sds_w)$r.squared)), sqrt(abs(summary(log_reg_sds_w)$adj.r.squared)), summary(log_reg_sds_w)$coef[1,1], NA, NA)

results_mat[11,] <- c(summary(log_rreg_sds)$coef[2,], ((summary(log_rreg_sds)$coef[2,1])-(1.96*(summary(log_rreg_sds)$coef[2,2]))), ((summary(log_rreg_sds)$coef[2,1])+(1.96*(summary(log_rreg_sds)$coef[2,2]))), summary(log_rreg_sds)$r.squared, NA, sqrt(abs(summary(log_rreg_sds)$r.squared)), NA, summary(log_rreg_sds)$coef[1,1], summary(log_rreg_sds)$biasTest[1,2], summary(log_rreg_sds)$biasTest[2,2])

results_mat[12,] <- c(summary(log_rreg_sds_w)$coef[2,], ((summary(log_rreg_sds_w)$coef[2,1])-(1.96*(summary(log_rreg_sds_w)$coef[2,2]))), ((summary(log_rreg_sds_w)$coef[2,1])+(1.96*(summary(log_rreg_sds_w)$coef[2,2]))), summary(log_rreg_sds_w)$r.squared, NA, sqrt(abs(summary(log_rreg_sds_w)$r.squared)), NA, summary(log_rreg_sds_w)$coef[1,1], summary(log_rreg_sds_w)$biasTest[1,2], summary(log_rreg_sds_w)$biasTest[2,2])

return(results_mat)

}

else {tp3_res <- matrix(NA,12,13)}

}

##### RUN ALL FUNCTIONS

spp_regdata <- pick.pops(data2, Species, Pop_int, GenT)

write.csv(spp_regdata, file=paste(Species,"_","table12regtp3",".csv", sep="")) # save matrix to a .csv

##### END
